# A first checklist of the Pteridophytes of Togo (West Africa)

**DOI:** 10.3897/BDJ.6.e24137

**Published:** 2018-06-06

**Authors:** Komla Elikplim Abotsi, Kouami Kokou, Jean-Yves Dubuisson, Germinal Rouhan

**Affiliations:** 1 Laboratory of Forestry Research, University of Lomé, Lomé, Togo; 2 Institut Systématique Evolution Biodiversité (ISYEB), Sorbonne Université, MNHN, CNRS, EPHE, 57 rue Cuvier CP 39, Paris, France; 3 Institut Systématique Evolution Biodiversité (ISYEB), Muséum national d'Histoire naturelle, CNRS, Sorbonne Université, EPHE, 57 rue Cuvier CP 39, Paris, France

**Keywords:** Checklist, Biodiversity, Ferns, Lycopods, Pteridophytes, Togo, West Africa

## Abstract

**Background:**

The present work proposes, for the first time, a study exclusively focused on the diversity of Pteridophytes in Togo.

The study was based on fieldwork that resulted in 869 new collections gathered between 2013 and 2017 in the country and on yet existing herbarium specimens kept at the Herbaria of Lomé and Paris. Thus, a total number of 1092 specimens collected throughout the country served as a basis for this work, to which were added the known, published occurrences of Pteridophytes for the country.

**New information:**

At the end of this study, a total diversity of 134 species belonging to 53 genera and 25 families and 12 orders were recorded and documented for the country. It results in 73 newly cited species for Togolese flora, including 61 spontaneous species. Lycopodiopsida (18 species) and Polypodiopsida (116 species) classes are both represented. The Polypodiales order is the most represented with 97 species. The Pteridaceae and Aspleniaceae families are the most diverse with 24 and 22 species respectively. Finally, notes were provided on species distribution at national level.

## Introduction

Much work has been done on the flora of Togo (Adjossou and Kokou 2009, Akpagana and Bouchet 1994, Batawila et al. 2005, Brunel et al. 1984, Guyot et al. 1994, Kokou et al. 1999, Kokou et al. 2006, Woegan 2007), resulting in flattering numbers of over 4000 species (SCBD 2018) for this relatively small country in tropical West Africa. Nevertheless, most of these works have mostly focused on angiosperms, suggesting an underestimation of other vascular plant lineages (the seed-bearing Gymnosperms and free-sporing 'Pteridophytes') and non vascular lineages ('Bryophytes').

'Pteridophytes' comprise actually two distinct, non-closely related evolutionary lineages treated as two classes, namely Lycophytes (Lycopodiopsida) and Ferns (Polypodiopsida) with a worldwide diversity estimated at about 11,916 species (PPGI 2016). On the African continent, the diversity of this group is relatively much lower than in the other tropical zones of the world with only 1,441 species (Roux 2009). This lower diversity could be linked, amongst other things, to the adverse past eco-climatic conditions and the weakness of the current forest cover (Kornas 1993), but it is certainly not the result of a single cause Couvreur 2015. Nevertheless, some regions are more diverse than others due to particular ecological conditions. These are mainly mountainous areas, islands and wet forest areas (Moran 1995, Aldasoro et al. 2004, Watkins Jr et al. 2006). Thus, the works of Aldasoro et al. (2004) have highlighted Togo Mountains as a hotspot of diversity for Pteridophytes on the African continent.

Nevertheless, apart from the works of Alston (1959), Roux (2009) and Abotsi et al. (2015), no other published taxonomic paper for Pteridophytes is available for the country. Moreover, those studies are still partial and cannot provide currently sufficient knowledge on the country. So, what is the current diversity of Pteridophytes in Togo? Available sources cite 61 species (Alston 1959), 54 species (Roux 2009) and even 126 species (Fousseni et al. 2014). It is therefore possible that some or all of these data are not accurate, meaning that there might be additional taxa still unknown for the local or even global flora. Conversely, some taxa may have been mis-stated for the country.

In the current era of the crisis of global biodiversity (Teyssèdre 2004) and increasing deforestation in Togo (Kokou 1998, Nadjombe 1992, Wilson et al. 2002) accompanied by a strong genetic erosion, it becomes more than urgent to know more about local biodiversity in order to draw the best conclusions for science and sustainable development. Species of great ecological, economic and/or genetic importance might be discovered and valued for the common good.

The present study therefore aims to produce an exhaustive and reliable list of Pteridophytes from Togo, based on reference herbarium specimens collected throughout the country and the occurrence data available in the literature.

## Materials and methods

### Study area

**Figure 1.**
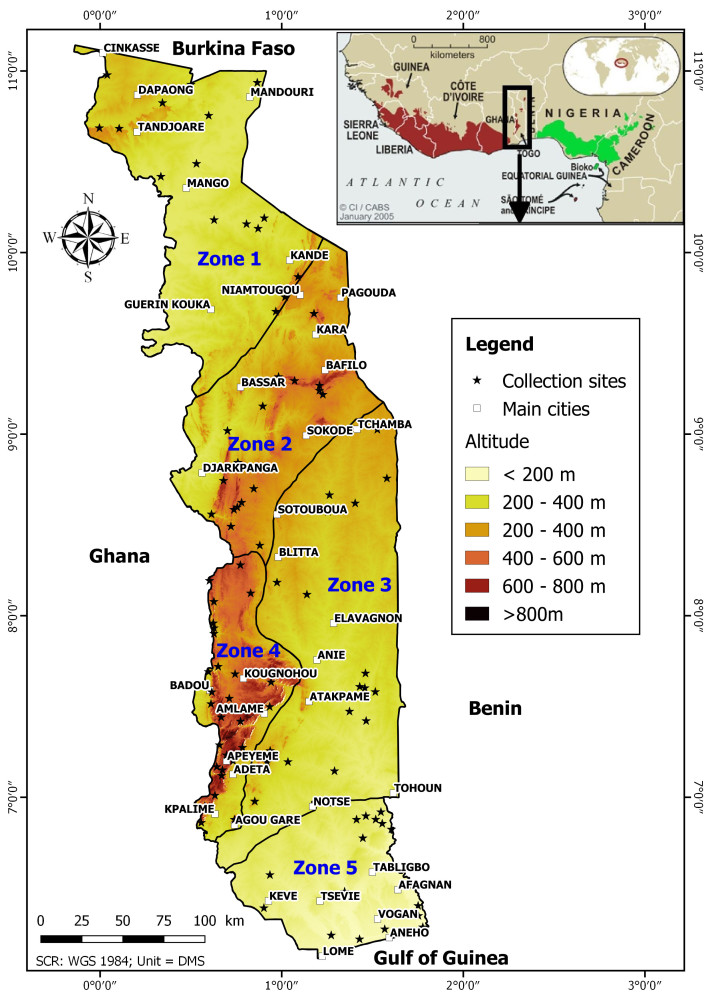
Study area (ecological subdivisions based on Ern (1979) and relief from SRTM2 scenes).

This study covers the whole of Togo. Togo is a country in West Africa, covering an area of 56,600 km². Ecologically, the country is divided into 5 ecological zones, discriminated mainly on the basis of climate, vegetation and relief (Ern 1979). The country usually has a humid to subhumid tropical climate, with rainfall ranges from 800 to 1600 mm per year. The country is crossed from south-west to north-east by the Atakora mountain range (also called Togo Mountains) which rises to 986 m at Mount Agou (Fig. 1). The rest of the territory consists of alluvial plains covered by a fairly dense network of mainly temporary rivers.

### Methodology

**Existing collections and occurrence data**

The herbarium specimens of Pteridophytes kept in the Lomé Herbarium (TOGO; herbarium acronym following Thiers 2018) were used for this study. In total, there are 223 specimens, which are judged insufficient to support this study. To this end, it was critical to carry out new collections in the field. In addition, the occurrences available at GBIF.org (2016) online database and in the works of Roux (2009) and Alston (1959) were taken into account.

**Fieldwork and collection of new specimens**

Complementary specimens were collected throughout the territory between March 2013 and August 2017 by K.E. Abotsi. The collection sites (Fig. 1) were chosen in order to increase the probability of encountering Pteridophytes and to cover the entire heterogeneity of the country. In all, 100 sites were selected in which collections were carried out, regardless of taxonomic affiliation.

For each gathering, 1 to 4 duplicates were collected depending on the availability of fertile plant material and the state of the population. A fragment of lamina was also collected and preserved in silica gel (Gaudeul and Rouhan 2013). Photos of the entire plant as well as close-ups for identification (fertile parts, venation, hairs, scales etc.) were taken to support the identification. A total of 869 georeferenced specimens were collected and are now kept in the Togo National Herbarium (TOGO). Duplicates were sent to the Herbarium of the National Museum of Natural History of Paris (P).

**Study of collected specimen**

Identifications of the newly collected specimens were carried out thanks to the reference specimens of TOGO and P herbaria (MNHN 2017) as well as the flora of Cameroon (Tardieu-Blot 1964), Benin (Akoègninou et al. 2006) and Mascarene Islands (Autrey et al. 2008). Species names were updated to follow the PPGI classification (PPGI 2016), and considering synonymies included in the literature or Tropicos (Missouri Botanical Garden 2018) or Roux (Roux 2009).

The collected specimens were studied on the basis of morphological characters (vegetative and reproductive), adapted from the list of descriptors proposed by Abotsi et al. (2015) for the Pteridaceae family. Information on the ecology and geographical distribution has been reported directly in the field.

**Presentation of the checklist**

The checklist is organised by suprageneric taxon according to the current classification (PPGI 2016). Only 3 representative specimens examined were cited in the checklist to avoid overloading the text.

## Data resources

The data underpinning the analysis reported in this paper are deposited at GBIF, the Global Biodiversity Information, http://ipt.pensoft.net/resource?r=togo_pteridophytes&v=1.1 (Abotsi 2018). The dataset contains only new occurrences recorded during this study. Prior occurrence data from other collectors are already available at GBIF portal.

## Checklists

### Checklist

#### Lycopodiopsida

#### Isoëtales

#### Isoëtaceae

#### Isoetes melanotheca

Alston

##### Materials

- **Type status:** Other material. **Occurrence:** catalogNumber: 12217; recordNumber: 2039 bis; recordedBy: Akpagana, K.; **Taxon:** scientificName: Isoetes melanotheca Alston; namePublishedIn: Bol. Soc. Broter. Ser. 2A, 30: 15 (1956); kingdom: Plantae; phylum: Pteridophyta; class: Lycopodiopsida; order: Isoëtales; family: Isoëtaceae; genus: Isoetes; specificEpithet: melanotheca; scientificNameAuthorship: Alston; **Location:** continent: Africa; country: Togo; countryCode: TG; locality: Akodessewa; verbatimSRS: WGS84; decimalLatitude: 7.4333333; decimalLongitude: 1.4166667; geodeticDatum: WGS84; **Identification:** identifiedBy: K. Akpagana; dateIdentified: 08-01-89; **Event:** eventDate: 08-01-89; habitat: Flooded meadow; **Record Level:** institutionID: Herbarium Togoense; collectionID: Akpagana, K.; institutionCode: TOGO; basisOfRecord: Preserved specimen
- **Type status:** Other material. **Occurrence:** catalogNumber: 12218; recordNumber: 55; recordedBy: Kokou, K.; **Taxon:** scientificName: Isoetes melanotheca Alston; namePublishedIn: Bol. Soc. Broter. Ser. 2A, 30: 15 (1956); kingdom: Plantae; phylum: Pteridophyta; class: Lycopodiopsida; order: Isoëtales; family: Isoëtaceae; genus: Isoetes; specificEpithet: melanotheca; scientificNameAuthorship: Alston; **Location:** continent: Africa; country: Togo; countryCode: TG; locality: Akodessewa; verbatimSRS: WGS84; decimalLatitude: 7.4333333; decimalLongitude: 1.4166667; geodeticDatum: WGS84; **Identification:** identifiedBy: Kokou, K.; dateIdentified: 11-07-89; **Event:** eventDate: 11-07-89; habitat: Flooded meadow; **Record Level:** institutionID: Herbarium Togoense; collectionID: Kokou, K.; institutionCode: TOGO; basisOfRecord: Preserved specimen
- **Type status:** Other material. **Occurrence:** catalogNumber: 12219; recordNumber: 2021; recordedBy: Akpagana, K.; **Taxon:** scientificName: Isoetes melanotheca Alston; namePublishedIn: Bol. Soc. Broter. Ser. 2A, 30: 15 (1956); kingdom: Plantae; phylum: Pteridophyta; class: Lycopodiopsida; order: Isoëtales; family: Isoëtaceae; genus: Isoetes; specificEpithet: melanotheca; scientificNameAuthorship: Alston; **Location:** continent: Africa; country: Togo; countryCode: TG; locality: Akodessewa; verbatimSRS: WGS84; decimalLatitude: 7.4333333; decimalLongitude: 1.4166667; geodeticDatum: WGS84; **Identification:** identifiedBy: K. Akpagana; dateIdentified: 07-01-89; **Event:** eventDate: 07-01-89; habitat: Flooded meadow; **Record Level:** institutionID: Herbarium Togoense; collectionID: Akpagana, K.; institutionCode: TOGO; basisOfRecord: Preserved specimen

##### Ecological interactions

###### Native status

Native

##### Distribution

Zone 3

#### Lycopodiales

#### Lycopodiaceae

#### Phlegmariurus staudtii

(Nessel) A.R.Field & Bostock

##### Materials

- **Type status:** Other material. **Occurrence:** catalogNumber: 12361; recordNumber: 5517; recordedBy: Brunel, J.-F.; **Taxon:** scientificName: Phlegmariurus staudtii (Nessel) A.R.Field & Bostock; namePublishedIn: PhytoKeys 20: 47 (2013) [epublished]; kingdom: Plantae; phylum: Pteridophyta; class: Lycopodiopsida; order: Lycopodiales; family: Lycopodiaceae; genus: Phlegmariurus; specificEpithet: staudtii; scientificNameAuthorship: (Nessel) A.R.Field & Bostock; **Location:** continent: Africa; country: Togo; countryCode: TG; locality: Danyi Elavagnon; verbatimElevation: 779; verbatimSRS: WGS84; decimalLatitude: 7.294897; decimalLongitude: 0.716599; geodeticDatum: WGS84; **Identification:** identifiedBy: Brunel, J.-F.; dateIdentified: /10/1978; **Event:** eventDate: /10/1978; habitat: Rainforest; **Record Level:** institutionID: Herbarium Togoense; collectionID: Brunel, J.-F.; institutionCode: TOGO; basisOfRecord: Preserved specimen

##### Ecological interactions

###### Native status

Native

##### Distribution

Zone 4

#### Palhinhaea cernua

(L.) Carv. Vasc. & Franco

##### Materials

- **Type status:** Other material. **Occurrence:** recordNumber: AB0345; recordedBy: Abotsi, K.E.; **Taxon:** scientificName: Palhinhaea cernua (L.) Carv. Vasc. & Franco; namePublishedIn: Bol. Soc. Broter. Ser. 2, 41: 25 (1967); kingdom: Plantae; phylum: Pteridophyta; class: Lycopodiopsida; order: Lycopodiales; family: Lycopodiaceae; genus: Palhinhaea; specificEpithet: cernua; scientificNameAuthorship: (L.) Carv. Vasc. & Franco; **Location:** continent: Africa; country: Togo; countryCode: TG; locality: Danyi Dzédramégan; verbatimElevation: 695; verbatimSRS: WGS84; decimalLatitude: 7.201429; decimalLongitude: 0.634892; geodeticDatum: WGS84; **Identification:** identifiedBy: Abotsi, K.E.; dateIdentified: 42643; **Event:** eventDate: 09-30-16; habitat: Wooded savannah; **Record Level:** institutionID: Herbarium Togoense; collectionID: Abotsi, K.E.; institutionCode: TOGO; basisOfRecord: Preserved specimen
- **Type status:** Other material. **Occurrence:** recordNumber: AB0457; recordedBy: Abotsi, K.E.; **Taxon:** scientificName: Palhinhaea cernua (L.) Carv. Vasc. & Franco; namePublishedIn: Bol. Soc. Broter. Ser. 2, 41: 25 (1967); kingdom: Plantae; phylum: Pteridophyta; class: Lycopodiopsida; order: Lycopodiales; family: Lycopodiaceae; genus: Palhinhaea; specificEpithet: cernua; scientificNameAuthorship: (L.) Carv. Vasc. & Franco; **Location:** continent: Africa; country: Togo; countryCode: TG; locality: Alédjo; verbatimElevation: 634; verbatimSRS: WGS84; decimalLatitude: 9.256244; decimalLongitude: 1.223654; geodeticDatum: WGS84; **Identification:** identifiedBy: Abotsi, K.E.; dateIdentified: 01-04-17; **Event:** eventDate: 01-04-17; habitat: Wooded savannah; **Record Level:** institutionID: Herbarium Togoense; collectionID: Abotsi, K.E.; institutionCode: TOGO; basisOfRecord: Preserved specimen
- **Type status:** Other material. **Occurrence:** recordNumber: ASM 0147; recordedBy: Abotsi, K.E., Sodjinou E. & Mingou P.; **Taxon:** scientificName: Palhinhaea cernua (L.) Carv. Vasc. & Franco; namePublishedIn: Bol. Soc. Broter. Ser. 2, 41: 25 (1967); kingdom: Plantae; phylum: Pteridophyta; class: Lycopodiopsida; order: Lycopodiales; family: Lycopodiaceae; genus: Palhinhaea; specificEpithet: cernua; scientificNameAuthorship: (L.) Carv. Vasc. & Franco; **Location:** continent: Africa; country: Togo; countryCode: TG; locality: Dikpéléou; verbatimElevation: 666; verbatimSRS: WGS84; decimalLatitude: 8.184573745; decimalLongitude: 0.634673418; geodeticDatum: WGS84; **Identification:** identifiedBy: Abotsi, K.E.; dateIdentified: 05-09-13; **Event:** eventDate: 05-09-13; habitat: Wooded savannah; **Record Level:** institutionID: Herbarium Togoense; collectionID: Abotsi, K.E.; institutionCode: TOGO; basisOfRecord: Preserved specimen

##### Ecological interactions

###### Native status

Native

##### Distribution

Zones 2 and 4

#### Pseudolycopodiella affinis

(Bory) Holub

##### Materials

- **Type status:** Other material. **Occurrence:** catalogNumber: 12360; recordNumber: 4661; recordedBy: Brunel, J.-F.; **Taxon:** scientificName: Pseudolycopodiella affinis (Bory) Holub.; namePublishedIn: Folia Geobot. Phytotax. 20 (1): 79 (1985); kingdom: Plantae; phylum: Pteridophyta; class: Lycopodiopsida; order: Lycopodiales; family: Lycopodiaceae; genus: Lycopodiella; specificEpithet: affinis; scientificNameAuthorship: (Bory) Holub.; **Location:** continent: Africa; country: Togo; countryCode: TG; locality: Défalé; verbatimElevation: 272; verbatimSRS: WGS84; decimalLatitude: 9.934112; decimalLongitude: 1.086934; geodeticDatum: WGS84; **Identification:** identifiedBy: Brunel, J.-F.; dateIdentified: /1978; **Event:** eventDate: /1978; habitat: Flooded meadow; **Record Level:** institutionID: Herbarium Togoense; collectionID: Brunel, J.-F.; institutionCode: TOGO; basisOfRecord: Preserved specimen

##### Ecological interactions

###### Native status

Native

##### Distribution

Zone 2

#### Selaginellales

#### Selaginellaceae

#### Selaginella blepharophylla

Alston

##### Materials

- **Type status:** Other material. **Occurrence:** recordNumber: AB0181; recordedBy: Abotsi, K.E.; **Taxon:** scientificName: Selaginella blepharophylla Alston; namePublishedIn: Mém. Inst. Fr. Afr. Noire 50: 40, t. 6, ff. 9-15 (1957); kingdom: Plantae; phylum: Pteridophyta; class: Lycopodiopsida; order: Selaginellales; family: Selaginellaceae; genus: Selaginella; specificEpithet: blepharophylla; scientificNameAuthorship: Alston; **Location:** continent: Africa; country: Togo; countryCode: TG; locality: Womé; verbatimElevation: 319; verbatimSRS: WGS84; decimalLatitude: 6.857785; decimalLongitude: 0.554402; geodeticDatum: WGS84; **Identification:** identifiedBy: Abotsi, K.E.; dateIdentified: 08-03-16; **Event:** eventDate: 08-03-16; habitat: Rainforest; **Record Level:** institutionID: Herbarium Togoense; collectionID: Abotsi, K.E.; institutionCode: TOGO; basisOfRecord: Preserved specimen
- **Type status:** Other material. **Occurrence:** recordNumber: AB0572; recordedBy: Abotsi, K.E.; **Taxon:** scientificName: Selaginella blepharophylla Alston; namePublishedIn: Mém. Inst. Fr. Afr. Noire 50: 40, t. 6, ff. 9-15 (1957); kingdom: Plantae; phylum: Pteridophyta; class: Lycopodiopsida; order: Selaginellales; family: Selaginellaceae; genus: Selaginella; specificEpithet: blepharophylla; scientificNameAuthorship: Alston; **Location:** continent: Africa; country: Togo; countryCode: TG; locality: Tchatchakou; verbatimElevation: 230; verbatimSRS: WGS84; decimalLatitude: 8.573536; decimalLongitude: 0.616629; geodeticDatum: WGS84; **Identification:** identifiedBy: Abotsi, K.E.; dateIdentified: 07-15-17; **Event:** eventDate: 07-15-17; habitat: Dry dense forest; **Record Level:** institutionID: Herbarium Togoense; collectionID: Abotsi, K.E.; institutionCode: TOGO; basisOfRecord: Preserved specimen
- **Type status:** Other material. **Occurrence:** recordNumber: AB0428; recordedBy: Abotsi, K.E.; **Taxon:** scientificName: Selaginella blepharophylla Alston; namePublishedIn: Mém. Inst. Fr. Afr. Noire 50: 40, t. 6, ff. 9-15 (1957); kingdom: Plantae; phylum: Pteridophyta; class: Lycopodiopsida; order: Selaginellales; family: Selaginellaceae; genus: Selaginella; specificEpithet: blepharophylla; scientificNameAuthorship: Alston; **Location:** continent: Africa; country: Togo; countryCode: TG; locality: Danyi Dzogbégan; verbatimElevation: 719; verbatimSRS: WGS84; decimalLatitude: 7.231714; decimalLongitude: 0.677706; geodeticDatum: WGS84; **Identification:** identifiedBy: Abotsi, K.E.; dateIdentified: 12-27-16; **Event:** eventDate: 12-27-16; habitat: Rainforest; **Record Level:** institutionID: Herbarium Togoense; collectionID: Abotsi, K.E.; institutionCode: TOGO; basisOfRecord: Preserved specimen

##### Ecological interactions

###### Native status

Native

##### Distribution

Zone 4

#### Selaginella buccholzii

Hieron.

##### Materials

- **Type status:** Other material. **Occurrence:** recordNumber: AB0553; recordedBy: Abotsi, K.E.; **Taxon:** scientificName: Selaginella buccholzii Hieron.; namePublishedIn: Engl. & Prantl, Nat. Pfl. 1(4): 696, no. 272 (1901 publ. 1902); kingdom: Plantae; phylum: Pteridophyta; class: Lycopodiopsida; order: Selaginellales; family: Selaginellaceae; genus: Selaginella; specificEpithet: buccholzii; scientificNameAuthorship: Hieron.; **Location:** continent: Africa; country: Togo; countryCode: TG; locality: Alédjo; verbatimElevation: 512; verbatimSRS: WGS84; decimalLatitude: 9.271784; decimalLongitude: 1.207243; geodeticDatum: WGS84; **Identification:** identifiedBy: Abotsi, K.E.; dateIdentified: 07-12-17; **Event:** eventDate: 07-12-17; habitat: Dry dense forest; **Record Level:** institutionID: Herbarium Togoense; collectionID: Abotsi, K.E.; institutionCode: TOGO; basisOfRecord: Preserved specimen
- **Type status:** Other material. **Occurrence:** recordNumber: AB0557; recordedBy: Abotsi, K.E.; **Taxon:** scientificName: Selaginella buccholzii Hieron.; namePublishedIn: Engl. & Prantl, Nat. Pfl. 1(4): 696, no. 272 (1901 publ. 1902); kingdom: Plantae; phylum: Pteridophyta; class: Lycopodiopsida; order: Selaginellales; family: Selaginellaceae; genus: Selaginella; specificEpithet: buccholzii; scientificNameAuthorship: Hieron.; **Location:** continent: Africa; country: Togo; countryCode: TG; locality: Efolo; verbatimElevation: 626; verbatimSRS: WGS84; decimalLatitude: 9.289027; decimalLongitude: 1.079214; geodeticDatum: WGS84; **Identification:** identifiedBy: Abotsi, K.E.; dateIdentified: 07-13-17; **Event:** eventDate: 07-13-17; habitat: Woodland; **Record Level:** institutionID: Herbarium Togoense; collectionID: Abotsi, K.E.; institutionCode: TOGO; basisOfRecord: Preserved specimen
- **Type status:** Other material. **Occurrence:** recordNumber: AB0558; recordedBy: Abotsi, K.E.; **Taxon:** scientificName: Selaginella buccholzii Hieron.; namePublishedIn: Engl. & Prantl, Nat. Pfl. 1(4): 696, no. 272 (1901 publ. 1902); kingdom: Plantae; phylum: Pteridophyta; class: Lycopodiopsida; order: Selaginellales; family: Selaginellaceae; genus: Selaginella; specificEpithet: buccholzii; scientificNameAuthorship: Hieron.; **Location:** continent: Africa; country: Togo; countryCode: TG; locality: Efolo; verbatimElevation: 627; verbatimSRS: WGS84; decimalLatitude: 9.288883; decimalLongitude: 1.079122; geodeticDatum: WGS84; **Identification:** identifiedBy: Abotsi, K.E.; dateIdentified: 07-13-17; **Event:** eventDate: 07-13-17; habitat: Woodland; **Record Level:** institutionID: Herbarium Togoense; collectionID: Abotsi, K.E.; institutionCode: TOGO; basisOfRecord: Preserved specimen

##### Ecological interactions

###### Native status

Native

##### Distribution

Zone 2

#### Selaginella cathedrifolia

Spring

##### Materials

- **Type status:** Other material. **Occurrence:** recordNumber: AB0265; recordedBy: Abotsi, K.E.; **Taxon:** scientificName: Selaginella cathedrifolia Spring; namePublishedIn: Monogr. Lyc. 2: 112, no. 58 (1850); kingdom: Plantae; phylum: Pteridophyta; class: Lycopodiopsida; order: Selaginellales; family: Selaginellaceae; genus: Selaginella; specificEpithet: cathedrifolia; scientificNameAuthorship: Spring; **Location:** continent: Africa; country: Togo; countryCode: TG; locality: Badou; verbatimElevation: 389; verbatimSRS: WGS84; decimalLatitude: 7.5807; decimalLongitude: 0.622406; geodeticDatum: WGS84; **Identification:** identifiedBy: Abotsi, K.E.; dateIdentified: 08-08-16; **Event:** eventDate: 08-08-16; habitat: Rainforest; **Record Level:** institutionID: Herbarium Togoense; collectionID: Abotsi, K.E.; institutionCode: TOGO; basisOfRecord: Preserved specimen
- **Type status:** Other material. **Occurrence:** recordNumber: AB0267; recordedBy: Abotsi, K.E.; **Taxon:** scientificName: Selaginella cathedrifolia Spring; namePublishedIn: Monogr. Lyc. 2: 112, no. 58 (1850); kingdom: Plantae; phylum: Pteridophyta; class: Lycopodiopsida; order: Selaginellales; family: Selaginellaceae; genus: Selaginella; specificEpithet: cathedrifolia; scientificNameAuthorship: Spring; **Location:** continent: Africa; country: Togo; countryCode: TG; locality: Okpahoué; verbatimElevation: 369; verbatimSRS: WGS84; decimalLatitude: 7.618456; decimalLongitude: 0.997671; geodeticDatum: WGS84; **Identification:** identifiedBy: Abotsi, K.E.; dateIdentified: 09-15-16; **Event:** eventDate: 09-15-16; habitat: Rainforest; **Record Level:** institutionID: Herbarium Togoense; collectionID: Abotsi, K.E.; institutionCode: TOGO; basisOfRecord: Preserved specimen
- **Type status:** Other material. **Occurrence:** recordNumber: AB0313; recordedBy: Abotsi, K.E.; **Taxon:** scientificName: Selaginella cathedrifolia Spring; namePublishedIn: Monogr. Lyc. 2: 112, no. 58 (1850); kingdom: Plantae; phylum: Pteridophyta; class: Lycopodiopsida; order: Selaginellales; family: Selaginellaceae; genus: Selaginella; specificEpithet: cathedrifolia; scientificNameAuthorship: Spring; **Location:** continent: Africa; country: Togo; countryCode: TG; locality: Assoukoko; verbatimElevation: 604; verbatimSRS: WGS84; decimalLatitude: 8.003763; decimalLongitude: 0.625499; geodeticDatum: WGS84; **Identification:** identifiedBy: Abotsi, K.E.; dateIdentified: 09-18-16; **Event:** eventDate: 09-18-16; habitat: Rainforest; **Record Level:** institutionID: Herbarium Togoense; collectionID: Abotsi, K.E.; institutionCode: TOGO; basisOfRecord: Preserved specimen

##### Ecological interactions

###### Native status

Native

##### Distribution

Zone 4

#### Selaginella goudotianavar.abyssinica

(Spring) Bizzarri

##### Materials

- **Type status:** Other material. **Occurrence:** recordNumber: AB0128; recordedBy: Abotsi, K.E.; **Taxon:** scientificName: Selaginella goudotiana Spring var. abyssinica (Spring) Bizzarri; namePublishedIn: Webbia 29: 585, fig. 9 (1975); kingdom: Plantae; phylum: Pteridophyta; class: Lycopodiopsida; order: Selaginellales; family: Selaginellaceae; genus: Selaginella; specificEpithet: goudotiana; infraspecificEpithet: abyssinica; taxonRank: Variety; scientificNameAuthorship: (Spring) Bizzarri; **Location:** continent: Africa; country: Togo; countryCode: TG; locality: Danyi N'Digbé; verbatimElevation: 590; verbatimSRS: WGS84; decimalLatitude: 7.115728; decimalLongitude: 0.661356; geodeticDatum: WGS84; **Identification:** identifiedBy: Abotsi, K.E.; dateIdentified: 06-28-16; **Event:** eventDate: 06-28-16; habitat: Coffee/cocoa based agroforest; **Record Level:** institutionID: Herbarium Togoense; collectionID: Abotsi, K.E.; institutionCode: TOGO; basisOfRecord: Preserved specimen
- **Type status:** Other material. **Occurrence:** recordNumber: AB0166; recordedBy: Abotsi, K.E.; **Taxon:** scientificName: Selaginella goudotiana Spring var. abyssinica (Spring) Bizzarri; namePublishedIn: Webbia 29: 585, fig. 9 (1975); kingdom: Plantae; phylum: Pteridophyta; class: Lycopodiopsida; order: Selaginellales; family: Selaginellaceae; genus: Selaginella; specificEpithet: goudotiana; infraspecificEpithet: abyssinica; taxonRank: Variety; scientificNameAuthorship: (Spring) Bizzarri; **Location:** continent: Africa; country: Togo; countryCode: TG; locality: Kpimé Séva; verbatimElevation: 309; verbatimSRS: WGS84; decimalLatitude: 7.00812; decimalLongitude: 0.645614; geodeticDatum: WGS84; **Identification:** identifiedBy: Abotsi, K.E.; dateIdentified: 07-23-16; **Event:** eventDate: 07-23-16; habitat: Rainforest; **Record Level:** institutionID: Herbarium Togoense; collectionID: Abotsi, K.E.; institutionCode: TOGO; basisOfRecord: Preserved specimen
- **Type status:** Other material. **Occurrence:** recordNumber: AB0499; recordedBy: Abotsi, K.E.; **Taxon:** scientificName: Selaginella goudotiana Spring var. abyssinica (Spring) Bizzarri; namePublishedIn: Webbia 29: 585, fig. 9 (1975); kingdom: Plantae; phylum: Pteridophyta; class: Lycopodiopsida; order: Selaginellales; family: Selaginellaceae; genus: Selaginella; specificEpithet: goudotiana; infraspecificEpithet: abyssinica; taxonRank: Variety; scientificNameAuthorship: (Spring) Bizzarri; **Location:** continent: Africa; country: Togo; countryCode: TG; locality: Gbadi N'Kugna; verbatimElevation: 674; verbatimSRS: WGS84; decimalLatitude: 7.454038; decimalLongitude: 0.705819; geodeticDatum: WGS84; **Identification:** identifiedBy: Abotsi, K.E.; dateIdentified: 04-25-17; **Event:** eventDate: 04-25-17; habitat: Rainforest; **Record Level:** institutionID: Herbarium Togoense; collectionID: Abotsi, K.E.; institutionCode: TOGO; basisOfRecord: Preserved specimen

##### Ecological interactions

###### Native status

Native

##### Distribution

Zone 4

#### Selaginella kalbreyeri

Baker

##### Materials

- **Type status:** Other material. **Occurrence:** recordNumber: AB0571; recordedBy: Abotsi, K.E.; **Taxon:** scientificName: Selaginella kalbreyeri Baker; namePublishedIn: J. Bot. 22: 276, no. 157 (1884); kingdom: Plantae; phylum: Pteridophyta; class: Lycopodiopsida; order: Selaginellales; family: Selaginellaceae; genus: Selaginella; specificEpithet: kalbreyeri; scientificNameAuthorship: Baker; **Location:** continent: Africa; country: Togo; countryCode: TG; locality: Tchatchakou; verbatimElevation: 213; verbatimSRS: WGS84; decimalLatitude: 8.573451; decimalLongitude: 0.616623; geodeticDatum: WGS84; **Identification:** identifiedBy: Abotsi, K.E.; dateIdentified: 07-15-17; **Event:** eventDate: 07-15-17; habitat: Dry dense forest; **Record Level:** institutionID: Herbarium Togoense; collectionID: Abotsi, K.E.; institutionCode: TOGO; basisOfRecord: Preserved specimen
- **Type status:** Other material. **Occurrence:** recordNumber: AB0566; recordedBy: Abotsi, K.E.; **Taxon:** scientificName: Selaginella kalbreyeri Baker; namePublishedIn: J. Bot. 22: 276, no. 157 (1884); kingdom: Plantae; phylum: Pteridophyta; class: Lycopodiopsida; order: Selaginellales; family: Selaginellaceae; genus: Selaginella; specificEpithet: kalbreyeri; scientificNameAuthorship: Baker; **Location:** continent: Africa; country: Togo; countryCode: TG; locality: Tchatchaminadè; verbatimElevation: 401; verbatimSRS: WGS84; decimalLatitude: 9.310482; decimalLongitude: 0.984611; geodeticDatum: WGS84; **Identification:** identifiedBy: Abotsi, K.E.; dateIdentified: 07-14-17; **Event:** eventDate: 07-14-17; habitat: Dry dense forest; **Record Level:** institutionID: Herbarium Togoense; collectionID: Abotsi, K.E.; institutionCode: TOGO; basisOfRecord: Preserved specimen

##### Ecological interactions

###### Native status

Native

##### Distribution

Zone 2

#### Selaginella molliceps

Spring

##### Materials

- **Type status:** Other material. **Occurrence:** recordNumber: AB0309; recordedBy: Abotsi, K.E.; **Taxon:** scientificName: Selaginella molliceps Spring; namePublishedIn: Mém. Acad. Roy. Sci. Belg. 24: 257 (1850); kingdom: Plantae; phylum: Pteridophyta; class: Lycopodiopsida; order: Selaginellales; family: Selaginellaceae; genus: Selaginella; specificEpithet: molliceps; scientificNameAuthorship: Spring; **Location:** continent: Africa; country: Togo; countryCode: TG; locality: Assoukoko; verbatimElevation: 656; verbatimSRS: WGS84; decimalLatitude: 8.005225; decimalLongitude: 0.628037; geodeticDatum: WGS84; **Identification:** identifiedBy: Abotsi, K.E.; dateIdentified: 09-18-16; **Event:** eventDate: 09-18-16; habitat: Rainforest; **Record Level:** institutionID: Herbarium Togoense; collectionID: Abotsi, K.E.; institutionCode: TOGO; basisOfRecord: Preserved specimen
- **Type status:** Other material. **Occurrence:** recordNumber: AB0016; recordedBy: Abotsi, K.E.; **Taxon:** scientificName: Selaginella molliceps Spring; namePublishedIn: Mém. Acad. Roy. Sci. Belg. 24: 257 (1850); kingdom: Plantae; phylum: Pteridophyta; class: Lycopodiopsida; order: Selaginellales; family: Selaginellaceae; genus: Selaginella; specificEpithet: molliceps; scientificNameAuthorship: Spring; **Location:** continent: Africa; country: Togo; countryCode: TG; locality: Agou Nyogbo; verbatimElevation: 555; verbatimSRS: WGS84; decimalLatitude: 6.876866; decimalLongitude: 0.733962; geodeticDatum: WGS84; **Identification:** identifiedBy: Abotsi, K.E.; dateIdentified: 06-13-16; **Event:** eventDate: 06-13-16; habitat: Coffee/cocoa based agroforest; **Record Level:** institutionID: Herbarium Togoense; collectionID: Abotsi, K.E.; institutionCode: TOGO; basisOfRecord: Preserved specimen

##### Ecological interactions

###### Native status

Native

##### Distribution

Zone 4

#### Selaginella myosurus

(Sw.) Alston

##### Materials

- **Type status:** Other material. **Occurrence:** recordNumber: AB0110; recordedBy: Abotsi, K.E.; **Taxon:** scientificName: Selaginella myosurus (Sw.) Alston; namePublishedIn: J. Bot. 70: 64, no. 6 (1932); kingdom: Plantae; phylum: Pteridophyta; class: Lycopodiopsida; order: Selaginellales; family: Selaginellaceae; genus: Selaginella; specificEpithet: myosurus; scientificNameAuthorship: (Sw.) Alston; **Location:** continent: Africa; country: Togo; countryCode: TG; locality: Danyi N'Digbé; verbatimElevation: 695; verbatimSRS: WGS84; decimalLatitude: 7.151191; decimalLongitude: 0.671984; geodeticDatum: WGS84; **Identification:** identifiedBy: Abotsi, K.E.; dateIdentified: 06-27-16; **Event:** eventDate: 06-27-16; habitat: Rainforest; **Record Level:** institutionID: Herbarium Togoense; collectionID: Abotsi, K.E.; institutionCode: TOGO; basisOfRecord: Preserved specimen
- **Type status:** Other material. **Occurrence:** recordNumber: AB0214; recordedBy: Abotsi, K.E.; **Taxon:** scientificName: Selaginella myosurus (Sw.) Alston; namePublishedIn: J. Bot. 70: 64, no. 6 (1932); kingdom: Plantae; phylum: Pteridophyta; class: Lycopodiopsida; order: Selaginellales; family: Selaginellaceae; genus: Selaginella; specificEpithet: myosurus; scientificNameAuthorship: (Sw.) Alston; **Location:** continent: Africa; country: Togo; countryCode: TG; locality: Kuma Dunyo; verbatimElevation: 614; verbatimSRS: WGS84; decimalLatitude: 6.981979; decimalLongitude: 0.596179; geodeticDatum: WGS84; **Identification:** identifiedBy: Abotsi, K.E.; dateIdentified: 08-04-16; **Event:** eventDate: 08-04-16; habitat: Rainforest; **Record Level:** institutionID: Herbarium Togoense; collectionID: Abotsi, K.E.; institutionCode: TOGO; basisOfRecord: Preserved specimen
- **Type status:** Other material. **Occurrence:** recordNumber: AB0483; recordedBy: Abotsi, K.E.; **Taxon:** scientificName: Selaginella myosurus (Sw.) Alston; namePublishedIn: J. Bot. 70: 64, no. 6 (1932); kingdom: Plantae; phylum: Pteridophyta; class: Lycopodiopsida; order: Selaginellales; family: Selaginellaceae; genus: Selaginella; specificEpithet: myosurus; scientificNameAuthorship: (Sw.) Alston; **Location:** continent: Africa; country: Togo; countryCode: TG; locality: Ediwlou; verbatimElevation: 618; verbatimSRS: WGS84; decimalLatitude: 7.40944; decimalLongitude: 0.680984; geodeticDatum: WGS84; **Identification:** identifiedBy: Abotsi, K.E.; dateIdentified: 04-25-17; **Event:** eventDate: 04-25-17; habitat: Rainforest; **Record Level:** institutionID: Herbarium Togoense; collectionID: Abotsi, K.E.; institutionCode: TOGO; basisOfRecord: Preserved specimen

##### Ecological interactions

###### Native status

Native

##### Distribution

Zone 4

#### Selaginella njamnjamensis

Hieron.

##### Materials

- **Type status:** Other material. **Occurrence:** recordNumber: AB0222; recordedBy: Abotsi, K.E.; **Taxon:** scientificName: Selaginella njamnjamensis Hieron.; namePublishedIn: Hedwigia 39: 312 (1900); kingdom: Plantae; phylum: Pteridophyta; class: Lycopodiopsida; order: Selaginellales; family: Selaginellaceae; genus: Selaginella; specificEpithet: njamnjamensis; scientificNameAuthorship: Hieron.; **Location:** continent: Africa; country: Togo; countryCode: TG; locality: Ounabè; verbatimElevation: 494; verbatimSRS: WGS84; decimalLatitude: 7.5603; decimalLongitude: 1.019597; geodeticDatum: WGS84; **Identification:** identifiedBy: Abotsi, K.E.; dateIdentified: 08-05-16; **Event:** eventDate: 08-05-16; habitat: Rainforest; **Record Level:** institutionID: Herbarium Togoense; collectionID: Abotsi, K.E.; institutionCode: TOGO; basisOfRecord: Preserved specimen
- **Type status:** Other material. **Occurrence:** recordNumber: AB0160; recordedBy: Abotsi, K.E.; **Taxon:** scientificName: Selaginella njamnjamensis Hieron.; namePublishedIn: Hedwigia 39: 312 (1900); kingdom: Plantae; phylum: Pteridophyta; class: Lycopodiopsida; order: Selaginellales; family: Selaginellaceae; genus: Selaginella; specificEpithet: njamnjamensis; scientificNameAuthorship: Hieron.; **Location:** continent: Africa; country: Togo; countryCode: TG; locality: Haito; verbatimElevation: 412; verbatimSRS: WGS84; decimalLatitude: 7.19919; decimalLongitude: 0.909572; geodeticDatum: WGS84; **Identification:** identifiedBy: Abotsi, K.E.; dateIdentified: 07-22-16; **Event:** eventDate: 07-22-16; habitat: Dry dense forest; **Record Level:** institutionID: Herbarium Togoense; collectionID: Abotsi, K.E.; institutionCode: TOGO; basisOfRecord: Preserved specimen

##### Ecological interactions

###### Native status

Native

##### Distribution

Zones 3 and 4

#### Selaginella protensa

Alston

##### Materials

- **Type status:** Other material. **Occurrence:** catalogNumber: 12422; recordNumber: 749 Bis; recordedBy: Brunel, J.-F.; **Taxon:** scientificName: Selaginella protensa Alston; namePublishedIn: Mém. Inst. Fr. Afr. Noire 50: 41 (1957); kingdom: Plantae; phylum: Pteridophyta; class: Lycopodiopsida; order: Selaginellales; family: Selaginellaceae; genus: Selaginella; specificEpithet: protensa; scientificNameAuthorship: Alston; **Location:** continent: Africa; country: Togo; countryCode: TG; locality: Kloto; verbatimElevation: 645; verbatimSRS: WGS84; decimalLatitude: 6.9415; decimalLongitude: 0.57489; geodeticDatum: WGS85; **Identification:** identifiedBy: Brunel, J.-F.; dateIdentified: /1972; **Event:** eventDate: /1972; habitat: Rainforest; **Record Level:** institutionID: Herbarium Togoense; collectionID: Brunel, J.-F.; institutionCode: TOGO; basisOfRecord: Preserved specimen

##### Ecological interactions

###### Native status

Native

##### Distribution

Zone 4

#### Selaginella soyauxii

Hieron.

##### Materials

- **Type status:** Other material. **Occurrence:** recordNumber: AB0207; recordedBy: Abotsi, K.E.; **Taxon:** scientificName: Selaginella soyauxii Hieron.; namePublishedIn: Engl. & Prantl, Nat. Pfl. 1(4): 697, no. 305. (1901 publ. 1902); kingdom: Plantae; phylum: Pteridophyta; class: Lycopodiopsida; order: Selaginellales; family: Selaginellaceae; genus: Selaginella; specificEpithet: soyauxii; scientificNameAuthorship: Hieron.; **Location:** continent: Africa; country: Togo; countryCode: TG; locality: Kpimé Séva; verbatimElevation: 527; verbatimSRS: WGS84; decimalLatitude: 7.017175; decimalLongitude: 0.64061; geodeticDatum: WGS84; **Identification:** identifiedBy: Abotsi, K.E.; dateIdentified: 08-04-16; **Event:** eventDate: 08-04-16; habitat: Rainforest; **Record Level:** institutionID: Herbarium Togoense; collectionID: Abotsi, K.E.; institutionCode: TOGO; basisOfRecord: Preserved specimen

##### Ecological interactions

###### Native status

Native

##### Distribution

Zone 4

#### Selaginella thomensis

Alston

##### Materials

- **Type status:** Other material. **Occurrence:** recordNumber: AB0208; recordedBy: Abotsi, K.E.; **Taxon:** scientificName: Selaginella thomensis Alston; namePublishedIn: Exell, Cat. Vasc. Pl. S. Tomé, 97, f. 3 (1944); kingdom: Plantae; phylum: Pteridophyta; class: Lycopodiopsida; order: Selaginellales; family: Selaginellaceae; genus: Selaginella; specificEpithet: thomensis; scientificNameAuthorship: Alston; **Location:** continent: Africa; country: Togo; countryCode: TG; locality: Kpimé Séva; verbatimElevation: 527; verbatimSRS: WGS84; decimalLatitude: 7.017048; decimalLongitude: 0.640631; geodeticDatum: WGS84; **Identification:** identifiedBy: Abotsi, K.E.; dateIdentified: 08-04-16; **Event:** eventDate: 08-04-16; habitat: Rainforest; **Record Level:** institutionID: Herbarium Togoense; collectionID: Abotsi, K.E.; institutionCode: TOGO; basisOfRecord: Preserved specimen
- **Type status:** Other material. **Occurrence:** recordNumber: AB0238; recordedBy: Abotsi, K.E.; **Taxon:** scientificName: Selaginella thomensis Alston; namePublishedIn: Exell, Cat. Vasc. Pl. S. Tomé, 97, f. 3 (1944); kingdom: Plantae; phylum: Pteridophyta; class: Lycopodiopsida; order: Selaginellales; family: Selaginellaceae; genus: Selaginella; specificEpithet: thomensis; scientificNameAuthorship: Alston; **Location:** continent: Africa; country: Togo; countryCode: TG; locality: Akloa; verbatimElevation: 477; verbatimSRS: WGS84; decimalLatitude: 7.515382; decimalLongitude: 0.620145; geodeticDatum: WGS84; **Identification:** identifiedBy: Abotsi, K.E.; dateIdentified: 08-06-16; **Event:** eventDate: 08-06-16; habitat: Rainforest; **Record Level:** institutionID: Herbarium Togoense; collectionID: Abotsi, K.E.; institutionCode: TOGO; basisOfRecord: Preserved specimen

##### Ecological interactions

###### Native status

Native

##### Distribution

Zone 4

#### Selaginella versicolor

Spring

##### Materials

- **Type status:** Other material. **Occurrence:** recordNumber: AB0216; recordedBy: Abotsi, K.E.; **Taxon:** scientificName: Selaginella versicolor Spring; namePublishedIn: Bull. Acad. Roy. Soc. Bruxelles 10: 143, no. 57 (1843); kingdom: Plantae; phylum: Pteridophyta; class: Lycopodiopsida; order: Selaginellales; family: Selaginellaceae; genus: Selaginella; specificEpithet: versicolor; scientificNameAuthorship: Spring; **Location:** continent: Africa; country: Togo; countryCode: TG; locality: Ayomé; verbatimElevation: 312; verbatimSRS: WGS84; decimalLatitude: 7.500341; decimalLongitude: 0.955395; geodeticDatum: WGS84; **Identification:** identifiedBy: Abotsi, K.E.; dateIdentified: 08-05-16; **Event:** eventDate: 08-05-16; habitat: Rainforest; **Record Level:** institutionID: Herbarium Togoense; collectionID: Abotsi, K.E.; institutionCode: TOGO; basisOfRecord: Preserved specimen
- **Type status:** Other material. **Occurrence:** recordNumber: AB0315; recordedBy: Abotsi, K.E.; **Taxon:** scientificName: Selaginella versicolor Spring; namePublishedIn: Bull. Acad. Roy. Soc. Bruxelles 10: 143, no. 57 (1843); kingdom: Plantae; phylum: Pteridophyta; class: Lycopodiopsida; order: Selaginellales; family: Selaginellaceae; genus: Selaginella; specificEpithet: versicolor; scientificNameAuthorship: Spring; **Location:** continent: Africa; country: Togo; countryCode: TG; locality: Assoukoko; verbatimElevation: 366; verbatimSRS: WGS84; decimalLatitude: 8.000007; decimalLongitude: 0.61621; geodeticDatum: WGS84; **Identification:** identifiedBy: Abotsi, K.E.; dateIdentified: 09-18-16; **Event:** eventDate: 09-18-16; habitat: Rainforest; **Record Level:** institutionID: Herbarium Togoense; collectionID: Abotsi, K.E.; institutionCode: TOGO; basisOfRecord: Preserved specimen
- **Type status:** Other material. **Occurrence:** recordNumber: AB0592; recordedBy: Abotsi, K.E.; **Taxon:** scientificName: Selaginella versicolor Spring; namePublishedIn: Bull. Acad. Roy. Soc. Bruxelles 10: 143, no. 57 (1843); kingdom: Plantae; phylum: Pteridophyta; class: Lycopodiopsida; order: Selaginellales; family: Selaginellaceae; genus: Selaginella; specificEpithet: versicolor; scientificNameAuthorship: Spring; **Location:** continent: Africa; country: Togo; countryCode: TG; locality: Fazao; verbatimElevation: 528; verbatimSRS: WGS84; decimalLatitude: 8.626591; decimalLongitude: 0.762663; geodeticDatum: WGS84; **Identification:** identifiedBy: Abotsi, K.E.; dateIdentified: 07-17-17; **Event:** eventDate: 07-17-17; habitat: Dry dense forest; **Record Level:** institutionID: Herbarium Togoense; collectionID: Abotsi, K.E.; institutionCode: TOGO; basisOfRecord: Preserved specimen

##### Ecological interactions

###### Native status

Native

##### Distribution

Zones 2 and 4

#### Selaginella vogelii

Spring

##### Materials

- **Type status:** Other material. **Occurrence:** recordNumber: AB0180; recordedBy: Abotsi, K.E.; **Taxon:** scientificName: Selaginella vogelii Spring; namePublishedIn: Monogr. Lyc. 2: 170, no. 111 (1850); kingdom: Plantae; phylum: Pteridophyta; class: Lycopodiopsida; order: Selaginellales; family: Selaginellaceae; genus: Selaginella; specificEpithet: vogelii; scientificNameAuthorship: Spring; **Location:** continent: Africa; country: Togo; countryCode: TG; locality: Womé; verbatimElevation: 322; verbatimSRS: WGS84; decimalLatitude: 6.85783; decimalLongitude: 0.554387; geodeticDatum: WGS84; **Identification:** identifiedBy: Abotsi, K.E.; dateIdentified: 08-03-16; **Event:** eventDate: 08-03-16; habitat: Rainforest; **Record Level:** institutionID: Herbarium Togoense; collectionID: Abotsi, K.E.; institutionCode: TOGO; basisOfRecord: Preserved specimen
- **Type status:** Other material. **Occurrence:** recordNumber: AB0262; recordedBy: Abotsi, K.E.; **Taxon:** scientificName: Selaginella vogelii Spring; namePublishedIn: Monogr. Lyc. 2: 170, no. 111 (1850); kingdom: Plantae; phylum: Pteridophyta; class: Lycopodiopsida; order: Selaginellales; family: Selaginellaceae; genus: Selaginella; specificEpithet: vogelii; scientificNameAuthorship: Spring; **Location:** continent: Africa; country: Togo; countryCode: TG; locality: Badou; verbatimElevation: 401; verbatimSRS: WGS84; decimalLatitude: 7.580799; decimalLongitude: 0.6227; geodeticDatum: WGS84; **Identification:** identifiedBy: Abotsi, K.E.; dateIdentified: 08-08-16; **Event:** eventDate: 08-08-16; habitat: Rainforest; **Record Level:** institutionID: Herbarium Togoense; collectionID: Abotsi, K.E.; institutionCode: TOGO; basisOfRecord: Preserved specimen
- **Type status:** Other material. **Occurrence:** recordNumber: AB0573; recordedBy: Abotsi, K.E.; **Taxon:** scientificName: Selaginella vogelii Spring; namePublishedIn: Monogr. Lyc. 2: 170, no. 111 (1850); kingdom: Plantae; phylum: Pteridophyta; class: Lycopodiopsida; order: Selaginellales; family: Selaginellaceae; genus: Selaginella; specificEpithet: vogelii; scientificNameAuthorship: Spring; **Location:** continent: Africa; country: Togo; countryCode: TG; locality: Souroukou; verbatimElevation: 239; verbatimSRS: WGS84; decimalLatitude: 8.755171; decimalLongitude: 0.684436; geodeticDatum: WGS84; **Identification:** identifiedBy: Abotsi, K.E.; dateIdentified: 07-15-17; **Event:** eventDate: 07-15-17; habitat: Dry dense forest; **Record Level:** institutionID: Herbarium Togoense; collectionID: Abotsi, K.E.; institutionCode: TOGO; basisOfRecord: Preserved specimen

##### Ecological interactions

###### Native status

Native

##### Distribution

Zones 2 and 4

#### Selaginella zechii

Hieron.

##### Materials

- **Type status:** Other material. **Occurrence:** catalogNumber: 12382; recordNumber: 4887; recordedBy: Brunel, J.-F.; **Taxon:** scientificName: Selaginella zechii Hieron.; namePublishedIn: Engl. & Prantl, Nat. Pfl. 1(4): 697, no. 298. (1901 publ. 1902); kingdom: Plantae; phylum: Pteridophyta; class: Lycopodiopsida; order: Selaginellales; family: Selaginellaceae; genus: Selaginella; specificEpithet: zechii; scientificNameAuthorship: Hieron.; **Location:** continent: Africa; country: Togo; countryCode: TG; locality: Kpimé Séva; verbatimElevation: 527; verbatimSRS: WGS84; decimalLatitude: 7.004519; decimalLongitude: 0.633291; geodeticDatum: WGS84; **Identification:** identifiedBy: Brunel, J.-F.; dateIdentified: /2/1974; **Event:** eventDate: /2/1974; habitat: Rainforest; **Record Level:** institutionID: Herbarium Togoense; collectionID: Brunel, J.-F.; institutionCode: TOGO; basisOfRecord: Preserved specimen

##### Ecological interactions

###### Native status

Native

##### Distribution

Zone 4

#### Polypodiopsida

#### Marattiidae

#### Marattiales

#### Marattiaceae

#### Ptisana salicifolia

(Schrad.) Senterre & Rouhan

##### Materials

- **Type status:** Other material. **Occurrence:** recordNumber: AB0353; recordedBy: Abotsi, K.E.; **Taxon:** scientificName: Ptisana salicifolia (Schrad.) Senterre & Rouhan; namePublishedIn: Phytotaxa 158(1): 70 (2014); kingdom: Plantae; phylum: Pteridophyta; class: Polypodiopsida; order: Marattiales; family: Marattiaceae; genus: Ptisana; specificEpithet: salicifolia; scientificNameAuthorship: (Schrad.) Senterre & Rouhan; **Location:** continent: Africa; country: Togo; countryCode: TG; locality: Danyi Gbaladzé; verbatimElevation: 798; verbatimSRS: WGS84; decimalLatitude: 7.207299; decimalLongitude: 0.731471; geodeticDatum: WGS84; **Identification:** identifiedBy: Abotsi, K.E.; dateIdentified: 42644; **Event:** eventDate: 10-01-16; habitat: Rainforest; **Record Level:** institutionID: Herbarium Togoense; collectionID: Abotsi, K.E.; institutionCode: TOGO; basisOfRecord: Preserved specimen
- **Type status:** Other material. **Occurrence:** recordNumber: AB0525; recordedBy: Abotsi, K.E.; **Taxon:** scientificName: Ptisana salicifolia (Schrad.) Senterre & Rouhan; namePublishedIn: Phytotaxa 158(1): 70 (2014); kingdom: Plantae; phylum: Pteridophyta; class: Polypodiopsida; order: Marattiales; family: Marattiaceae; genus: Ptisana; specificEpithet: salicifolia; scientificNameAuthorship: (Schrad.) Senterre & Rouhan; **Location:** continent: Africa; country: Togo; countryCode: TG; locality: Gobè; verbatimElevation: 720; verbatimSRS: WGS84; decimalLatitude: 7.501756; decimalLongitude: 0.799352; geodeticDatum: WGS84; **Identification:** identifiedBy: Abotsi, K.E.; dateIdentified: 04-26-17; **Event:** eventDate: 04-26-17; habitat: Rainforest; **Record Level:** institutionID: Herbarium Togoense; collectionID: Abotsi, K.E.; institutionCode: TOGO; basisOfRecord: Preserved specimen
- **Type status:** Other material. **Occurrence:** recordNumber: AB0511; recordedBy: Abotsi, K.E.; **Taxon:** scientificName: Ptisana salicifolia (Schrad.) Senterre & Rouhan; namePublishedIn: Phytotaxa 158(1): 70 (2014); kingdom: Plantae; phylum: Pteridophyta; class: Polypodiopsida; order: Marattiales; family: Marattiaceae; genus: Ptisana; specificEpithet: salicifolia; scientificNameAuthorship: (Schrad.) Senterre & Rouhan; **Location:** continent: Africa; country: Togo; countryCode: TG; locality: Gbadi Gawodo; verbatimElevation: 636; verbatimSRS: WGS84; decimalLatitude: 7.484688; decimalLongitude: 0.766313; geodeticDatum: WGS84; **Identification:** identifiedBy: Abotsi, K.E.; dateIdentified: 04-26-17; **Event:** eventDate: 04-26-17; habitat: Rainforest; **Record Level:** institutionID: Herbarium Togoense; collectionID: Abotsi, K.E.; institutionCode: TOGO; basisOfRecord: Preserved specimen

##### Ecological interactions

###### Native status

Native

##### Distribution

Zone 4

#### Ophioglossidae

#### Ophioglossales

#### Ophioglossaceae

#### Ophioglossum costatum

R.Br.

##### Materials

- **Type status:** Other material. **Occurrence:** recordNumber: AB0401; recordedBy: Abotsi, K.E.; **Taxon:** scientificName: Ophioglossum costatum R.Br.; namePublishedIn: Prodr. Fl. Nov. Holl. 163 (1810); kingdom: Plantae; phylum: Pteridophyta; class: Polypodiopsida; order: Ophioglossales; family: Ophioglossaceae; genus: Ophioglossum; specificEpithet: costatum; scientificNameAuthorship: R.Br.; **Location:** continent: Africa; country: Togo; countryCode: TG; locality: Rodokpé; verbatimElevation: 168; verbatimSRS: WGS84; decimalLatitude: 7.04094; decimalLongitude: 1.155844; geodeticDatum: WGS84; **Identification:** identifiedBy: Abotsi, K.E.; dateIdentified: 10-07-16; **Event:** eventDate: 10-07-16; habitat: Wooded savannah; **Record Level:** institutionID: Herbarium Togoense; collectionID: Abotsi, K.E.; institutionCode: TOGO; basisOfRecord: Preserved specimen
- **Type status:** Other material. **Occurrence:** recordNumber: AB0598; recordedBy: Abotsi, K.E.; **Taxon:** scientificName: Ophioglossum costatum R.Br.; namePublishedIn: Prodr. Fl. Nov. Holl. 163 (1810); kingdom: Plantae; phylum: Pteridophyta; class: Polypodiopsida; order: Ophioglossales; family: Ophioglossaceae; genus: Ophioglossum; specificEpithet: costatum; scientificNameAuthorship: R.Br.; **Location:** continent: Africa; country: Togo; countryCode: TG; locality: Fazao; verbatimElevation: 534; verbatimSRS: WGS84; decimalLatitude: 8.631144; decimalLongitude: 0.766771; geodeticDatum: WGS84; **Identification:** identifiedBy: Abotsi, K.E.; dateIdentified: 07-17-17; **Event:** eventDate: 07-17-17; habitat: Shrub savannah; **Record Level:** institutionID: Herbarium Togoense; collectionID: Abotsi, K.E.; institutionCode: TOGO; basisOfRecord: Preserved specimen
- **Type status:** Other material. **Occurrence:** recordNumber: AB0599; recordedBy: Abotsi, K.E.; **Taxon:** scientificName: Ophioglossum costatum R.Br.; namePublishedIn: Prodr. Fl. Nov. Holl. 163 (1810); kingdom: Plantae; phylum: Pteridophyta; class: Polypodiopsida; order: Ophioglossales; family: Ophioglossaceae; genus: Ophioglossum; specificEpithet: costatum; scientificNameAuthorship: R.Br.; **Location:** continent: Africa; country: Togo; countryCode: TG; locality: Fazao; verbatimElevation: 533; verbatimSRS: WGS84; decimalLatitude: 8.631118; decimalLongitude: 0.766724; geodeticDatum: WGS84; **Identification:** identifiedBy: Abotsi, K.E.; dateIdentified: 07-17-17; **Event:** eventDate: 07-17-17; habitat: Shrub savannah; **Record Level:** institutionID: Herbarium Togoense; collectionID: Abotsi, K.E.; institutionCode: TOGO; basisOfRecord: Preserved specimen

##### Ecological interactions

###### Native status

Native

##### Distribution

Zones 2 and 3

#### Ophioglossum gomezianum

Welw. ex A. Br. apud Kuhn

##### Materials

- **Type status:** Other material. **Occurrence:** catalogNumber: 12382; recordNumber: 2018; recordedBy: Akpagana, K.; **Taxon:** scientificName: Ophioglossum gomezianum Welw. ex A. Br. apud Kuhn; namePublishedIn: Fil. Afr. 176 (1868); kingdom: Plantae; phylum: Pteridophyta; class: Polypodiopsida; order: Ophioglossales; family: Ophioglossaceae; genus: Ophioglossum; specificEpithet: gomezianum; scientificNameAuthorship: Welw. ex A. Br. apud Kuhn; **Location:** continent: Africa; country: Togo; countryCode: TG; locality: Oké; verbatimElevation: 200; verbatimSRS: WGS84; decimalLatitude: 7.4333333; decimalLongitude: 1.4166667; geodeticDatum: WGS84; **Identification:** identifiedBy: K. Akpagana; dateIdentified: 07-01-89; **Event:** eventDate: 07-01-89; habitat: Wooded savannah; **Record Level:** institutionID: Herbarium Togoense; collectionID: Akpagana, K.; institutionCode: TOGO; basisOfRecord: Preserved specimen

##### Ecological interactions

###### Native status

Native

##### Distribution

Zone 3

#### Ophioglossum gramineum

Willd.

##### Materials

- **Type status:** Other material. **Occurrence:** catalogNumber: 12381; recordNumber: 2040; recordedBy: Akpagana, K.; **Taxon:** scientificName: Ophioglossum gramineum Willd.; namePublishedIn: Nov. Act. Acad. Erf. 2: 18, t. 1, f. 1 (1802); kingdom: Plantae; phylum: Pteridophyta; class: Polypodiopsida; order: Ophioglossales; family: Ophioglossaceae; genus: Ophioglossum; specificEpithet: gramineum; scientificNameAuthorship: Willd.; **Location:** continent: Africa; country: Togo; countryCode: TG; locality: Akodessewa; verbatimElevation: 180; verbatimSRS: WGS84; decimalLatitude: 7.4333333; decimalLongitude: 1.4166667; geodeticDatum: WGS84; **Identification:** identifiedBy: K. Akpagana; dateIdentified: 08-01-89; **Event:** eventDate: 08-01-89; habitat: Wooded savannah; **Record Level:** institutionID: Herbarium Togoense; collectionID: Akpagana, K.; institutionCode: TOGO; basisOfRecord: Preserved specimen

##### Ecological interactions

###### Native status

Native

##### Distribution

Zone 3

#### Ophioglossum reticulatum

L.

##### Materials

- **Type status:** Other material. **Occurrence:** catalogNumber: 12380; recordNumber: 2037 bis; recordedBy: Akpagana, K.; **Taxon:** scientificName: Ophioglossum reticulatum L.; namePublishedIn: Sp. Pl. 2: 1063 (1753); kingdom: Plantae; phylum: Pteridophyta; class: Polypodiopsida; order: Ophioglossales; family: Ophioglossaceae; genus: Ophioglossum; specificEpithet: reticulatum; scientificNameAuthorship: L.; **Location:** continent: Africa; country: Togo; countryCode: TG; locality: Hounto; verbatimElevation: 210; verbatimSRS: WGS84; decimalLatitude: 7.4333333; decimalLongitude: 1.4166667; geodeticDatum: WGS84; **Identification:** identifiedBy: K. Akpagana; dateIdentified: 06-01-89; **Event:** eventDate: 06-01-89; habitat: Wooded savannah; **Record Level:** institutionID: Herbarium Togoense; collectionID: Akpagana, K.; institutionCode: TOGO; basisOfRecord: Preserved specimen

##### Ecological interactions

###### Native status

Native

##### Distribution

Zone 3

#### Ophioglossum rubellum

A. Braun..

##### Materials

- **Type status:** Other material. **Occurrence:** catalogNumber: 12378; recordNumber: 2023 bis; recordedBy: Akpagana, K.; **Taxon:** scientificName: Ophioglossum rubellum A. Braun..; namePublishedIn: Kuhn, Fil. Afr. 179 (1868); kingdom: Plantae; phylum: Pteridophyta; class: Polypodiopsida; order: Ophioglossales; family: Ophioglossaceae; genus: Ophioglossum; specificEpithet: rubellum; scientificNameAuthorship: A. Braun..; **Location:** continent: Africa; country: Togo; countryCode: TG; locality: Akodessewa; verbatimElevation: 182; verbatimSRS: WGS84; decimalLatitude: 7.4333333; decimalLongitude: 1.4166667; geodeticDatum: WGS84; **Identification:** identifiedBy: K. Akpagana; dateIdentified: 07-01-89; **Event:** eventDate: 07-01-89; habitat: Wooded savannah; **Record Level:** institutionID: Herbarium Togoense; collectionID: Akpagana, K.; institutionCode: TOGO; basisOfRecord: Preserved specimen
- **Type status:** Other material. **Occurrence:** catalogNumber: 12379; recordNumber: 2024 bis; recordedBy: Akpagana, K.; **Taxon:** scientificName: Ophioglossum rubellum A. Braun..; namePublishedIn: Kuhn, Fil. Afr. 179 (1868); kingdom: Plantae; phylum: Pteridophyta; class: Polypodiopsida; order: Ophioglossales; family: Ophioglossaceae; genus: Ophioglossum; specificEpithet: rubellum; scientificNameAuthorship: A. Braun..; **Location:** continent: Africa; country: Togo; countryCode: TG; locality: Atchakoe; verbatimElevation: 160; verbatimSRS: WGS84; decimalLatitude: 7.4333333; decimalLongitude: 1.4166667; geodeticDatum: WGS84; **Identification:** identifiedBy: K. Akpagana; dateIdentified: 07-01-89; **Event:** eventDate: 07-01-89; habitat: Wooded savannah; **Record Level:** institutionID: Herbarium Togoense; collectionID: Akpagana, K.; institutionCode: TOGO; basisOfRecord: Preserved specimen

##### Ecological interactions

###### Native status

Native

##### Distribution

Zone 3

#### Polypodiidae

#### Cyatheales

#### Cyatheaceae

#### Alsophila camerooniana

(Hook.) R. M. Tryon

##### Materials

- **Type status:** Other material. **Occurrence:** recordNumber: AB0357; recordedBy: Abotsi, K.E.; **Taxon:** scientificName: Alsophila camerooniana (Hook.) R. M. Tryon; namePublishedIn: Contr. Gray Herb. 200: 30 (1970); kingdom: Plantae; phylum: Pteridophyta; class: Polypodiopsida; order: Cyatheales; family: Cyatheaceae; genus: Alsophila; specificEpithet: camerooniana; scientificNameAuthorship: (Hook.) R. M. Tryon; **Location:** continent: Africa; country: Togo; countryCode: TG; locality: Danyi Gbaladzé; verbatimElevation: 799; verbatimSRS: WGS84; decimalLatitude: 7.207184; decimalLongitude: 0.731346; geodeticDatum: WGS84; **Identification:** identifiedBy: Abotsi, K.E.; dateIdentified: 10-01-16; **Event:** eventDate: 10-01-16; habitat: Rainforest; **Record Level:** institutionID: Herbarium Togoense; collectionID: Abotsi, K.E.; institutionCode: TOGO; basisOfRecord: Preserved specimen
- **Type status:** Other material. **Occurrence:** recordNumber: AB0423; recordedBy: Abotsi, K.E.; **Taxon:** scientificName: Alsophila camerooniana (Hook.) R. M. Tryon; namePublishedIn: Contr. Gray Herb. 200: 30 (1970); kingdom: Plantae; phylum: Pteridophyta; class: Polypodiopsida; order: Cyatheales; family: Cyatheaceae; genus: Alsophila; specificEpithet: camerooniana; scientificNameAuthorship: (Hook.) R. M. Tryon; **Location:** continent: Africa; country: Togo; countryCode: TG; locality: Danyi Dzogbégan; verbatimElevation: 722; verbatimSRS: WGS84; decimalLatitude: 7.23171; decimalLongitude: 0.677705; geodeticDatum: WGS84; **Identification:** identifiedBy: Abotsi, K.E.; dateIdentified: 12-27-16; **Event:** eventDate: 12-27-16; habitat: Rainforest; **Record Level:** institutionID: Herbarium Togoense; collectionID: Abotsi, K.E.; institutionCode: TOGO; basisOfRecord: Preserved specimen
- **Type status:** Other material. **Occurrence:** recordNumber: AB0512; recordedBy: Abotsi, K.E.; **Taxon:** scientificName: Alsophila camerooniana (Hook.) R. M. Tryon; namePublishedIn: Contr. Gray Herb. 200: 30 (1970); kingdom: Plantae; phylum: Pteridophyta; class: Polypodiopsida; order: Cyatheales; family: Cyatheaceae; genus: Alsophila; specificEpithet: camerooniana; scientificNameAuthorship: (Hook.) R. M. Tryon; **Location:** continent: Africa; country: Togo; countryCode: TG; locality: Gbadi Gawodo; verbatimElevation: 636; verbatimSRS: WGS84; decimalLatitude: 7.484655; decimalLongitude: 0.766314; geodeticDatum: WGS84; **Identification:** identifiedBy: Abotsi, K.E.; dateIdentified: 04-26-17; **Event:** eventDate: 04-26-17; habitat: Rainforest; **Record Level:** institutionID: Herbarium Togoense; collectionID: Abotsi, K.E.; institutionCode: TOGO; basisOfRecord: Preserved specimen

##### Ecological interactions

###### Native status

Native

##### Distribution

Zone 4

#### Gleicheniales

#### Gleicheniaceae

#### Dicranopteris linearis

(Burm.f.) Underw.

##### Materials

- **Type status:** Other material. **Occurrence:** recordNumber: AB0613; recordedBy: Abotsi, K.E.; **Taxon:** scientificName: Dicranopteris linearis (Burm.f.) Underw.; namePublishedIn: Bull. Torrey Bot. Cl. 34: 249 (1907); kingdom: Plantae; phylum: Pteridophyta; class: Polypodiopsida; order: Gleicheniales; family: Gleicheniaceae; genus: Dicranopteris; specificEpithet: linearis; scientificNameAuthorship: (Burm.f.) Underw.; **Location:** continent: Africa; country: Togo; countryCode: TG; locality: Kouma Apoti; verbatimElevation: 684; verbatimSRS: WGS84; decimalLatitude: 6.980898; decimalLongitude: 0.562334; geodeticDatum: WGS84; **Identification:** identifiedBy: Abotsi, K.E.; dateIdentified: 01-25-18; **Event:** eventDate: 01-25-18; habitat: Rainforest; **Record Level:** institutionID: Herbarium Togoense; collectionID: Abotsi, K.E.; institutionCode: TOGO; basisOfRecord: Preserved specimen
- **Type status:** Other material. **Occurrence:** recordNumber: AB0614; recordedBy: Abotsi, K.E.; **Taxon:** scientificName: Dicranopteris linearis (Burm.f.) Underw.; namePublishedIn: Bull. Torrey Bot. Cl. 34: 249 (1907); kingdom: Plantae; phylum: Pteridophyta; class: Polypodiopsida; order: Gleicheniales; family: Gleicheniaceae; genus: Dicranopteris; specificEpithet: linearis; scientificNameAuthorship: (Burm.f.) Underw.; **Location:** continent: Africa; country: Togo; countryCode: TG; locality: Agomé Pédo; verbatimElevation: 712; verbatimSRS: WGS84; decimalLatitude: 6.934271; decimalLongitude: 0.570366; geodeticDatum: WGS84; **Identification:** identifiedBy: Abotsi, K.E.; dateIdentified: 01-26-18; **Event:** eventDate: 01-26-18; habitat: Rainforest; **Record Level:** institutionID: Herbarium Togoense; collectionID: Abotsi, K.E.; institutionCode: TOGO; basisOfRecord: Preserved specimen

##### Ecological interactions

###### Native status

Native

##### Distribution

Zone 4

#### Hymenophyllales

#### Hymenophyllaceae

#### Crepidomanes africanum

(Christ) Ebihara & Dubuisson

##### Materials

- **Type status:** Other material. **Occurrence:** recordNumber: AB0475; recordedBy: Abotsi, K.E.; **Taxon:** scientificName: Crepidomanes africanum (Christ) Ebihara & Dubuisson; namePublishedIn: Blumea 51(2): 238 (2006); kingdom: Plantae; phylum: Pteridophyta; class: Polypodiopsida; order: Hymenophyllales; family: Hymenophyllaceae; genus: Crepidomanes; specificEpithet: africanum; scientificNameAuthorship: (Christ) Ebihara & Dubuisson; **Location:** continent: Africa; country: Togo; countryCode: TG; locality: Danyi Dzogbégan; verbatimElevation: 715; verbatimSRS: WGS84; decimalLatitude: 7.232032; decimalLongitude: 0.677687; geodeticDatum: WGS84; **Identification:** identifiedBy: Abotsi, K.E.; dateIdentified: 04-24-17; **Event:** eventDate: 04-24-17; habitat: Rainforest; **Record Level:** institutionID: Herbarium Togoense; collectionID: Abotsi, K.E.; institutionCode: TOGO; basisOfRecord: Preserved specimen

##### Ecological interactions

###### Native status

Native

##### Distribution

Zone 4

#### Crepidomanes minutum

(Blume) K. Iwats

##### Materials

- **Type status:** Other material. **Occurrence:** recordedBy: Roux, J.P.; **Taxon:** scientificName: Crepidomanes minutum (Blume) K. Iwats.; namePublishedIn: J. Fac. Sci. Univ. Tokyo, Sect. 3, Bot. 13(5): 524 (1985); kingdom: Plantae; phylum: Pteridophyta; class: Polypodiopsida; order: Hymenophyllales; family: Hymenophyllaceae; genus: Crepidomanes; specificEpithet: minutum; scientificNameAuthorship: (Blume) K. Iwats.; **Location:** continent: Africa; country: Togo; countryCode: TG; **Event:** habitat: Rainforest; **Record Level:** basisOfRecord: Unknown; source: Synopsis of the Lycopodiophyta and Pteridophyta of Africa, Madagascar and neighbouring islands (Roux, 2009)

##### Ecological interactions

###### Native status

Native

##### Distribution

Zone 4

#### Didymoglossum chamaedrys

(Taton) J. P. Roux

##### Materials

- **Type status:** Other material. **Occurrence:** catalogNumber: 12215; recordNumber: 8142 bis; recordedBy: Brunel, J.-F.; **Taxon:** scientificName: Didymoglossum chamaedrys (Taton) J. P. Roux; namePublishedIn: Strelitzia 23: 41 (2009); kingdom: Plantae; phylum: Pteridophyta; class: Polypodiopsida; order: Hymenophyllales; family: Hymenophyllaceae; genus: Didymoglossum; specificEpithet: chamaedrys; scientificNameAuthorship: (Taton) J. P. Roux; **Location:** continent: Africa; country: Togo; countryCode: TG; locality: Akloa; verbatimElevation: 335; verbatimSRS: WGS84; decimalLatitude: 7.5833333; decimalLongitude: 0.6; geodeticDatum: WGS84; **Identification:** identifiedBy: Brunel, J.-F.; dateIdentified: 01-01-84; **Event:** eventDate: 01-01-84; habitat: Rainforest; **Record Level:** institutionID: Herbarium Togoense; collectionID: Brunel, J.-F.; institutionCode: TOGO; basisOfRecord: Preserved specimen

##### Ecological interactions

###### Native status

Native

##### Distribution

Zone 4

#### Didymoglossum erosum

(Willd.) Beentje

##### Materials

- **Type status:** Other material. **Occurrence:** recordedBy: Roux, J.P.; **Taxon:** scientificName: Didymoglossum erosum (Willd.) Beentje; namePublishedIn: Fl. Trop. East Africa, Hymenophyllaceae: 13 (2008); kingdom: Plantae; phylum: Pteridophyta; class: Polypodiopsida; order: Hymenophyllales; family: Hymenophyllaceae; genus: Didymoglossum; specificEpithet: erosum; scientificNameAuthorship: (Willd.) Beentje; **Location:** continent: Africa; country: Togo; countryCode: TG; **Event:** habitat: Rainforest; **Record Level:** basisOfRecord: Unknown; source: Synopsis of the Lycopodiophyta and Pteridophyta of Africa, Madagascar and neighbouring islands (Roux, 2009)

##### Ecological interactions

###### Native status

Native

##### Distribution

Zone 4

#### Osmundales

#### Osmundaceae

#### Osmunda hilsenbergii

Hook. & Grev.

##### Materials

- **Type status:** Other material. **Occurrence:** recordNumber: AB0425; recordedBy: Abotsi, K.E.; **Taxon:** scientificName: Osmunda hilsenbergii Hook. & Grev.; namePublishedIn: J. Bot. (Hook.) Kew Misc. 3: 230 (1833); kingdom: Plantae; phylum: Pteridophyta; class: Polypodiopsida; order: Osmundales; family: Osmundaceae; genus: Osmunda; specificEpithet: hilsenbergii; scientificNameAuthorship: Hook. & Grev.; **Location:** continent: Africa; country: Togo; countryCode: TG; locality: Danyi Dzogbégan; verbatimElevation: 720; verbatimSRS: WGS84; decimalLatitude: 7.231747; decimalLongitude: 0.677667; geodeticDatum: WGS84; **Identification:** identifiedBy: Abotsi, K.E.; dateIdentified: 12-27-16; **Event:** eventDate: 12-27-16; habitat: Rainforest; **Record Level:** institutionID: Herbarium Togoense; collectionID: Abotsi, K.E.; institutionCode: TOGO; basisOfRecord: Preserved specimen

##### Ecological interactions

###### Native status

Native

##### Distribution

Zone 4

#### Polypodiales

#### Aspleniineae

#### Aspleniaceae

#### Asplenium aethiopicum

(Burm.f.) Becherer

##### Materials

- **Type status:** Other material. **Occurrence:** recordNumber: AB0071; recordedBy: Abotsi, K.E.; **Taxon:** scientificName: Asplenium aethiopicum (Burm. f.) Becherer; namePublishedIn: Candollea 6: 23 (1935); kingdom: Plantae; phylum: Pteridophyta; class: Polypodiopsida; order: Polypodiales; family: Aspleniaceae; genus: Asplenium; specificEpithet: aethiopicum; scientificNameAuthorship: (Burm. f.) Becherer; **Location:** continent: Africa; country: Togo; countryCode: TG; locality: Kuma Konda; verbatimElevation: 665; verbatimSRS: WGS84; decimalLatitude: 6.954179; decimalLongitude: 0.584052; geodeticDatum: WGS84; **Identification:** identifiedBy: Abotsi, K.E.; dateIdentified: 06-24-16; **Event:** eventDate: 06-24-16; habitat: Rainforest; **Record Level:** institutionID: Herbarium Togoense; collectionID: Abotsi, K.E.; institutionCode: TOGO; basisOfRecord: Preserved specimen
- **Type status:** Other material. **Occurrence:** recordNumber: AB0061; recordedBy: Abotsi, K.E.; **Taxon:** scientificName: Asplenium aethiopicum var. aethiopicum (Burm. f.) Becherer; namePublishedIn: Candollea 6: 23 (1935); kingdom: Plantae; phylum: Pteridophyta; class: Polypodiopsida; order: Polypodiales; family: Aspleniaceae; genus: Asplenium; specificEpithet: aethiopicum; scientificNameAuthorship: (Burm. f.) Becherer; **Location:** continent: Africa; country: Togo; countryCode: TG; locality: Agomé Yoh; verbatimElevation: 381; verbatimSRS: WGS84; decimalLatitude: 6.951669; decimalLongitude: 0.597757; geodeticDatum: WGS84; **Identification:** identifiedBy: Abotsi, K.E.; dateIdentified: 06-15-16; **Event:** eventDate: 06-15-16; habitat: Rainforest; **Record Level:** institutionID: Herbarium Togoense; collectionID: Abotsi, K.E.; institutionCode: TOGO; basisOfRecord: Preserved specimen

##### Ecological interactions

###### Native status

Native

##### Distribution

Zone 4

#### Asplenium barteri

Hook.

##### Materials

- **Type status:** Other material. **Occurrence:** catalogNumber: 12186; recordNumber: 5751; recordedBy: Brunel, J.-F.; **Taxon:** scientificName: Asplenium barteri Hook.; namePublishedIn: A 2nd Cent. Ferns, t. 75 (1861); kingdom: Plantae; phylum: Pteridophyta; class: Polypodiopsida; order: Polypodiales; family: Aspleniaceae; genus: Asplenium; specificEpithet: barteri; scientificNameAuthorship: Hook.; **Location:** continent: Africa; country: Togo; countryCode: TG; locality: Kouma; verbatimElevation: 670; verbatimSRS: WGS84; decimalLatitude: 6.954128; decimalLongitude: 0.584035; geodeticDatum: WGS84; **Identification:** identifiedBy: Brunel, J.-F.; dateIdentified: /12/1978; **Event:** eventDate: /12/1978; habitat: Rainforest; **Record Level:** institutionID: Herbarium Togoense; collectionID: Brunel, J.-F.; institutionCode: TOGO; basisOfRecord: Preserved specimen

##### Ecological interactions

###### Native status

Native

##### Distribution

Zone 4

#### Asplenium biafranum

Alston & Ballard ex Ballard

##### Materials

- **Type status:** Other material. **Occurrence:** recordNumber: AB0137; recordedBy: Abotsi, K.E.; **Taxon:** scientificName: Asplenium biafranum Alston & Ballard ex Ballard; namePublishedIn: Hook., Ic. Pl. ser.5. 4: t. 3367 (1938); kingdom: Plantae; phylum: Pteridophyta; class: Polypodiopsida; order: Polypodiales; family: Aspleniaceae; genus: Asplenium; specificEpithet: biafranum; scientificNameAuthorship: Alston & Ballard ex Ballard; **Location:** continent: Africa; country: Togo; countryCode: TG; locality: Danyi N'Digbé, near Danyi Amégapé; verbatimElevation: 595; verbatimSRS: WGS84; decimalLatitude: 7.11577; decimalLongitude: 0.661522; geodeticDatum: WGS84; **Identification:** identifiedBy: Abotsi, K.E.; dateIdentified: 06-28-16; **Event:** eventDate: 06-28-16; habitat: Rainforest; **Record Level:** institutionID: Herbarium Togoense; collectionID: Abotsi, K.E.; institutionCode: TOGO; basisOfRecord: Preserved specimen
- **Type status:** Other material. **Occurrence:** catalogNumber: 12170; recordNumber: 5499; recordedBy: Brunel, J.-F.; **Taxon:** scientificName: Asplenium biafranum Alston & Ballard ex Ballard; namePublishedIn: Hook., Ic. Pl. ser.5. 4: t. 3367 (1938); kingdom: Plantae; phylum: Pteridophyta; class: Polypodiopsida; order: Polypodiales; family: Aspleniaceae; genus: Asplenium; specificEpithet: biafranum; scientificNameAuthorship: Alston & Ballard ex Ballard; **Location:** continent: Africa; country: Togo; countryCode: TG; locality: Danyi Elavagnon; verbatimElevation: 778; verbatimSRS: WGS84; decimalLatitude: 7.2947; decimalLongitude: 0.7165; geodeticDatum: WGS84; **Identification:** identifiedBy: Brunel, J.-F.; dateIdentified: /10/1978; **Event:** eventDate: /10/1978; habitat: Rainforest; **Record Level:** institutionID: Herbarium Togoense; collectionID: Brunel, J.-F.; institutionCode: TOGO; basisOfRecord: Preserved specimen

##### Ecological interactions

###### Native status

Native

##### Distribution

Zone 4

#### Asplenium blastophorum

Hieron.

##### Materials

- **Type status:** Other material. **Occurrence:** recordedBy: Roux, J.P.; **Taxon:** scientificName: Asplenium blastophorum Hieron.; namePublishedIn: Engl. Jahrb. 46: 378 (1911); kingdom: Plantae; phylum: Pteridophyta; class: Polypodiopsida; order: Polypodiales; family: Aspleniaceae; genus: Asplenium; specificEpithet: blastophorum; scientificNameAuthorship: Hieron.; **Location:** continent: Africa; country: Togo; countryCode: TG; **Event:** habitat: Rainforest; **Record Level:** basisOfRecord: Unknown; source: Synopsis of the Lycopodiophyta and Pteridophyta of Africa, Madagascar and neighbouring islands (Roux, 2009)

##### Ecological interactions

###### Native status

Native

##### Distribution

Zone 4

#### Asplenium buettneri

Hieron. ex Brause

##### Materials

- **Type status:** Other material. **Occurrence:** recordNumber: AB0139; recordedBy: Abotsi, K.E.; **Taxon:** scientificName: Asplenium buettneri Sem.; namePublishedIn: Deutsche Zentralafr. Exp. 2: 23 (1910); kingdom: Plantae; phylum: Pteridophyta; class: Polypodiopsida; order: Polypodiales; family: Aspleniaceae; genus: Asplenium; specificEpithet: buettneri; scientificNameAuthorship: Hieron. ex Brause; **Location:** continent: Africa; country: Togo; countryCode: TG; locality: Danyi N'Digbé; verbatimElevation: 704; verbatimSRS: WGS85; decimalLatitude: 7.114806; decimalLongitude: 0.657008; geodeticDatum: WGS84; **Identification:** identifiedBy: Abotsi, K.E.; dateIdentified: 06-28-16; **Event:** eventDate: 06-28-16; habitat: Rainforest; **Record Level:** institutionID: Herbarium Togoense; collectionID: Abotsi, K.E.; institutionCode: TOGO; basisOfRecord: Preserved specimen
- **Type status:** Other material. **Occurrence:** recordNumber: AB0486; recordedBy: Abotsi, K.E.; **Taxon:** scientificName: Asplenium buettneri Sem.; namePublishedIn: Deutsche Zentralafr. Exp. 2: 23 (1910); kingdom: Plantae; phylum: Pteridophyta; class: Polypodiopsida; order: Polypodiales; family: Aspleniaceae; genus: Asplenium; specificEpithet: buettneri; scientificNameAuthorship: Hieron. ex Brause; **Location:** continent: Africa; country: Togo; countryCode: TG; locality: Ediwlou; verbatimElevation: 614; verbatimSRS: WGS86; decimalLatitude: 7.409661; decimalLongitude: 0.681092; geodeticDatum: WGS84; **Identification:** identifiedBy: Abotsi, K.E.; dateIdentified: 04-25-17; **Event:** eventDate: 04-25-17; habitat: Rainforest; **Record Level:** institutionID: Herbarium Togoense; collectionID: Abotsi, K.E.; institutionCode: TOGO; basisOfRecord: Preserved specimen
- **Type status:** Other material. **Occurrence:** recordNumber: AB0115; recordedBy: Abotsi, K.E.; **Taxon:** scientificName: Asplenium buettneri Sem.; namePublishedIn: Deutsche Zentralafr. Exp. 2: 23 (1910); kingdom: Plantae; phylum: Pteridophyta; class: Polypodiopsida; order: Polypodiales; family: Aspleniaceae; genus: Asplenium; specificEpithet: buettneri; scientificNameAuthorship: Hieron. ex Brause; **Location:** continent: Africa; country: Togo; countryCode: TG; locality: Danyi N'Digbé; verbatimElevation: 681; verbatimSRS: WGS84; decimalLatitude: 7.153141; decimalLongitude: 0.666893; geodeticDatum: WGS84; **Identification:** identifiedBy: Abotsi, K.E.; dateIdentified: 06-27-16; **Event:** eventDate: 06-27-16; habitat: Rainforest; **Record Level:** institutionID: Herbarium Togoense; collectionID: Abotsi, K.E.; institutionCode: TOGO; basisOfRecord: Preserved specimen

##### Ecological interactions

###### Native status

Native

##### Distribution

Zone 4

#### Asplenium bulbiferum

G.Forst.

##### Materials

- **Type status:** Other material. **Occurrence:** recordedBy: Abotsi, K.E.; **Taxon:** scientificName: Asplenium bulbiferum G.Forst.; namePublishedIn: Prod. 80 (1786); kingdom: Plantae; phylum: Pteridophyta; class: Polypodiopsida; order: Polypodiales; family: Aspleniaceae; genus: Asplenium; specificEpithet: bulbiferum; scientificNameAuthorship: G.Forst.; **Location:** continent: Africa; country: Togo; countryCode: TG; locality: Lomé, in gardens; **Identification:** identifiedBy: Abotsi, K.E.; **Event:** habitat: Gardens; **Record Level:** basisOfRecord: Human observation

##### Ecological interactions

###### Native status

Not native

##### Distribution

In gardens

#### Asplenium currorii

Hook.

##### Materials

- **Type status:** Other material. **Occurrence:** recordNumber: AB0121; recordedBy: Abotsi, K.E.; **Taxon:** scientificName: Asplenium currorii Hook.; namePublishedIn: Sp. 3: 82 (1860); kingdom: Plantae; phylum: Pteridophyta; class: Polypodiopsida; order: Polypodiales; family: Aspleniaceae; genus: Asplenium; specificEpithet: currorii; scientificNameAuthorship: Hook.; **Location:** continent: Africa; country: Togo; countryCode: TG; locality: Danyi N'Digbé; verbatimElevation: 667; verbatimSRS: WGS84; decimalLatitude: 7.153288; decimalLongitude: 0.666538; geodeticDatum: WGS84; **Identification:** identifiedBy: Abotsi, K.E.; dateIdentified: 06-27-16; **Event:** eventDate: 06-27-16; habitat: Rainforest; **Record Level:** institutionID: Herbarium Togoense; collectionID: Abotsi, K.E.; institutionCode: TOGO; basisOfRecord: Preserved specimen
- **Type status:** Other material. **Occurrence:** recordNumber: AB0390; recordedBy: Abotsi, K.E.; **Taxon:** scientificName: Asplenium currorii Hook.; namePublishedIn: Sp. 3: 82 (1860); kingdom: Plantae; phylum: Pteridophyta; class: Polypodiopsida; order: Polypodiales; family: Aspleniaceae; genus: Asplenium; specificEpithet: currorii; scientificNameAuthorship: Hook.; **Location:** continent: Africa; country: Togo; countryCode: TG; locality: Diguengué; verbatimElevation: 428; verbatimSRS: WGS84; decimalLatitude: 8.071387; decimalLongitude: 0.641095; geodeticDatum: WGS84; **Identification:** identifiedBy: Abotsi, K.E.; dateIdentified: 10-06-16; **Event:** eventDate: 10-06-16; habitat: Rainforest; **Record Level:** institutionID: Herbarium Togoense; collectionID: Abotsi, K.E.; institutionCode: TOGO; basisOfRecord: Preserved specimen
- **Type status:** Other material. **Occurrence:** recordNumber: AB0414; recordedBy: Abotsi, K.E.; **Taxon:** scientificName: Asplenium currorii Hook.; namePublishedIn: Sp. 3: 82 (1860); kingdom: Plantae; phylum: Pteridophyta; class: Polypodiopsida; order: Polypodiales; family: Aspleniaceae; genus: Asplenium; specificEpithet: currorii; scientificNameAuthorship: Hook.; **Location:** continent: Africa; country: Togo; countryCode: TG; locality: Danyi Dzogbégan; verbatimElevation: 713; verbatimSRS: WGS84; decimalLatitude: 7.232297; decimalLongitude: 0.677372; geodeticDatum: WGS84; **Identification:** identifiedBy: Abotsi, K.E.; dateIdentified: 12-27-16; **Event:** eventDate: 12-27-16; habitat: Rainforest; **Record Level:** institutionID: Herbarium Togoense; collectionID: Abotsi, K.E.; institutionCode: TOGO; basisOfRecord: Preserved specimen

##### Ecological interactions

###### Native status

Native

##### Distribution

Zone 4

#### Asplenium daucifolium

Lam.

##### Materials

- **Type status:** Other material. **Occurrence:** recordedBy: Abotsi, K.E.; **Taxon:** scientificName: Asplenium daucifolium Lam.; namePublishedIn: Encycl. 2: 310 (1786); kingdom: Plantae; phylum: Pteridophyta; class: Polypodiopsida; order: Polypodiales; family: Aspleniaceae; genus: Asplenium; specificEpithet: daucifolium; scientificNameAuthorship: Lam.; **Location:** continent: Africa; country: Togo; countryCode: TG; locality: Lomé, in gardens; **Identification:** identifiedBy: Abotsi, K.E.; **Event:** habitat: Gardens; **Record Level:** basisOfRecord: Human observation

##### Ecological interactions

###### Native status

Not native

##### Distribution

In gardens

#### Asplenium diplaziosorum

Hieron.

##### Materials

- **Type status:** Other material. **Occurrence:** catalogNumber: 12183; recordNumber: 1529; recordedBy: Akpagana, K.; **Taxon:** scientificName: Asplenium diplaziosorum Hieron.; namePublishedIn: Engl. Jahrb. 46: 351 (1911) (diplazisorum); kingdom: Plantae; phylum: Pteridophyta; class: Polypodiopsida; order: Polypodiales; family: Aspleniaceae; genus: Asplenium; specificEpithet: diplaziosorum; scientificNameAuthorship: Hieron.; **Location:** continent: Africa; country: Togo; countryCode: TG; locality: Danyi N'Digbé, Bavé area; verbatimElevation: 600; verbatimSRS: WGS84; decimalLatitude: 7.122676; decimalLongitude: 0.651209; geodeticDatum: WGS84; **Identification:** identifiedBy: K. Akpagana; dateIdentified: /03/1987; **Event:** eventDate: /03/1987; habitat: Rainforest; **Record Level:** institutionID: Herbarium Togoense; collectionID: Akpagana, K.; institutionCode: TOGO; basisOfRecord: Preserved specimen
- **Type status:** Other material. **Occurrence:** catalogNumber: 12184; recordNumber: 1502; recordedBy: Akpagana, K.; **Taxon:** scientificName: Asplenium diplaziosorum Hieron.; namePublishedIn: Engl. Jahrb. 46: 351 (1911) (diplazisorum); kingdom: Plantae; phylum: Pteridophyta; class: Polypodiopsida; order: Polypodiales; family: Aspleniaceae; genus: Asplenium; specificEpithet: diplaziosorum; scientificNameAuthorship: Hieron.; **Location:** continent: Africa; country: Togo; countryCode: TG; locality: Danyi N'Digbé, river Fufu; verbatimElevation: 717; verbatimSRS: WGS84; decimalLatitude: 7.146703; decimalLongitude: 0.675793; geodeticDatum: WGS84; **Identification:** identifiedBy: K. Akpagana; dateIdentified: /03/1987; **Event:** eventDate: /03/1987; habitat: Rainforest; **Record Level:** institutionID: Herbarium Togoense; collectionID: Akpagana, K.; institutionCode: TOGO; basisOfRecord: Preserved specimen

##### Ecological interactions

###### Native status

Native

##### Distribution

Zone 4

#### Asplenium dregeanum

Kunze

##### Materials

- **Type status:** Other material. **Occurrence:** recordNumber: AB0408; recordedBy: Abotsi, K.E.; **Taxon:** scientificName: Asplenium dregeanum Kunze; namePublishedIn: Linn. 10: 517 (1836); kingdom: Plantae; phylum: Pteridophyta; class: Polypodiopsida; order: Polypodiales; family: Aspleniaceae; genus: Asplenium; specificEpithet: dregeanum; scientificNameAuthorship: Kunze; **Location:** continent: Africa; country: Togo; countryCode: TG; locality: Mt Agou, Kébo-Dzigbé; verbatimElevation: 928; verbatimSRS: WGS84; decimalLatitude: 6.870528; decimalLongitude: 0.748695; geodeticDatum: WGS84; **Identification:** identifiedBy: Abotsi, K.E.; dateIdentified: 12-26-16; **Event:** eventDate: 12-26-16; habitat: Rainforest; **Record Level:** institutionID: Herbarium Togoense; collectionID: Abotsi, K.E.; institutionCode: TOGO; basisOfRecord: Preserved specimen
- **Type status:** Other material. **Occurrence:** recordNumber: ASM 0320; recordedBy: Abotsi, K.E., Sodjinou E. & Mingou P.; **Taxon:** scientificName: Asplenium dregeanum Kunze; namePublishedIn: Linn. 10: 517 (1836); kingdom: Plantae; phylum: Pteridophyta; class: Polypodiopsida; order: Polypodiales; family: Aspleniaceae; genus: Asplenium; specificEpithet: dregeanum; scientificNameAuthorship: Kunze; **Location:** continent: Africa; country: Togo; countryCode: TG; locality: Mt Agou, Kébo-Dzigbé; verbatimElevation: 868; verbatimSRS: WGS84; decimalLatitude: 6.869505505; decimalLongitude: 0.748776409; geodeticDatum: WGS84; **Identification:** identifiedBy: Abotsi, K.E.; dateIdentified: 04-16-13; **Event:** eventDate: 04-16-13; habitat: Rainforest; **Record Level:** institutionID: Herbarium Togoense; collectionID: Abotsi, K.E.; institutionCode: TOGO; basisOfRecord: Preserved specimen
- **Type status:** Other material. **Occurrence:** recordNumber: ASM 0322; recordedBy: Abotsi, K.E., Sodjinou E. & Mingou P.; **Taxon:** scientificName: Asplenium dregeanum Kunze; namePublishedIn: Linn. 10: 517 (1836); kingdom: Plantae; phylum: Pteridophyta; class: Polypodiopsida; order: Polypodiales; family: Aspleniaceae; genus: Asplenium; specificEpithet: dregeanum; scientificNameAuthorship: Kunze; **Location:** continent: Africa; country: Togo; countryCode: TG; locality: Mt Agou, Kébo-Dzigbé; verbatimElevation: 879; verbatimSRS: WGS84; decimalLatitude: 6.870573617; decimalLongitude: 0.747170511; geodeticDatum: WGS84; **Identification:** identifiedBy: Abotsi, K.E.; dateIdentified: 04-16-13; **Event:** eventDate: 04-16-13; habitat: Rainforest; **Record Level:** institutionID: Herbarium Togoense; collectionID: Abotsi, K.E.; institutionCode: TOGO; basisOfRecord: Preserved specimen

##### Ecological interactions

###### Native status

Native

##### Distribution

Zone 4

#### Asplenium emarginatum

P.Beauv.

##### Materials

- **Type status:** Other material. **Occurrence:** recordNumber: AB0142; recordedBy: Abotsi, K.E.; **Taxon:** scientificName: Asplenium emarginatum P.Beauv.; namePublishedIn: Flore d'Oware et de Bénin 2,11: 6, pl. 61 (18 Apr. 1808); kingdom: Plantae; phylum: Pteridophyta; class: Polypodiopsida; order: Polypodiales; family: Aspleniaceae; genus: Asplenium; specificEpithet: emarginatum; scientificNameAuthorship: P.Beauv.; **Location:** continent: Africa; country: Togo; countryCode: TG; locality: Danyi N'Digbé; verbatimElevation: 521; verbatimSRS: WGS84; decimalLatitude: 7.123263; decimalLongitude: 0.652707; geodeticDatum: WGS84; **Identification:** identifiedBy: Abotsi, K.E.; dateIdentified: 06-28-16; **Event:** eventDate: 06-28-16; habitat: Rainforest; **Record Level:** institutionID: Herbarium Togoense; collectionID: Abotsi, K.E.; institutionCode: TOGO; basisOfRecord: Preserved specimen
- **Type status:** Other material. **Occurrence:** recordNumber: AB0503; recordedBy: Abotsi, K.E.; **Taxon:** scientificName: Asplenium emarginatum P.Beauv.; namePublishedIn: Flore d'Oware et de Bénin 2,11: 6, pl. 61 (18 Apr. 1808); kingdom: Plantae; phylum: Pteridophyta; class: Polypodiopsida; order: Polypodiales; family: Aspleniaceae; genus: Asplenium; specificEpithet: emarginatum; scientificNameAuthorship: P.Beauv.; **Location:** continent: Africa; country: Togo; countryCode: TG; locality: Gbadi N'Kugna; verbatimElevation: 679; verbatimSRS: WGS84; decimalLatitude: 7.4541; decimalLongitude: 0.70565; geodeticDatum: WGS84; **Identification:** identifiedBy: Abotsi, K.E.; dateIdentified: 04-25-17; **Event:** eventDate: 04-25-17; habitat: Rainforest; **Record Level:** institutionID: Herbarium Togoense; collectionID: Abotsi, K.E.; institutionCode: TOGO; basisOfRecord: Preserved specimen
- **Type status:** Other material. **Occurrence:** recordNumber: AB0509; recordedBy: Abotsi, K.E.; **Taxon:** scientificName: Asplenium emarginatum P.Beauv.; namePublishedIn: Flore d'Oware et de Bénin 2,11: 6, pl. 61 (18 Apr. 1808); kingdom: Plantae; phylum: Pteridophyta; class: Polypodiopsida; order: Polypodiales; family: Aspleniaceae; genus: Asplenium; specificEpithet: emarginatum; scientificNameAuthorship: P.Beauv.; **Location:** continent: Africa; country: Togo; countryCode: TG; locality: Gbadi Gawodo; verbatimElevation: 638; verbatimSRS: WGS84; decimalLatitude: 7.484707; decimalLongitude: 0.766359; geodeticDatum: WGS84; **Identification:** identifiedBy: Abotsi, K.E.; dateIdentified: 04-26-17; **Event:** eventDate: 04-26-17; habitat: Rainforest; **Record Level:** institutionID: Herbarium Togoense; collectionID: Abotsi, K.E.; institutionCode: TOGO; basisOfRecord: Preserved specimen

##### Ecological interactions

###### Native status

Native

##### Distribution

Zone 4

#### Asplenium formosum

Willd.

##### Materials

- **Type status:** Other material. **Occurrence:** recordNumber: AB0038; recordedBy: Abotsi, K.E.; **Taxon:** scientificName: Asplenium formosum Willd.; namePublishedIn: Linn. Sp. Pl. ed. 4, 5: 329 (1810); kingdom: Plantae; phylum: Pteridophyta; class: Polypodiopsida; order: Polypodiales; family: Aspleniaceae; genus: Asplenium; specificEpithet: formosum; scientificNameAuthorship: Willd.; **Location:** continent: Africa; country: Togo; countryCode: TG; locality: Agou Kébo-Dzigbé; verbatimElevation: 765; verbatimSRS: WGS84; decimalLatitude: 6.863409; decimalLongitude: 0.75644; geodeticDatum: WGS84; **Identification:** identifiedBy: Abotsi, K.E.; dateIdentified: 06-14-16; **Event:** eventDate: 06-14-16; habitat: Rainforest; **Record Level:** institutionID: Herbarium Togoense; collectionID: Abotsi, K.E.; institutionCode: TOGO; basisOfRecord: Preserved specimen
- **Type status:** Other material. **Occurrence:** recordNumber: AB0051; recordedBy: Abotsi, K.E.; **Taxon:** scientificName: Asplenium formosum Willd.; namePublishedIn: Linn. Sp. Pl. ed. 4, 5: 329 (1810); kingdom: Plantae; phylum: Pteridophyta; class: Polypodiopsida; order: Polypodiales; family: Aspleniaceae; genus: Asplenium; specificEpithet: formosum; scientificNameAuthorship: Willd.; **Location:** continent: Africa; country: Togo; countryCode: TG; locality: Agomé Yoh; verbatimElevation: 463; verbatimSRS: WGS84; decimalLatitude: 6.952098; decimalLongitude: 0.590288; geodeticDatum: WGS84; **Identification:** identifiedBy: Abotsi, K.E.; dateIdentified: 06-15-16; **Event:** eventDate: 06-15-16; habitat: Rainforest; **Record Level:** institutionID: Herbarium Togoense; collectionID: Abotsi, K.E.; institutionCode: TOGO; basisOfRecord: Preserved specimen
- **Type status:** Other material. **Occurrence:** recordNumber: AB0072; recordedBy: Abotsi, K.E.; **Taxon:** scientificName: Asplenium formosum Willd.; namePublishedIn: Linn. Sp. Pl. ed. 4, 5: 329 (1810); kingdom: Plantae; phylum: Pteridophyta; class: Polypodiopsida; order: Polypodiales; family: Aspleniaceae; genus: Asplenium; specificEpithet: formosum; scientificNameAuthorship: Willd.; **Location:** continent: Africa; country: Togo; countryCode: TG; locality: Kuma Konda; verbatimElevation: 661; verbatimSRS: WGS84; decimalLatitude: 6.954178; decimalLongitude: 0.584035; geodeticDatum: WGS84; **Identification:** identifiedBy: Abotsi, K.E.; dateIdentified: 06-24-16; **Event:** eventDate: 06-24-16; habitat: Rainforest; **Record Level:** institutionID: Herbarium Togoense; collectionID: Abotsi, K.E.; institutionCode: TOGO; basisOfRecord: Preserved specimen

##### Ecological interactions

###### Native status

Native

##### Distribution

Zones 2 and 4

#### Asplenium hemitomum

Hieron.

##### Materials

- **Type status:** Other material. **Occurrence:** catalogNumber: 12213; recordNumber: 1788; recordedBy: Akpagana, K.; **Taxon:** scientificName: Asplenium hemitomum Hieron.; namePublishedIn: Engl. Jahrb. 46: 365 (1911); kingdom: Plantae; phylum: Pteridophyta; class: Polypodiopsida; order: Polypodiales; family: Aspleniaceae; genus: Asplenium; specificEpithet: hemitomum; scientificNameAuthorship: Hieron.; **Location:** continent: Africa; country: Togo; countryCode: TG; locality: Danyi Elavagnon; verbatimElevation: 769; verbatimSRS: WGS84; decimalLatitude: 7.2904; decimalLongitude: 0.707708; geodeticDatum: WGS84; **Identification:** identifiedBy: K. Akpagana; dateIdentified: /07/1987; **Event:** eventDate: /07/1987; habitat: Rainforest; **Record Level:** institutionID: Herbarium Togoense; collectionID: Akpagana, K.; institutionCode: TOGO; basisOfRecord: Preserved specimen

##### Ecological interactions

###### Native status

Native

##### Distribution

Zone 4

#### Asplenium inaequilaterale

Bory ex Willd.

##### Materials

- **Type status:** Other material. **Occurrence:** recordNumber: ASM 0155; recordedBy: Abotsi, K.E., Sodjinou E. & Mingou P.; **Taxon:** scientificName: Asplenium inaequilaterale Bory ex Willd.; namePublishedIn: Linn. Sp. Pl. ed. 4, 5: 322 (1810); kingdom: Plantae; phylum: Pteridophyta; class: Polypodiopsida; order: Polypodiales; family: Aspleniaceae; genus: Asplenium; specificEpithet: inaequilaterale; scientificNameAuthorship: Bory ex Willd.; **Location:** continent: Africa; country: Togo; countryCode: TG; locality: Dikpéléou; verbatimElevation: 723; verbatimSRS: WGS84; decimalLatitude: 8.196159386; decimalLongitude: 0.616072241; geodeticDatum: WGS84; **Identification:** identifiedBy: Abotsi, K.E.; dateIdentified: 05-09-13; **Event:** eventDate: 05-09-13; habitat: Rainforest; **Record Level:** institutionID: Herbarium Togoense; collectionID: Abotsi, K.E.; institutionCode: TOGO; basisOfRecord: Preserved specimen
- **Type status:** Other material. **Occurrence:** recordNumber: ASM 0171; recordedBy: Abotsi, K.E., Sodjinou E. & Mingou P.; **Taxon:** scientificName: Asplenium inaequilaterale Bory ex Willd.; namePublishedIn: Linn. Sp. Pl. ed. 4, 5: 322 (1810); kingdom: Plantae; phylum: Pteridophyta; class: Polypodiopsida; order: Polypodiales; family: Aspleniaceae; genus: Asplenium; specificEpithet: inaequilaterale; scientificNameAuthorship: Bory ex Willd.; **Location:** continent: Africa; country: Togo; countryCode: TG; locality: Assoukoko forest reserve, river Kpakrassou; verbatimElevation: 397; verbatimSRS: WGS84; decimalLatitude: 8.015147099; decimalLongitude: 0.633241708; geodeticDatum: WGS84; **Identification:** identifiedBy: Abotsi, K.E.; dateIdentified: 05-11-13; **Event:** eventDate: 05-11-13; habitat: Rainforest; **Record Level:** institutionID: Herbarium Togoense; collectionID: Abotsi, K.E.; institutionCode: TOGO; basisOfRecord: Preserved specimen
- **Type status:** Other material. **Occurrence:** recordNumber: ASM 0172; recordedBy: Abotsi, K.E., Sodjinou E. & Mingou P.; **Taxon:** scientificName: Asplenium inaequilaterale Bory ex Willd.; namePublishedIn: Linn. Sp. Pl. ed. 4, 5: 322 (1810); kingdom: Plantae; phylum: Pteridophyta; class: Polypodiopsida; order: Polypodiales; family: Aspleniaceae; genus: Asplenium; specificEpithet: inaequilaterale; scientificNameAuthorship: Bory ex Willd.; **Location:** continent: Africa; country: Togo; countryCode: TG; locality: Assoukoko forest reserve, river Kpakrassou; verbatimElevation: 397; verbatimSRS: WGS84; decimalLatitude: 8.015147099; decimalLongitude: 0.633241708; geodeticDatum: WGS84; **Identification:** identifiedBy: Abotsi, K.E.; dateIdentified: 05-11-13; **Event:** eventDate: 05-11-13; habitat: Rainforest; **Record Level:** institutionID: Herbarium Togoense; collectionID: Abotsi, K.E.; institutionCode: TOGO; basisOfRecord: Preserved specimen

##### Ecological interactions

###### Native status

Native

##### Distribution

Zone 4

#### Asplenium longicauda

Hook.

##### Materials

- **Type status:** Other material. **Occurrence:** recordNumber: AB0451; recordedBy: Abotsi, K.E.; **Taxon:** scientificName: Asplenium longicauda Hook.; namePublishedIn: 2. Cent. Ferns, t. 69 (1861); kingdom: Plantae; phylum: Pteridophyta; class: Polypodiopsida; order: Polypodiales; family: Aspleniaceae; genus: Asplenium; specificEpithet: longicauda; scientificNameAuthorship: Hook.; **Location:** continent: Africa; country: Togo; countryCode: TG; locality: Agomé Yoh; verbatimElevation: 355; verbatimSRS: WGS84; decimalLatitude: 6.949376; decimalLongitude: 0.596927; geodeticDatum: WGS84; **Identification:** identifiedBy: Abotsi, K.E.; dateIdentified: 12-29-16; **Event:** eventDate: 12-29-16; habitat: Rainforest; **Record Level:** institutionID: Herbarium Togoense; collectionID: Abotsi, K.E.; institutionCode: TOGO; basisOfRecord: Preserved specimen

##### Ecological interactions

###### Native status

Native

##### Distribution

Zone 4

#### Asplenium megalura

Hieron.

##### Materials

- **Type status:** Other material. **Occurrence:** recordNumber: AB0473; recordedBy: Abotsi, K.E.; **Taxon:** scientificName: Asplenium megalura Hieron.; namePublishedIn: Deutsche Zentralafr. Exp. 2: 17 (1910); kingdom: Plantae; phylum: Pteridophyta; class: Polypodiopsida; order: Polypodiales; family: Aspleniaceae; genus: Asplenium; specificEpithet: megalura; scientificNameAuthorship: Hieron.; **Location:** continent: Africa; country: Togo; countryCode: TG; locality: Danyi Dzogbégan; verbatimElevation: 715; verbatimSRS: WGS84; decimalLatitude: 7.232035; decimalLongitude: 0.677682; geodeticDatum: WGS84; **Identification:** identifiedBy: Abotsi, K.E.; dateIdentified: 04-24-17; **Event:** eventDate: 04-24-17; habitat: Rainforest; **Record Level:** institutionID: Herbarium Togoense; collectionID: Abotsi, K.E.; institutionCode: TOGO; basisOfRecord: Preserved specimen

##### Ecological interactions

###### Native status

Native

##### Distribution

Zone 4

#### Asplenium nidus

L.

##### Materials

- **Type status:** Other material. **Occurrence:** recordedBy: Abotsi, K.E.; **Taxon:** scientificName: Asplenium nidus L.; namePublishedIn: Sp. 2: 1079 (1753); kingdom: Plantae; phylum: Pteridophyta; class: Polypodiopsida; order: Polypodiales; family: Aspleniaceae; genus: Asplenium; specificEpithet: nidus; scientificNameAuthorship: L.; **Location:** continent: Africa; country: Togo; countryCode: TG; locality: Lomé, in gardens; **Identification:** identifiedBy: Abotsi, K.E.; **Event:** habitat: Gardens; **Record Level:** basisOfRecord: Human observation

##### Ecological interactions

###### Native status

Not native

##### Distribution

In gardens

#### Asplenium stuhlmannii

Hieron.

##### Materials

- **Type status:** Other material. **Occurrence:** catalogNumber: 12215; recordNumber: 2142; recordedBy: Akpagana, K.; **Taxon:** scientificName: Asplenium stuhlmannii Hieron.; namePublishedIn: Engl., Pflw. Ostafr. C: 83 (1895); kingdom: Plantae; phylum: Pteridophyta; class: Polypodiopsida; order: Polypodiales; family: Aspleniaceae; genus: Asplenium; specificEpithet: stuhlmannii; scientificNameAuthorship: Hieron.; **Location:** continent: Africa; country: Togo; countryCode: TG; locality: Akébou mountain; verbatimElevation: 452; verbatimSRS: WGS84; decimalLatitude: 7.702966; decimalLongitude: 0.72096; geodeticDatum: WGS84; **Identification:** identifiedBy: K. Akpagana; dateIdentified: /02/1991; **Event:** eventDate: /02/1991; habitat: Rainforest; **Record Level:** institutionID: Herbarium Togoense; collectionID: Akpagana, K.; institutionCode: TOGO; basisOfRecord: Preserved specimen

##### Ecological interactions

###### Native status

Native

##### Distribution

Zone 4

#### Asplenium uhligii

Hieron.

##### Materials

- **Type status:** Other material. **Occurrence:** recordedBy: Roux, J.P.; **Taxon:** scientificName: Asplenium uhligii Hieron.; namePublishedIn: Engl. Jahrb. 441. 374 (1911); kingdom: Plantae; phylum: Pteridophyta; class: Polypodiopsida; order: Polypodiales; family: Aspleniaceae; genus: Asplenium; specificEpithet: uhligii; scientificNameAuthorship: Hieron.; **Location:** continent: Africa; country: Togo; countryCode: TG; **Event:** habitat: Rainforest; **Record Level:** basisOfRecord: Unknown; source: Synopsis of the Lycopodiophyta and Pteridophyta of Africa, Madagascar and neighbouring islands (Roux, 2009)

##### Ecological interactions

###### Native status

Native

##### Distribution

Zone 4

#### Asplenium variabilevar.paucijugum

(Ballard) Alston

##### Materials

- **Type status:** Other material. **Occurrence:** recordNumber: AB0520; recordedBy: Abotsi, K.E.; **Taxon:** scientificName: Asplenium variabile Hook. var. paucijugum (Ballard) Alston; namePublishedIn: Sp. 8: 93, t. 185 (1860); kingdom: Plantae; phylum: Pteridophyta; class: Polypodiopsida; order: Polypodiales; family: Aspleniaceae; genus: Asplenium; specificEpithet: variabile; infraspecificEpithet: paucijugum; taxonRank: Variety; scientificNameAuthorship: (Ballard) Alston; **Location:** continent: Africa; country: Togo; countryCode: TG; locality: Ona; verbatimElevation: 611; verbatimSRS: WGS84; decimalLatitude: 7.512907; decimalLongitude: 0.76419; geodeticDatum: WGS84; **Identification:** identifiedBy: Abotsi, K.E.; dateIdentified: 04-26-17; **Event:** eventDate: 04-26-17; habitat: Rainforest; **Record Level:** institutionID: Herbarium Togoense; collectionID: Abotsi, K.E.; institutionCode: TOGO; basisOfRecord: Preserved specimen
- **Type status:** Other material. **Occurrence:** recordNumber: AB0136; recordedBy: Abotsi, K.E.; **Taxon:** scientificName: Asplenium variabile Hook. var. paucijugum (Ballard) Alston; namePublishedIn: Sp. 8: 93, t. 185 (1860); kingdom: Plantae; phylum: Pteridophyta; class: Polypodiopsida; order: Polypodiales; family: Aspleniaceae; genus: Asplenium; specificEpithet: variabile; infraspecificEpithet: paucijugum; taxonRank: Variety; scientificNameAuthorship: (Ballard) Alston; **Location:** continent: Africa; country: Togo; countryCode: TG; locality: Danyi N'Digbé; verbatimElevation: 581; verbatimSRS: WGS84; decimalLatitude: 7.115811; decimalLongitude: 0.66138; geodeticDatum: WGS84; **Identification:** identifiedBy: Abotsi, K.E.; dateIdentified: 06-28-16; **Event:** eventDate: 06-28-16; habitat: Rainforest; **Record Level:** institutionID: Herbarium Togoense; collectionID: Abotsi, K.E.; institutionCode: TOGO; basisOfRecord: Preserved specimen

##### Ecological interactions

###### Native status

Native

##### Distribution

Zone 4

#### Asplenium variabilevar.variabile

Hook.

##### Materials

- **Type status:** Other material. **Occurrence:** catalogNumber: 12222; recordNumber: 1379; recordedBy: Akpagana, K.; **Taxon:** scientificName: Asplenium variabile Hook. var. variabile Hook.; namePublishedIn: Bol. Soc. Brot. ser. 2, 30: 7 (1956); kingdom: Plantae; phylum: Pteridophyta; class: Polypodiopsida; order: Polypodiales; family: Aspleniaceae; genus: Asplenium; specificEpithet: variabile; infraspecificEpithet: variabile; taxonRank: Variety; scientificNameAuthorship: Hook.; **Location:** continent: Africa; country: Togo; countryCode: TG; locality: Danyi N'Digbé; verbatimElevation: 704; verbatimSRS: WGS84; decimalLatitude: 7.133; decimalLongitude: 0.669777; geodeticDatum: WGS84; **Identification:** identifiedBy: K. Akpagana; dateIdentified: /02/1987; **Event:** eventDate: /02/1987; habitat: Rainforest; **Record Level:** institutionID: Herbarium Togoense; collectionID: Akpagana, K.; institutionCode: TOGO; basisOfRecord: Preserved specimen
- **Type status:** Other material. **Occurrence:** catalogNumber: 12223; recordNumber: 1479; recordedBy: Akpagana, K.; **Taxon:** scientificName: Asplenium variabile Hook. var. variabile Hook.; namePublishedIn: Bol. Soc. Brot. ser. 2, 30: 7 (1956); kingdom: Plantae; phylum: Pteridophyta; class: Polypodiopsida; order: Polypodiales; family: Aspleniaceae; genus: Asplenium; specificEpithet: variabile; infraspecificEpithet: variabile; taxonRank: Variety; scientificNameAuthorship: Hook.; **Location:** continent: Africa; country: Togo; countryCode: TG; locality: Gbadi N'Kugna; verbatimElevation: 679; verbatimSRS: WGS84; decimalLatitude: 7.4541; decimalLongitude: 0.70565; geodeticDatum: WGS84; **Identification:** identifiedBy: K. Akpagana; dateIdentified: /03/1987; **Event:** eventDate: /03/1987; habitat: Rainforest; **Record Level:** institutionID: Herbarium Togoense; collectionID: Akpagana, K.; institutionCode: TOGO; basisOfRecord: Preserved specimen
- **Type status:** Other material. **Occurrence:** catalogNumber: 12224; recordNumber: 1562; recordedBy: Akpagana, K.; **Taxon:** scientificName: Asplenium variabile Hook. var. variabile Hook.; namePublishedIn: Bol. Soc. Brot. ser. 2, 30: 7 (1956); kingdom: Plantae; phylum: Pteridophyta; class: Polypodiopsida; order: Polypodiales; family: Aspleniaceae; genus: Asplenium; specificEpithet: variabile; infraspecificEpithet: variabile; taxonRank: Variety; scientificNameAuthorship: Hook.; **Location:** continent: Africa; country: Togo; countryCode: TG; locality: Danyi N'Digbé; verbatimElevation: 715; verbatimSRS: WGS84; decimalLatitude: 7.144703; decimalLongitude: 0.67574; geodeticDatum: WGS84; **Identification:** identifiedBy: K. Akpagana; dateIdentified: /03/1987; **Event:** eventDate: /03/1987; habitat: Rainforest; **Record Level:** institutionID: Herbarium Togoense; collectionID: Akpagana, K.; institutionCode: TOGO; basisOfRecord: Preserved specimen

##### Ecological interactions

###### Native status

Native

##### Distribution

Zone 4

#### Hymenasplenium unilaterale

(Lam.) Hayata

##### Materials

- **Type status:** Other material. **Occurrence:** recordNumber: AB0450; recordedBy: Abotsi, K.E.; **Taxon:** scientificName: Hymenasplenium unilaterale (Lam.) Hayata; namePublishedIn: Bot. Mag. (Tokyo) 41: 712 (1927); kingdom: Plantae; phylum: Pteridophyta; class: Polypodiopsida; order: Polypodiales; family: Aspleniaceae; genus: Hymenasplenium; specificEpithet: unilaterale; scientificNameAuthorship: (Lam.) Hayata; **Location:** continent: Africa; country: Togo; countryCode: TG; locality: Agomé Yoh; verbatimElevation: 350; verbatimSRS: WGS84; decimalLatitude: 6.949431; decimalLongitude: 0.596989; geodeticDatum: WGS84; **Identification:** identifiedBy: Abotsi, K.E.; dateIdentified: 12-29-16; **Event:** eventDate: 12-29-16; habitat: Rainforest; **Record Level:** institutionID: Herbarium Togoense; collectionID: Abotsi, K.E.; institutionCode: TOGO; basisOfRecord: Preserved specimen

##### Ecological interactions

###### Native status

Native

##### Distribution

Zone 4

#### Athyriaceae

#### Diplazium proliferum

(Lam.) Thou.

##### Materials

- **Type status:** Other material. **Occurrence:** recordNumber: ASM 0124B; recordedBy: Abotsi, K.E., Sodjinou E. & Mingou P.; **Taxon:** scientificName: Diplazium proliferum (Lam.) Thou.; namePublishedIn: Fl. Trist. d'Ac. 35 (1804); kingdom: Plantae; phylum: Pteridophyta; class: Polypodiopsida; order: Polypodiales; family: Athyriaceae; genus: Diplazium; specificEpithet: proliferum; scientificNameAuthorship: (Lam.) Thou.; **Location:** continent: Africa; country: Togo; countryCode: TG; locality: Yégué, river Atedia; verbatimElevation: 598; verbatimSRS: WGS84; decimalLatitude: 8.175000518; decimalLongitude: 0.659176219; geodeticDatum: WGS84; **Identification:** identifiedBy: Abotsi, K.E.; dateIdentified: 05-08-13; **Event:** eventDate: 05-08-13; habitat: Rainforest; **Record Level:** institutionID: Herbarium Togoense; collectionID: Abotsi, K.E.; institutionCode: TOGO; basisOfRecord: Preserved specimen
- **Type status:** Other material. **Occurrence:** recordNumber: ASM 0186; recordedBy: Abotsi, K.E., Sodjinou E. & Mingou P.; **Taxon:** scientificName: Diplazium proliferum (Lam.) Thou.; namePublishedIn: Fl. Trist. d'Ac. 35 (1804); kingdom: Plantae; phylum: Pteridophyta; class: Polypodiopsida; order: Polypodiales; family: Athyriaceae; genus: Diplazium; specificEpithet: proliferum; scientificNameAuthorship: (Lam.) Thou.; **Location:** continent: Africa; country: Togo; countryCode: TG; locality: Assoukoko forest reserve, river N'Gobo; verbatimElevation: 404; verbatimSRS: WGS84; decimalLatitude: 8.016297839; decimalLongitude: 0.632174175; geodeticDatum: WGS84; **Identification:** identifiedBy: Abotsi, K.E.; dateIdentified: 05-11-13; **Event:** eventDate: 05-11-13; habitat: Rainforest; **Record Level:** institutionID: Herbarium Togoense; collectionID: Abotsi, K.E.; institutionCode: TOGO; basisOfRecord: Preserved specimen
- **Type status:** Other material. **Occurrence:** recordNumber: AB0026; recordedBy: Abotsi, K.E.; **Taxon:** scientificName: Diplazium proliferum (Lam.) Thou.; namePublishedIn: Fl. Trist. d'Ac. 35 (1804); kingdom: Plantae; phylum: Pteridophyta; class: Polypodiopsida; order: Polypodiales; family: Athyriaceae; genus: Diplazium; specificEpithet: proliferum; scientificNameAuthorship: (Lam.) Thou.; **Location:** continent: Africa; country: Togo; countryCode: TG; locality: Agou Kébo-Dalavé; verbatimElevation: 506; verbatimSRS: WGS84; decimalLatitude: 6.857501; decimalLongitude: 0.752499; geodeticDatum: WGS84; **Identification:** identifiedBy: Abotsi, K.E.; dateIdentified: 06-14-16; **Event:** eventDate: 06-14-16; habitat: Rainforest; **Record Level:** institutionID: Herbarium Togoense; collectionID: Abotsi, K.E.; institutionCode: TOGO; basisOfRecord: Preserved specimen

##### Ecological interactions

###### Native status

Native

##### Distribution

Zone 4

#### Thelypteridaceae

#### Christella dentata

(Forssk.) Brownsey & Jermy

##### Materials

- **Type status:** Other material. **Occurrence:** recordNumber: AB0335; recordedBy: Abotsi, K.E.; **Taxon:** scientificName: Christella dentata (Forssk.) Brownsey & Jermy; namePublishedIn: Brit. Fern Gaz. 10: 338 (1973); kingdom: Plantae; phylum: Pteridophyta; class: Polypodiopsida; order: Polypodiales; family: Thelypteridaceae; genus: Christella; specificEpithet: dentata; scientificNameAuthorship: (Forssk.) Brownsey & Jermy; **Location:** continent: Africa; country: Togo; countryCode: TG; locality: Danyi Kudzragan; verbatimElevation: 691; verbatimSRS: WGS84; decimalLatitude: 7.150072; decimalLongitude: 0.623726; geodeticDatum: WGS84; **Identification:** identifiedBy: Abotsi, K.E.; dateIdentified: 09-30-16; **Event:** eventDate: 09-30-16; habitat: Rainforest; **Record Level:** institutionID: Herbarium Togoense; collectionID: Abotsi, K.E.; institutionCode: TOGO; basisOfRecord: Preserved specimen
- **Type status:** Other material. **Occurrence:** recordNumber: AB0507; recordedBy: Abotsi, K.E.; **Taxon:** scientificName: Christella dentata (Forssk.) Brownsey & Jermy; namePublishedIn: Brit. Fern Gaz. 10: 338 (1973); kingdom: Plantae; phylum: Pteridophyta; class: Polypodiopsida; order: Polypodiales; family: Thelypteridaceae; genus: Christella; specificEpithet: dentata; scientificNameAuthorship: (Forssk.) Brownsey & Jermy; **Location:** continent: Africa; country: Togo; countryCode: TG; locality: Gbadi Gawodo; verbatimElevation: 640; verbatimSRS: WGS84; decimalLatitude: 7.484785; decimalLongitude: 0.766352; geodeticDatum: WGS84; **Identification:** identifiedBy: Abotsi, K.E.; dateIdentified: 04-26-17; **Event:** eventDate: 04-26-17; habitat: Rainforest; **Record Level:** institutionID: Herbarium Togoense; collectionID: Abotsi, K.E.; institutionCode: TOGO; basisOfRecord: Preserved specimen
- **Type status:** Other material. **Occurrence:** recordNumber: AB0574; recordedBy: Abotsi, K.E.; **Taxon:** scientificName: Christella dentata (Forssk.) Brownsey & Jermy; namePublishedIn: Brit. Fern Gaz. 10: 338 (1973); kingdom: Plantae; phylum: Pteridophyta; class: Polypodiopsida; order: Polypodiales; family: Thelypteridaceae; genus: Christella; specificEpithet: dentata; scientificNameAuthorship: (Forssk.) Brownsey & Jermy; **Location:** continent: Africa; country: Togo; countryCode: TG; locality: Souroukou; verbatimElevation: 239; verbatimSRS: WGS84; decimalLatitude: 8.755171; decimalLongitude: 0.684436; geodeticDatum: WGS84; **Identification:** identifiedBy: Abotsi, K.E.; dateIdentified: 07-15-17; **Event:** eventDate: 07-15-17; habitat: Dry dense forest; **Record Level:** institutionID: Herbarium Togoense; collectionID: Abotsi, K.E.; institutionCode: TOGO; basisOfRecord: Preserved specimen

##### Ecological interactions

###### Native status

Native

##### Distribution

Zones 2, 3 and 4

#### Christella guineensis

(Christ) Holttum

##### Materials

- **Type status:** Other material. **Occurrence:** recordNumber: ASM 0255; recordedBy: Abotsi, K.E.; **Taxon:** scientificName: Christella guineensis (Christ) Holttum; namePublishedIn: J. S. Afr. Bot. 40: 145 (1974); kingdom: Plantae; phylum: Pteridophyta; class: Polypodiopsida; order: Polypodiales; family: Thelypteridaceae; genus: Christella; specificEpithet: guineensis; scientificNameAuthorship: (Christ) Holttum; **Location:** continent: Africa; country: Togo; countryCode: TG; locality: Danyi Dzogbégan; verbatimElevation: 786; verbatimSRS: WGS84; decimalLatitude: 7.236561041; decimalLongitude: 0.688195106; geodeticDatum: WGS84; **Identification:** identifiedBy: Abotsi, K.E.; dateIdentified: 04-10-13; **Event:** eventDate: 04-10-13; habitat: Rainforest; **Record Level:** institutionID: Herbarium Togoense; collectionID: Abotsi, K.E.; institutionCode: TOGO; basisOfRecord: Preserved specimen

##### Ecological interactions

###### Native status

Native

##### Distribution

Zone 4

#### Cyclosorus interruptus

(Willd.) H. Itô

##### Materials

- **Type status:** Other material. **Occurrence:** recordNumber: AB0131; recordedBy: Abotsi, K.E.; **Taxon:** scientificName: Cyclosorus interruptus (Willd.) H. Itô; namePublishedIn: Bot. Mag. (Tokyo) 51: 714 (1937); kingdom: Plantae; phylum: Pteridophyta; class: Polypodiopsida; order: Polypodiales; family: Thelypteridaceae; genus: Cyclosorus; specificEpithet: interruptus; scientificNameAuthorship: (Willd.) H. Itô; **Location:** continent: Africa; country: Togo; countryCode: TG; locality: Danyi N'Digbé; verbatimElevation: 600; verbatimSRS: WGS84; decimalLatitude: 7.115773; decimalLongitude: 0.661284; geodeticDatum: WGS84; **Identification:** identifiedBy: Abotsi, K.E.; dateIdentified: 06-28-16; **Event:** eventDate: 06-28-16; habitat: Coffee/cocoa based agroforest; **Record Level:** institutionID: Herbarium Togoense; collectionID: Abotsi, K.E.; institutionCode: TOGO; basisOfRecord: Preserved specimen
- **Type status:** Other material. **Occurrence:** recordNumber: AB0351; recordedBy: Abotsi, K.E.; **Taxon:** scientificName: Cyclosorus interruptus (Willd.) H. Itô; namePublishedIn: Bot. Mag. (Tokyo) 51: 714 (1937); kingdom: Plantae; phylum: Pteridophyta; class: Polypodiopsida; order: Polypodiales; family: Thelypteridaceae; genus: Cyclosorus; specificEpithet: interruptus; scientificNameAuthorship: (Willd.) H. Itô; **Location:** continent: Africa; country: Togo; countryCode: TG; locality: Danyi Gbaladzé; verbatimElevation: 796; verbatimSRS: WGS84; decimalLatitude: 7.208385; decimalLongitude: 0.731784; geodeticDatum: WGS84; **Identification:** identifiedBy: Abotsi, K.E.; dateIdentified: 10-01-16; **Event:** eventDate: 10-01-16; habitat: Rainforest; **Record Level:** institutionID: Herbarium Togoense; collectionID: Abotsi, K.E.; institutionCode: TOGO; basisOfRecord: Preserved specimen

##### Ecological interactions

###### Native status

Native

##### Distribution

Zone 4

#### Cyclosorus striatus

(Schum.) Ching

##### Materials

- **Type status:** Other material. **Occurrence:** recordNumber: AB0151; recordedBy: Abotsi, K.E.; **Taxon:** scientificName: Cyclosorus striatus (Schum.) Ching; namePublishedIn: Bull. Fan Mem. Inst. Biol. Bot. 10: 249 (1941); kingdom: Plantae; phylum: Pteridophyta; class: Polypodiopsida; order: Polypodiales; family: Thelypteridaceae; genus: Cyclosorus; specificEpithet: striatus; scientificNameAuthorship: (Schum.) Ching; **Location:** continent: Africa; country: Togo; countryCode: TG; locality: Aklakougan; verbatimElevation: 10; verbatimSRS: WGS84; decimalLatitude: 6.347992; decimalLongitude: 1.715628; geodeticDatum: WGS84; **Identification:** identifiedBy: Abotsi, K.E.; dateIdentified: 07-12-16; **Event:** eventDate: 07-12-16; habitat: Mangrove; **Record Level:** institutionID: Herbarium Togoense; collectionID: Abotsi, K.E.; institutionCode: TOGO; basisOfRecord: Preserved specimen
- **Type status:** Other material. **Occurrence:** recordNumber: AB0175; recordedBy: Abotsi, K.E.; **Taxon:** scientificName: Cyclosorus striatus (Schum.) Ching; namePublishedIn: Bull. Fan Mem. Inst. Biol. Bot. 10: 249 (1941); kingdom: Plantae; phylum: Pteridophyta; class: Polypodiopsida; order: Polypodiales; family: Thelypteridaceae; genus: Cyclosorus; specificEpithet: striatus; scientificNameAuthorship: (Schum.) Ching; **Location:** continent: Africa; country: Togo; countryCode: TG; locality: Volové; verbatimElevation: 224; verbatimSRS: WGS84; decimalLatitude: 6.887768; decimalLongitude: 0.619544; geodeticDatum: WGS84; **Identification:** identifiedBy: Abotsi, K.E.; dateIdentified: 08-02-16; **Event:** eventDate: 08-02-16; habitat: Floating meadow; **Record Level:** institutionID: Herbarium Togoense; collectionID: Abotsi, K.E.; institutionCode: TOGO; basisOfRecord: Preserved specimen
- **Type status:** Other material. **Occurrence:** recordNumber: AB0460; recordedBy: Abotsi, K.E.; **Taxon:** scientificName: Cyclosorus striatus (Schum.) Ching; namePublishedIn: Bull. Fan Mem. Inst. Biol. Bot. 10: 249 (1941); kingdom: Plantae; phylum: Pteridophyta; class: Polypodiopsida; order: Polypodiales; family: Thelypteridaceae; genus: Cyclosorus; specificEpithet: striatus; scientificNameAuthorship: (Schum.) Ching; **Location:** continent: Africa; country: Togo; countryCode: TG; locality: Afito; verbatimElevation: 31; verbatimSRS: WGS84; decimalLatitude: 6.754644; decimalLongitude: 1.590446; geodeticDatum: WGS84; **Identification:** identifiedBy: Abotsi, K.E.; dateIdentified: 01-17-17; **Event:** eventDate: 01-17-17; habitat: Floating meadow; **Record Level:** institutionID: Herbarium Togoense; collectionID: Abotsi, K.E.; institutionCode: TOGO; basisOfRecord: Preserved specimen

##### Ecological interactions

###### Native status

Native

##### Distribution

Zones 4 and 5

#### Pneumatopteris afra

(Christ) Holttum

##### Materials

- **Type status:** Other material. **Occurrence:** recordNumber: AB0106; recordedBy: Abotsi, K.E.; **Taxon:** scientificName: Pneumatopteris afra (Christ) Holttum; namePublishedIn: Blumea 21: 306 (1973); kingdom: Plantae; phylum: Pteridophyta; class: Polypodiopsida; order: Polypodiales; family: Thelypteridaceae; genus: Pneumatopteris; specificEpithet: afra; scientificNameAuthorship: (Christ) Holttum; **Location:** continent: Africa; country: Togo; countryCode: TG; locality: Danyi N'Digbé; verbatimElevation: 687; verbatimSRS: WGS84; decimalLatitude: 7.150936; decimalLongitude: 0.672439; geodeticDatum: WGS84; **Identification:** identifiedBy: Abotsi, K.E.; dateIdentified: 06-27-16; **Event:** eventDate: 06-27-16; habitat: Rainforest; **Record Level:** institutionID: Herbarium Togoense; collectionID: Abotsi, K.E.; institutionCode: TOGO; basisOfRecord: Preserved specimen
- **Type status:** Other material. **Occurrence:** recordNumber: AB0242; recordedBy: Abotsi, K.E.; **Taxon:** scientificName: Pneumatopteris afra (Christ) Holttum; namePublishedIn: Blumea 21: 306 (1973); kingdom: Plantae; phylum: Pteridophyta; class: Polypodiopsida; order: Polypodiales; family: Thelypteridaceae; genus: Pneumatopteris; specificEpithet: afra; scientificNameAuthorship: (Christ) Holttum; **Location:** continent: Africa; country: Togo; countryCode: TG; locality: Akloa; verbatimElevation: 361; verbatimSRS: WGS84; decimalLatitude: 7.515003; decimalLongitude: 0.619685; geodeticDatum: WGS84; **Identification:** identifiedBy: Abotsi, K.E.; dateIdentified: 08-06-16; **Event:** eventDate: 08-06-16; habitat: Rainforest; **Record Level:** institutionID: Herbarium Togoense; collectionID: Abotsi, K.E.; institutionCode: TOGO; basisOfRecord: Preserved specimen
- **Type status:** Other material. **Occurrence:** recordNumber: AB0481; recordedBy: Abotsi, K.E.; **Taxon:** scientificName: Pneumatopteris afra (Christ) Holttum; namePublishedIn: Blumea 21: 306 (1973); kingdom: Plantae; phylum: Pteridophyta; class: Polypodiopsida; order: Polypodiales; family: Thelypteridaceae; genus: Pneumatopteris; specificEpithet: afra; scientificNameAuthorship: (Christ) Holttum; **Location:** continent: Africa; country: Togo; countryCode: TG; locality: Ediwlou; verbatimElevation: 583; verbatimSRS: WGS84; decimalLatitude: 7.407942; decimalLongitude: 0.701539; geodeticDatum: WGS84; **Identification:** identifiedBy: Abotsi, K.E.; dateIdentified: 04-25-17; **Event:** eventDate: 04-25-17; habitat: Coffee/cocoa based agroforest; **Record Level:** institutionID: Herbarium Togoense; collectionID: Abotsi, K.E.; institutionCode: TOGO; basisOfRecord: Preserved specimen

##### Ecological interactions

###### Native status

Native

##### Distribution

Zone 4

#### Pneumatopteris oppositifolia

(Hook.) Holttum

##### Materials

- **Type status:** Other material. **Occurrence:** recordNumber: AB0197; recordedBy: Abotsi, K.E.; **Taxon:** scientificName: Pneumatopteris oppositifolia (Hook.) Holttum; namePublishedIn: Blumea 21: 304 (1973); kingdom: Plantae; phylum: Pteridophyta; class: Polypodiopsida; order: Polypodiales; family: Thelypteridaceae; genus: Pneumatopteris; specificEpithet: oppositifolia; scientificNameAuthorship: (Hook.) Holttum; **Location:** continent: Africa; country: Togo; countryCode: TG; locality: Kpimé Séva; verbatimElevation: 309; verbatimSRS: WGS84; decimalLatitude: 7.00841; decimalLongitude: 0.64558; geodeticDatum: WGS84; **Identification:** identifiedBy: Abotsi, K.E.; dateIdentified: 08-04-16; **Event:** eventDate: 08-04-16; habitat: Rainforest; **Record Level:** institutionID: Herbarium Togoense; collectionID: Abotsi, K.E.; institutionCode: TOGO; basisOfRecord: Preserved specimen
- **Type status:** Other material. **Occurrence:** recordNumber: AB0494; recordedBy: Abotsi, K.E.; **Taxon:** scientificName: Pneumatopteris oppositifolia (Hook.) Holttum; namePublishedIn: Blumea 21: 304 (1973); kingdom: Plantae; phylum: Pteridophyta; class: Polypodiopsida; order: Polypodiales; family: Thelypteridaceae; genus: Pneumatopteris; specificEpithet: oppositifolia; scientificNameAuthorship: (Hook.) Holttum; **Location:** continent: Africa; country: Togo; countryCode: TG; locality: Agbo Kopé; verbatimElevation: 666; verbatimSRS: WGS84; decimalLatitude: 7.453796; decimalLongitude: 0.6768; geodeticDatum: WGS84; **Identification:** identifiedBy: Abotsi, K.E.; dateIdentified: 04-25-17; **Event:** eventDate: 04-25-17; habitat: Rainforest; **Record Level:** institutionID: Herbarium Togoense; collectionID: Abotsi, K.E.; institutionCode: TOGO; basisOfRecord: Preserved specimen
- **Type status:** Other material. **Occurrence:** recordNumber: AB0602; recordedBy: Abotsi, K.E.; **Taxon:** scientificName: Pneumatopteris oppositifolia (Hook.) Holttum; namePublishedIn: Blumea 21: 304 (1973); kingdom: Plantae; phylum: Pteridophyta; class: Polypodiopsida; order: Polypodiales; family: Thelypteridaceae; genus: Pneumatopteris; specificEpithet: oppositifolia; scientificNameAuthorship: (Hook.) Holttum; **Location:** continent: Africa; country: Togo; countryCode: TG; locality: Diomé; verbatimElevation: 272; verbatimSRS: WGS84; decimalLatitude: 8.584623; decimalLongitude: 1.306073; geodeticDatum: WGS84; **Identification:** identifiedBy: Abotsi, K.E.; dateIdentified: 07-18-17; **Event:** eventDate: 07-18-17; habitat: Dry dense forest; **Record Level:** institutionID: Herbarium Togoense; collectionID: Abotsi, K.E.; institutionCode: TOGO; basisOfRecord: Preserved specimen

##### Ecological interactions

###### Native status

Native

##### Distribution

Zones 3 and 4

#### Pneumatopteris subpennigera

(C. Chr.) Holttum

##### Materials

- **Type status:** Other material. **Occurrence:** catalogNumber: 12444; recordNumber: 41; recordedBy: Akpagana, K.; **Taxon:** scientificName: Pneumatopteris subpennigera (C. Chr.) Holttum; namePublishedIn: Blumea 21: 304 (1973); kingdom: Plantae; phylum: Pteridophyta; class: Polypodiopsida; order: Polypodiales; family: Thelypteridaceae; genus: Pneumatopteris; specificEpithet: subpennigera; scientificNameAuthorship: (C. Chr.) Holttum; **Location:** continent: Africa; country: Togo; countryCode: TG; locality: Kouma Konda, "Campement Kloto"; verbatimElevation: 645; verbatimSRS: WGS84; decimalLatitude: 6.9415; decimalLongitude: 0.57489; geodeticDatum: WGS84; **Identification:** identifiedBy: K. Akpagana; dateIdentified: /4/1981; **Event:** eventDate: /4/1981; habitat: Rainforest; **Record Level:** institutionID: Herbarium Togoense; collectionID: Akpagana, K.; institutionCode: TOGO; basisOfRecord: Preserved specimen

##### Ecological interactions

###### Native status

Native

##### Distribution

Zone 4

#### Dennstaedtiineae

#### Dennstaedtiaceae

#### Blotiella currorii

(Hook.) R.M.Tryon

##### Materials

- **Type status:** Other material. **Occurrence:** recordNumber: AB0417; recordedBy: Abotsi, K.E.; **Taxon:** scientificName: Blotiella currorii (Hook.) R.M.Tryon; namePublishedIn: Contr. Gray Herb. 191: 99 (1962); kingdom: Plantae; phylum: Pteridophyta; class: Polypodiopsida; order: Polypodiales; family: Dennstaedtiaceae; genus: Blotiella; specificEpithet: currorii; scientificNameAuthorship: (Hook.) R.M.Tryon; **Location:** continent: Africa; country: Togo; countryCode: TG; locality: Danyi Dzogbégan; verbatimElevation: 715; verbatimSRS: WGS84; decimalLatitude: 7.232104; decimalLongitude: 0.67749; geodeticDatum: WGS84; **Identification:** identifiedBy: Abotsi, K.E.; dateIdentified: 12-27-16; **Event:** eventDate: 12-27-16; habitat: Rainforest; **Record Level:** institutionID: Herbarium Togoense; collectionID: Abotsi, K.E.; institutionCode: TOGO; basisOfRecord: Preserved specimen
- **Type status:** Other material. **Occurrence:** recordNumber: AB0431; recordedBy: Abotsi, K.E.; **Taxon:** scientificName: Blotiella currorii (Hook.) R.M.Tryon; namePublishedIn: Contr. Gray Herb. 191: 99 (1962); kingdom: Plantae; phylum: Pteridophyta; class: Polypodiopsida; order: Polypodiales; family: Dennstaedtiaceae; genus: Blotiella; specificEpithet: currorii; scientificNameAuthorship: (Hook.) R.M.Tryon; **Location:** continent: Africa; country: Togo; countryCode: TG; locality: Danyi Afiadégnigba; verbatimElevation: 771; verbatimSRS: WGS84; decimalLatitude: 7.293651; decimalLongitude: 0.710834; geodeticDatum: WGS84; **Identification:** identifiedBy: Abotsi, K.E.; dateIdentified: 12-27-16; **Event:** eventDate: 12-27-16; habitat: Rainforest; **Record Level:** institutionID: Herbarium Togoense; collectionID: Abotsi, K.E.; institutionCode: TOGO; basisOfRecord: Preserved specimen
- **Type status:** Other material. **Occurrence:** recordNumber: AB0488; recordedBy: Abotsi, K.E.; **Taxon:** scientificName: Blotiella currorii (Hook.) R.M.Tryon; namePublishedIn: Contr. Gray Herb. 191: 99 (1962); kingdom: Plantae; phylum: Pteridophyta; class: Polypodiopsida; order: Polypodiales; family: Dennstaedtiaceae; genus: Blotiella; specificEpithet: currorii; scientificNameAuthorship: (Hook.) R.M.Tryon; **Location:** continent: Africa; country: Togo; countryCode: TG; locality: Agbo Kopé; verbatimElevation: 667; verbatimSRS: WGS84; decimalLatitude: 7.453814; decimalLongitude: 0.676844; geodeticDatum: WGS84; **Identification:** identifiedBy: Abotsi, K.E.; dateIdentified: 04-25-17; **Event:** eventDate: 04-25-17; habitat: Rainforest; **Record Level:** institutionID: Herbarium Togoense; collectionID: Abotsi, K.E.; institutionCode: TOGO; basisOfRecord: Preserved specimen

##### Ecological interactions

###### Native status

Native

##### Distribution

Zone 4

#### Microlepia speluncae

(L.) Moore

##### Materials

- **Type status:** Other material. **Occurrence:** recordNumber: AB0104; recordedBy: Abotsi, K.E.; **Taxon:** scientificName: Microlepia speluncae (L.) Moore; namePublishedIn: Ind. Fil. 93 (1857); kingdom: Plantae; phylum: Pteridophyta; class: Polypodiopsida; order: Polypodiales; family: Dennstaedtiaceae; genus: Microlepia; specificEpithet: speluncae; scientificNameAuthorship: (L.) Moore; **Location:** continent: Africa; country: Togo; countryCode: TG; locality: Danyi N'Digbé; verbatimElevation: 695; verbatimSRS: WGS84; decimalLatitude: 7.150579; decimalLongitude: 0.672903; geodeticDatum: WGS84; **Identification:** identifiedBy: Abotsi, K.E.; dateIdentified: 06-27-16; **Event:** eventDate: 06-27-16; habitat: Rainforest; **Record Level:** institutionID: Herbarium Togoense; collectionID: Abotsi, K.E.; institutionCode: TOGO; basisOfRecord: Preserved specimen
- **Type status:** Other material. **Occurrence:** recordNumber: AB0243; recordedBy: Abotsi, K.E.; **Taxon:** scientificName: Microlepia speluncae (L.) Moore; namePublishedIn: Ind. Fil. 93 (1857); kingdom: Plantae; phylum: Pteridophyta; class: Polypodiopsida; order: Polypodiales; family: Dennstaedtiaceae; genus: Microlepia; specificEpithet: speluncae; scientificNameAuthorship: (L.) Moore; **Location:** continent: Africa; country: Togo; countryCode: TG; locality: Akloa; verbatimElevation: 353; verbatimSRS: WGS84; decimalLatitude: 7.514951; decimalLongitude: 0.619469; geodeticDatum: WGS84; **Identification:** identifiedBy: Abotsi, K.E.; dateIdentified: 08-06-16; **Event:** eventDate: 08-06-16; habitat: Rainforest; **Record Level:** institutionID: Herbarium Togoense; collectionID: Abotsi, K.E.; institutionCode: TOGO; basisOfRecord: Preserved specimen
- **Type status:** Other material. **Occurrence:** recordNumber: AB0314; recordedBy: Abotsi, K.E.; **Taxon:** scientificName: Microlepia speluncae (L.) Moore; namePublishedIn: Ind. Fil. 93 (1857); kingdom: Plantae; phylum: Pteridophyta; class: Polypodiopsida; order: Polypodiales; family: Dennstaedtiaceae; genus: Microlepia; specificEpithet: speluncae; scientificNameAuthorship: (L.) Moore; **Location:** continent: Africa; country: Togo; countryCode: TG; locality: Assoukoko; verbatimElevation: 596; verbatimSRS: WGS84; decimalLatitude: 8.003764; decimalLongitude: 0.625461; geodeticDatum: WGS84; **Identification:** identifiedBy: Abotsi, K.E.; dateIdentified: 09-18-16; **Event:** eventDate: 09-18-16; habitat: Rainforest; **Record Level:** institutionID: Herbarium Togoense; collectionID: Abotsi, K.E.; institutionCode: TOGO; basisOfRecord: Preserved specimen

##### Ecological interactions

###### Native status

Native

##### Distribution

Zone 4

#### Pteridium capense

(Thunb.) Krasser

##### Materials

- **Type status:** Other material. **Occurrence:** recordNumber: AB0081; recordedBy: Abotsi, K.E.; **Taxon:** scientificName: Pteridium capense (Thunb.) Krasser; namePublishedIn: Ann. Hofmus. Wien 15: 6 (1900); kingdom: Plantae; phylum: Pteridophyta; class: Polypodiopsida; order: Polypodiales; family: Dennstaedtiaceae; genus: Pteridium; specificEpithet: capense; scientificNameAuthorship: (Thunb.) Krasser; **Location:** continent: Africa; country: Togo; countryCode: TG; locality: Kuma Konda; verbatimElevation: 562; verbatimSRS: WGS84; decimalLatitude: 6.94931; decimalLongitude: 0.577096; geodeticDatum: WGS84; **Identification:** identifiedBy: Abotsi, K.E.; dateIdentified: 06-24-16; **Event:** eventDate: 06-24-16; habitat: Wooded savannah; **Record Level:** institutionID: Herbarium Togoense; collectionID: Abotsi, K.E.; institutionCode: TOGO; basisOfRecord: Preserved specimen
- **Type status:** Other material. **Occurrence:** recordNumber: AB0196; recordedBy: Abotsi, K.E.; **Taxon:** scientificName: Pteridium capense (Thunb.) Krasser; namePublishedIn: Ann. Hofmus. Wien 15: 6 (1900); kingdom: Plantae; phylum: Pteridophyta; class: Polypodiopsida; order: Polypodiales; family: Dennstaedtiaceae; genus: Pteridium; specificEpithet: capense; scientificNameAuthorship: (Thunb.) Krasser; **Location:** continent: Africa; country: Togo; countryCode: TG; locality: Womé; verbatimElevation: 355; verbatimSRS: WGS84; decimalLatitude: 6.858877; decimalLongitude: 0.556633; geodeticDatum: WGS84; **Identification:** identifiedBy: Abotsi, K.E.; dateIdentified: 08-03-16; **Event:** eventDate: 08-03-16; habitat: Wooded savannah; **Record Level:** institutionID: Herbarium Togoense; collectionID: Abotsi, K.E.; institutionCode: TOGO; basisOfRecord: Preserved specimen
- **Type status:** Other material. **Occurrence:** recordNumber: AB0377; recordedBy: Abotsi, K.E.; **Taxon:** scientificName: Pteridium capense (Thunb.) Krasser; namePublishedIn: Ann. Hofmus. Wien 15: 6 (1900); kingdom: Plantae; phylum: Pteridophyta; class: Polypodiopsida; order: Polypodiales; family: Dennstaedtiaceae; genus: Pteridium; specificEpithet: capense; scientificNameAuthorship: (Thunb.) Krasser; **Location:** continent: Africa; country: Togo; countryCode: TG; locality: Lalamila; verbatimElevation: 575; verbatimSRS: WGS84; decimalLatitude: 8.1645; decimalLongitude: 0.80856; geodeticDatum: WGS84; **Identification:** identifiedBy: Abotsi, K.E.; dateIdentified: 10-05-16; **Event:** eventDate: 10-05-16; habitat: Fallow; **Record Level:** institutionID: Herbarium Togoense; collectionID: Abotsi, K.E.; institutionCode: TOGO; basisOfRecord: Preserved specimen

##### Ecological interactions

###### Native status

Native

##### Distribution

Zone 4

#### Polypodiineae

#### Davalliaceae

#### Davallia chaerophylloides

(Poir.) Steud.

##### Materials

- **Type status:** Other material. **Occurrence:** recordNumber: AB0359; recordedBy: Abotsi, K.E.; **Taxon:** scientificName: Davallia chaerophylloides (Poir.) Steud.; namePublishedIn: Nom. 2. 146 (1824); kingdom: Plantae; phylum: Pteridophyta; class: Polypodiopsida; order: Polypodiales; family: Davalliaceae; genus: Davallia; specificEpithet: chaerophylloides; scientificNameAuthorship: (Poir.) Steud.; **Location:** continent: Africa; country: Togo; countryCode: TG; locality: Danyi Gbaladzé; verbatimElevation: 793; verbatimSRS: WGS84; decimalLatitude: 7.206387; decimalLongitude: 0.731235; geodeticDatum: WGS84; **Identification:** identifiedBy: Abotsi, K.E.; dateIdentified: 10-01-16; **Event:** eventDate: 10-01-16; habitat: Coffee/cocoa based agroforest; **Record Level:** institutionID: Herbarium Togoense; collectionID: Abotsi, K.E.; institutionCode: TOGO; basisOfRecord: Preserved specimen
- **Type status:** Other material. **Occurrence:** recordNumber: AB0085; recordedBy: Abotsi, K.E.; **Taxon:** scientificName: Davallia chaerophylloides (Poir.) Steud.; namePublishedIn: Nom. 2. 146 (1824); kingdom: Plantae; phylum: Pteridophyta; class: Polypodiopsida; order: Polypodiales; family: Davalliaceae; genus: Davallia; specificEpithet: chaerophylloides; scientificNameAuthorship: (Poir.) Steud.; **Location:** continent: Africa; country: Togo; countryCode: TG; locality: Kuma Konda; verbatimElevation: 569; verbatimSRS: WGS84; decimalLatitude: 6.948921; decimalLongitude: 0.578765; geodeticDatum: WGS84; **Identification:** identifiedBy: Abotsi, K.E.; dateIdentified: 06-24-16; **Event:** eventDate: 06-24-16; habitat: Rainforest; **Record Level:** institutionID: Herbarium Togoense; collectionID: Abotsi, K.E.; institutionCode: TOGO; basisOfRecord: Preserved specimen
- **Type status:** Other material. **Occurrence:** recordNumber: AB0144; recordedBy: Abotsi, K.E.; **Taxon:** scientificName: Davallia chaerophylloides (Poir.) Steud.; namePublishedIn: Nom. 2. 146 (1824); kingdom: Plantae; phylum: Pteridophyta; class: Polypodiopsida; order: Polypodiales; family: Davalliaceae; genus: Davallia; specificEpithet: chaerophylloides; scientificNameAuthorship: (Poir.) Steud.; **Location:** continent: Africa; country: Togo; countryCode: TG; locality: Danyi N'Digbé; verbatimElevation: 760; verbatimSRS: WGS84; decimalLatitude: 7.138132; decimalLongitude: 0.674497; geodeticDatum: WGS84; **Identification:** identifiedBy: Abotsi, K.E.; dateIdentified: 06-28-16; **Event:** eventDate: 06-28-16; habitat: Inhabited area, on a tree; **Record Level:** institutionID: Herbarium Togoense; collectionID: Abotsi, K.E.; institutionCode: TOGO; basisOfRecord: Preserved specimen

##### Ecological interactions

###### Native status

Native

##### Distribution

Zone 4

#### Davallia trichomanoides

Blume

##### Materials

- **Type status:** Other material. **Occurrence:** recordedBy: Abotsi, K.E.; **Taxon:** scientificName: Davallia trichomanoides Blume; namePublishedIn: Enum. Pl. Javae 2: 238. (1828); kingdom: Plantae; phylum: Pteridophyta; class: Polypodiopsida; order: Polypodiales; family: Davalliaceae; genus: Davallia; specificEpithet: trichomanoides; scientificNameAuthorship: Blume; **Location:** continent: Africa; country: Togo; countryCode: TG; locality: Lomé, in gardens; **Identification:** identifiedBy: Abotsi, K.E.; **Event:** habitat: Gardens; **Record Level:** basisOfRecord: Human observation

##### Ecological interactions

###### Native status

Not native

##### Distribution

In gardens

#### Dryopteridaceae

#### Dryopteridoideae

#### Ctenitis cirrhosa

(Schumach.) Ching

##### Materials

- **Type status:** Other material. **Occurrence:** recordNumber: AB0518; recordedBy: Abotsi, K.E.; **Taxon:** scientificName: Ctenitis cirrhosa (Schumach.) Ching; namePublishedIn: Sunyatsenia 5: 250 (1940); kingdom: Plantae; phylum: Pteridophyta; class: Polypodiopsida; order: Polypodiales; family: Dryopteridaceae; genus: Ctenitis; specificEpithet: cirrhosa; scientificNameAuthorship: (Schumach.) Ching; **Location:** continent: Africa; country: Togo; countryCode: TG; locality: Ona; verbatimElevation: 614; verbatimSRS: WGS84; decimalLatitude: 7.512781; decimalLongitude: 0.763979; geodeticDatum: WGS84; **Identification:** identifiedBy: Abotsi, K.E.; dateIdentified: 04-26-17; **Event:** eventDate: 04-26-17; habitat: Rainforest; **Record Level:** institutionID: Herbarium Togoense; collectionID: Abotsi, K.E.; institutionCode: TOGO; basisOfRecord: Preserved specimen
- **Type status:** Other material. **Occurrence:** recordNumber: AB0378; recordedBy: Abotsi, K.E.; **Taxon:** scientificName: Ctenitis cirrhosa (Schumach.) Ching; namePublishedIn: Sunyatsenia 5: 250 (1940); kingdom: Plantae; phylum: Pteridophyta; class: Polypodiopsida; order: Polypodiales; family: Dryopteridaceae; genus: Ctenitis; specificEpithet: cirrhosa; scientificNameAuthorship: (Schumach.) Ching; **Location:** continent: Africa; country: Togo; countryCode: TG; locality: Lalamila; verbatimElevation: 567; verbatimSRS: WGS84; decimalLatitude: 8.164788; decimalLongitude: 0.807486; geodeticDatum: WGS84; **Identification:** identifiedBy: Abotsi, K.E.; dateIdentified: 10-05-16; **Event:** eventDate: 10-05-16; habitat: Rainforest; **Record Level:** institutionID: Herbarium Togoense; collectionID: Abotsi, K.E.; institutionCode: TOGO; basisOfRecord: Preserved specimen
- **Type status:** Other material. **Occurrence:** recordNumber: ASM 0218; recordedBy: Abotsi, K.E., Sodjinou E. & Mingou P.; **Taxon:** scientificName: Ctenitis cirrhosa (Schumach.) Ching; namePublishedIn: Sunyatsenia 5: 250 (1940); kingdom: Plantae; phylum: Pteridophyta; class: Polypodiopsida; order: Polypodiales; family: Dryopteridaceae; genus: Ctenitis; specificEpithet: cirrhosa; scientificNameAuthorship: (Schumach.) Ching; **Location:** continent: Africa; country: Togo; countryCode: TG; locality: Danyi N'Digbé, river Fufu; verbatimElevation: 693; verbatimSRS: WGS84; decimalLatitude: 7.15439309; decimalLongitude: 0.669064082; geodeticDatum: WGS84; **Identification:** identifiedBy: Abotsi, K.E.; dateIdentified: 04-08-13; **Event:** eventDate: 04-08-13; habitat: Rainforest; **Record Level:** institutionID: Herbarium Togoense; collectionID: Abotsi, K.E.; institutionCode: TOGO; basisOfRecord: Preserved specimen

##### Ecological interactions

###### Native status

Native

##### Distribution

Zone 4

#### Dryopteris athamantica

(Kunze) Kuntze

##### Materials

- **Type status:** Other material. **Occurrence:** catalogNumber: 12113; recordNumber: 5589; recordedBy: Brunel, J.-F.; **Taxon:** scientificName: Dryopteris athamantica (Kunze) Kuntze; namePublishedIn: Rev. Gen. Pl. 2: 812 (1891); kingdom: Plantae; phylum: Pteridophyta; class: Polypodiopsida; order: Polypodiales; family: Dryopteridaceae; genus: Dryopteris; specificEpithet: athamantica; scientificNameAuthorship: (Kunze) Kuntze; **Location:** continent: Africa; country: Togo; countryCode: TG; locality: Yoh; verbatimElevation: 300; verbatimSRS: WGS84; decimalLatitude: 7; decimalLongitude: 0.75; geodeticDatum: WGS84; **Identification:** identifiedBy: Meyer, C.A.; dateIdentified: 1980; **Event:** eventDate: /10/1978; habitat: Rainforest; **Record Level:** institutionID: Herbarium Togoense; collectionID: Brunel, J.-F.; institutionCode: TOGO; basisOfRecord: Preserved specimen
- **Type status:** Other material. **Occurrence:** catalogNumber: 12114; recordNumber: 5589 bis; recordedBy: Brunel, J.-F.; **Taxon:** scientificName: Dryopteris athamantica (Kunze) Kuntze; namePublishedIn: Rev. Gen. Pl. 2: 812 (1891); kingdom: Plantae; phylum: Pteridophyta; class: Polypodiopsida; order: Polypodiales; family: Dryopteridaceae; genus: Dryopteris; specificEpithet: athamantica; scientificNameAuthorship: (Kunze) Kuntze; **Location:** continent: Africa; country: Togo; countryCode: TG; locality: Yoh; verbatimElevation: 300; verbatimSRS: WGS84; decimalLatitude: 7; decimalLongitude: 0.75; geodeticDatum: WGS84; **Identification:** identifiedBy: Meyer, C.A.; dateIdentified: 1980; **Event:** eventDate: /10/1978; habitat: Rainforest; **Record Level:** institutionID: Herbarium Togoense; collectionID: Brunel, J.-F.; institutionCode: TOGO; basisOfRecord: Preserved specimen

##### Ecological interactions

###### Native status

Native

##### Distribution

Zone 4

#### Elaphoglossoideae

#### Bolbitis acrostichoides

(Afzel. ex Sw.) Ching

##### Materials

- **Type status:** Other material. **Occurrence:** recordNumber: AB0327; recordedBy: Abotsi, K.E.; **Taxon:** scientificName: Bolbitis acrostichoides (Afzel. ex Sw.) Ching; namePublishedIn: C. Chr., Ind. Fil., Suppl. III: 47 (1934); kingdom: Plantae; phylum: Pteridophyta; class: Polypodiopsida; order: Polypodiales; family: Dryopteridaceae; genus: Bolbitis; specificEpithet: acrostichoides; scientificNameAuthorship: (Afzel. ex Sw.) Ching; **Location:** continent: Africa; country: Togo; countryCode: TG; locality: Assoukoko; verbatimElevation: 345; verbatimSRS: WGS84; decimalLatitude: 8.004729; decimalLongitude: 0.613121; geodeticDatum: WGS84; **Identification:** identifiedBy: Abotsi, K.E.; dateIdentified: 09-18-16; **Event:** eventDate: 09-18-16; habitat: Rainforest; **Record Level:** institutionID: Herbarium Togoense; collectionID: Abotsi, K.E.; institutionCode: TOGO; basisOfRecord: Preserved specimen
- **Type status:** Other material. **Occurrence:** recordNumber: AB0331; recordedBy: Abotsi, K.E.; **Taxon:** scientificName: Bolbitis acrostichoides (Afzel. ex Sw.) Ching; namePublishedIn: C. Chr., Ind. Fil., Suppl. III: 47 (1934); kingdom: Plantae; phylum: Pteridophyta; class: Polypodiopsida; order: Polypodiales; family: Dryopteridaceae; genus: Bolbitis; specificEpithet: acrostichoides; scientificNameAuthorship: (Afzel. ex Sw.) Ching; **Location:** continent: Africa; country: Togo; countryCode: TG; locality: Assoukoko; verbatimElevation: 346; verbatimSRS: WGS84; decimalLatitude: 8.003891; decimalLongitude: 0.613241; geodeticDatum: WGS84; **Identification:** identifiedBy: Abotsi, K.E.; dateIdentified: 09-18-16; **Event:** eventDate: 09-18-16; habitat: Rainforest; **Record Level:** institutionID: Herbarium Togoense; collectionID: Abotsi, K.E.; institutionCode: TOGO; basisOfRecord: Preserved specimen
- **Type status:** Other material. **Occurrence:** recordNumber: AB0394; recordedBy: Abotsi, K.E.; **Taxon:** scientificName: Bolbitis acrostichoides (Afzel. ex Sw.) Ching; namePublishedIn: C. Chr., Ind. Fil., Suppl. III: 47 (1934); kingdom: Plantae; phylum: Pteridophyta; class: Polypodiopsida; order: Polypodiales; family: Dryopteridaceae; genus: Bolbitis; specificEpithet: acrostichoides; scientificNameAuthorship: (Afzel. ex Sw.) Ching; **Location:** continent: Africa; country: Togo; countryCode: TG; locality: Diguengué; verbatimElevation: 427; verbatimSRS: WGS84; decimalLatitude: 8.071374; decimalLongitude: 0.6411; geodeticDatum: WGS84; **Identification:** identifiedBy: Abotsi, K.E.; dateIdentified: 10-06-16; **Event:** eventDate: 10-06-16; habitat: Rainforest; **Record Level:** institutionID: Herbarium Togoense; collectionID: Abotsi, K.E.; institutionCode: TOGO; basisOfRecord: Preserved specimen

##### Ecological interactions

###### Native status

Native

##### Distribution

Zone 4

#### Bolbitis auriculata

(Lam.) Alston

##### Materials

- **Type status:** Other material. **Occurrence:** recordNumber: AB0220; recordedBy: Abotsi, K.E.; **Taxon:** scientificName: Bolbitis auriculata (Lam.) Alston; namePublishedIn: J. Bot. Suppl. 3 (1934); kingdom: Plantae; phylum: Pteridophyta; class: Polypodiopsida; order: Polypodiales; family: Dryopteridaceae; genus: Bolbitis; specificEpithet: auriculata; scientificNameAuthorship: (Lam.) Alston; **Location:** continent: Africa; country: Togo; countryCode: TG; locality: Ayomé; verbatimElevation: 326; verbatimSRS: WGS84; decimalLatitude: 7.501178; decimalLongitude: 0.955729; geodeticDatum: WGS84; **Identification:** identifiedBy: Abotsi, K.E.; dateIdentified: 08-05-16; **Event:** eventDate: 08-05-16; habitat: Rainforest; **Record Level:** institutionID: Herbarium Togoense; collectionID: Abotsi, K.E.; institutionCode: TOGO; basisOfRecord: Preserved specimen
- **Type status:** Other material. **Occurrence:** recordNumber: AB0290; recordedBy: Abotsi, K.E.; **Taxon:** scientificName: Bolbitis auriculata (Lam.) Alston; namePublishedIn: J. Bot. Suppl. 3 (1934); kingdom: Plantae; phylum: Pteridophyta; class: Polypodiopsida; order: Polypodiales; family: Dryopteridaceae; genus: Bolbitis; specificEpithet: auriculata; scientificNameAuthorship: (Lam.) Alston; **Location:** continent: Africa; country: Togo; countryCode: TG; locality: Sérégbéné; verbatimElevation: 526; verbatimSRS: WGS84; decimalLatitude: 7.864947; decimalLongitude: 0.73967; geodeticDatum: WGS84; **Identification:** identifiedBy: Abotsi, K.E.; dateIdentified: 09-17-16; **Event:** eventDate: 09-17-16; habitat: Rainforest; **Record Level:** institutionID: Herbarium Togoense; collectionID: Abotsi, K.E.; institutionCode: TOGO; basisOfRecord: Preserved specimen
- **Type status:** Other material. **Occurrence:** recordNumber: AB0404; recordedBy: Abotsi, K.E.; **Taxon:** scientificName: Bolbitis auriculata (Lam.) Alston; namePublishedIn: J. Bot. Suppl. 3 (1934); kingdom: Plantae; phylum: Pteridophyta; class: Polypodiopsida; order: Polypodiales; family: Dryopteridaceae; genus: Bolbitis; specificEpithet: auriculata; scientificNameAuthorship: (Lam.) Alston; **Location:** continent: Africa; country: Togo; countryCode: TG; locality: Agou Kébo-Dzigbé; verbatimElevation: 804; verbatimSRS: WGS84; decimalLatitude: 6.864669; decimalLongitude: 0.755956; geodeticDatum: WGS84; **Identification:** identifiedBy: Abotsi, K.E.; dateIdentified: 12-26-16; **Event:** eventDate: 12-26-16; habitat: Rainforest; **Record Level:** institutionID: Herbarium Togoense; collectionID: Abotsi, K.E.; institutionCode: TOGO; basisOfRecord: Preserved specimen

##### Ecological interactions

###### Native status

Native

##### Distribution

Zone 4

#### Bolbitis heudelotii

(Bory ex Fée) Alston

##### Materials

- **Type status:** Other material. **Occurrence:** recordNumber: AB0119; recordedBy: Abotsi, K.E.; **Taxon:** scientificName: Bolbitis heudelotii (Bory ex Fée) Alston; namePublishedIn: Kew Bull. Suppl. 3 (1934); kingdom: Plantae; phylum: Pteridophyta; class: Polypodiopsida; order: Polypodiales; family: Dryopteridaceae; genus: Bolbitis; specificEpithet: heudelotii; scientificNameAuthorship: (Bory ex Fée) Alston; **Location:** continent: Africa; country: Togo; countryCode: TG; locality: Danyi N'Digbé; verbatimElevation: 679; verbatimSRS: WGS84; decimalLatitude: 7.15317; decimalLongitude: 0.666869; geodeticDatum: WGS84; **Identification:** identifiedBy: Abotsi, K.E.; dateIdentified: 06-27-16; **Event:** eventDate: 06-27-16; habitat: Rainforest; **Record Level:** institutionID: Herbarium Togoense; collectionID: Abotsi, K.E.; institutionCode: TOGO; basisOfRecord: Preserved specimen
- **Type status:** Other material. **Occurrence:** recordNumber: AB0409; recordedBy: Abotsi, K.E.; **Taxon:** scientificName: Bolbitis heudelotii (Bory ex Fée) Alston; namePublishedIn: Kew Bull. Suppl. 3 (1934); kingdom: Plantae; phylum: Pteridophyta; class: Polypodiopsida; order: Polypodiales; family: Dryopteridaceae; genus: Bolbitis; specificEpithet: heudelotii; scientificNameAuthorship: (Bory ex Fée) Alston; **Location:** continent: Africa; country: Togo; countryCode: TG; locality: Danyi Apéyémé; verbatimElevation: 691; verbatimSRS: WGS84; decimalLatitude: 7.184707; decimalLongitude: 0.691975; geodeticDatum: WGS84; **Identification:** identifiedBy: Abotsi, K.E.; dateIdentified: 12-27-16; **Event:** eventDate: 12-27-16; habitat: Rainforest; **Record Level:** institutionID: Herbarium Togoense; collectionID: Abotsi, K.E.; institutionCode: TOGO; basisOfRecord: Preserved specimen
- **Type status:** Other material. **Occurrence:** recordNumber: AB0579; recordedBy: Abotsi, K.E.; **Taxon:** scientificName: Bolbitis heudelotii (Bory ex Fée) Alston; namePublishedIn: Kew Bull. Suppl. 3 (1934); kingdom: Plantae; phylum: Pteridophyta; class: Polypodiopsida; order: Polypodiales; family: Dryopteridaceae; genus: Bolbitis; specificEpithet: heudelotii; scientificNameAuthorship: (Bory ex Fée) Alston; **Location:** continent: Africa; country: Togo; countryCode: TG; locality: Souroukou; verbatimElevation: 346; verbatimSRS: WGS84; decimalLatitude: 8.756288; decimalLongitude: 0.681726; geodeticDatum: WGS84; **Identification:** identifiedBy: Abotsi, K.E.; dateIdentified: 07-15-17; **Event:** eventDate: 07-15-17; habitat: Dry dense forest; **Record Level:** institutionID: Herbarium Togoense; collectionID: Abotsi, K.E.; institutionCode: TOGO; basisOfRecord: Preserved specimen

##### Ecological interactions

###### Native status

Native

##### Distribution

Zones 2 and 4

#### Parapolystichum barterianum

(Hook.) Rouhan

##### Materials

- **Type status:** Other material. **Occurrence:** catalogNumber: 12134; recordNumber: 5758; recordedBy: Brunel, J.-F.; **Taxon:** scientificName: Parapolystichum barterianum (Hook.) Rouhan; namePublishedIn: Brittonia 67(1): 82 (2014); kingdom: Plantae; phylum: Pteridophyta; class: Polypodiopsida; order: Polypodiales; family: Dryopteridaceae; genus: Parapolystichum; specificEpithet: barterianum; scientificNameAuthorship: (Hook.) Rouhan; **Location:** continent: Africa; country: Togo; countryCode: TG; locality: Kouma; verbatimElevation: 600; verbatimSRS: WGS85; decimalLatitude: 6.939; decimalLongitude: 0.6091; geodeticDatum: WGS85; **Identification:** identifiedBy: Meyer, C.A.; dateIdentified: 1980; **Event:** eventDate: /12/1978; habitat: Rainforest; **Record Level:** institutionID: Herbarium Togoense; collectionID: Brunel, J.-F.; institutionCode: TOGO; basisOfRecord: Preserved specimen

##### Ecological interactions

###### Native status

Native

##### Distribution

Zone 4

#### Parapolystichum subsimile

(Hook.) Rouhan

##### Materials

- **Type status:** Other material. **Occurrence:** catalogNumber: 12154; recordNumber: 29; recordedBy: Akpagana, K.; **Taxon:** scientificName: Parapolystichum subsimile (Hook.) Rouhan; namePublishedIn: Brittonia 67(1): 84 (2014); kingdom: Plantae; phylum: Pteridophyta; class: Polypodiopsida; order: Polypodiales; family: Dryopteridaceae; genus: Parapolystichum; specificEpithet: subsimile; scientificNameAuthorship: (Hook.) Rouhan; **Location:** continent: Africa; country: Togo; countryCode: TG; locality: Adamé; verbatimElevation: 610; verbatimSRS: WGS84; decimalLatitude: 6.35; decimalLongitude: 1.2227778; geodeticDatum: WGS84; **Identification:** identifiedBy: K. Akpagana; dateIdentified: 09-01-82; **Event:** eventDate: 09-01-82; habitat: Rainforest; **Record Level:** institutionID: Herbarium Togoense; collectionID: Akpagana, K.; institutionCode: TOGO; basisOfRecord: Preserved specimen
- **Type status:** Other material. **Occurrence:** catalogNumber: 12155; recordNumber: 5589; recordedBy: Brunel, J.-F.; **Taxon:** scientificName: Parapolystichum subsimile (Hook.) Rouhan; namePublishedIn: Brittonia 67(1): 84 (2014); kingdom: Plantae; phylum: Pteridophyta; class: Polypodiopsida; order: Polypodiales; family: Dryopteridaceae; genus: Parapolystichum; specificEpithet: subsimile; scientificNameAuthorship: (Hook.) Rouhan; **Location:** continent: Africa; country: Togo; countryCode: TG; locality: Yoh; verbatimElevation: 300; verbatimSRS: WGS84; decimalLatitude: 7; decimalLongitude: 0.75; geodeticDatum: WGS84; **Identification:** identifiedBy: Meyer, C.A.; dateIdentified: 1980; **Event:** eventDate: /10/1978; habitat: Rainforest; **Record Level:** institutionID: Herbarium Togoense; collectionID: Brunel, J.-F.; institutionCode: TOGO; basisOfRecord: Preserved specimen

##### Ecological interactions

###### Native status

Native

##### Distribution

Zone 4

#### Lomariopsidaceae

#### Lomariopsis guineensis

(Underw.) Alston

##### Materials

- **Type status:** Other material. **Occurrence:** recordNumber: AB0419; recordedBy: Abotsi, K.E.; **Taxon:** scientificName: Lomariopsis guineensis (Underw.) Alston; namePublishedIn: J. Bot. 72 (Suppl.): 5 (1934); kingdom: Plantae; phylum: Pteridophyta; class: Polypodiopsida; order: Polypodiales; family: Lomariopsidaceae; genus: Lomariopsis; specificEpithet: guineensis; scientificNameAuthorship: (Underw.) Alston; **Location:** continent: Africa; country: Togo; countryCode: TG; locality: Danyi Dzogbégan; verbatimElevation: 714; verbatimSRS: WGS84; decimalLatitude: 7.232082; decimalLongitude: 0.677545; geodeticDatum: WGS84; **Identification:** identifiedBy: Abotsi, K.E.; dateIdentified: 12-27-16; **Event:** eventDate: 12-27-16; habitat: Rainforest; **Record Level:** institutionID: Herbarium Togoense; collectionID: Abotsi, K.E.; institutionCode: TOGO; basisOfRecord: Preserved specimen
- **Type status:** Other material. **Occurrence:** recordNumber: ASM 0151; recordedBy: Abotsi, K.E., Sodjinou E. & Mingou P.; **Taxon:** scientificName: Lomariopsis guineensis (Underw.) Alston; namePublishedIn: J. Bot. 72 (Suppl.): 5 (1934); kingdom: Plantae; phylum: Pteridophyta; class: Polypodiopsida; order: Polypodiales; family: Lomariopsidaceae; genus: Lomariopsis; specificEpithet: guineensis; scientificNameAuthorship: (Underw.) Alston; **Location:** continent: Africa; country: Togo; countryCode: TG; locality: Dikpéléou; verbatimElevation: 696; verbatimSRS: WGS84; decimalLatitude: 8.195824911; decimalLongitude: 0.614532118; geodeticDatum: WGS84; **Identification:** identifiedBy: Abotsi, K.E.; dateIdentified: 05-09-13; **Event:** eventDate: 05-09-13; habitat: Rainforest; **Record Level:** institutionID: Herbarium Togoense; collectionID: Abotsi, K.E.; institutionCode: TOGO; basisOfRecord: Preserved specimen

##### Ecological interactions

###### Native status

Native

##### Distribution

Zone 4

#### Nephrolepidaceae

#### Nephrolepis bisserata

(Sw.) Schott.

##### Materials

- **Type status:** Other material. **Occurrence:** recordNumber: AB0202; recordedBy: Abotsi, K.E.; **Taxon:** scientificName: Nephrolepis bisserata (Sw.) Schott.; namePublishedIn: Gen. Fil. ad., t. 3 (1834); kingdom: Plantae; phylum: Pteridophyta; class: Polypodiopsida; order: Polypodiales; family: Nephrolepidaceae; genus: Nephrolepis; specificEpithet: bisserata; scientificNameAuthorship: (Sw.) Schott.; **Location:** continent: Africa; country: Togo; countryCode: TG; locality: Kpimé Séva; verbatimElevation: 523; verbatimSRS: WGS84; decimalLatitude: 7.010523; decimalLongitude: 0.639052; geodeticDatum: WGS84; **Identification:** identifiedBy: Abotsi, K.E.; dateIdentified: 08-04-16; **Event:** eventDate: 08-04-16; habitat: Palm grove; **Record Level:** institutionID: Herbarium Togoense; collectionID: Abotsi, K.E.; institutionCode: TOGO; basisOfRecord: Preserved specimen
- **Type status:** Other material. **Occurrence:** recordNumber: AB0330; recordedBy: Abotsi, K.E.; **Taxon:** scientificName: Nephrolepis bisserata (Sw.) Schott.; namePublishedIn: Gen. Fil. ad., t. 3 (1834); kingdom: Plantae; phylum: Pteridophyta; class: Polypodiopsida; order: Polypodiales; family: Nephrolepidaceae; genus: Nephrolepis; specificEpithet: bisserata; scientificNameAuthorship: (Sw.) Schott.; **Location:** continent: Africa; country: Togo; countryCode: TG; locality: Assoukoko; verbatimElevation: 336; verbatimSRS: WGS84; decimalLatitude: 8.005672; decimalLongitude: 0.61372; geodeticDatum: WGS84; **Identification:** identifiedBy: Abotsi, K.E.; dateIdentified: 09-18-16; **Event:** eventDate: 09-18-16; habitat: Fallow; **Record Level:** institutionID: Herbarium Togoense; collectionID: Abotsi, K.E.; institutionCode: TOGO; basisOfRecord: Preserved specimen
- **Type status:** Other material. **Occurrence:** recordNumber: AB0364; recordedBy: Abotsi, K.E.; **Taxon:** scientificName: Nephrolepis bisserata (Sw.) Schott.; namePublishedIn: Gen. Fil. ad., t. 3 (1834); kingdom: Plantae; phylum: Pteridophyta; class: Polypodiopsida; order: Polypodiales; family: Nephrolepidaceae; genus: Nephrolepis; specificEpithet: bisserata; scientificNameAuthorship: (Sw.) Schott.; **Location:** continent: Africa; country: Togo; countryCode: TG; locality: M'Poti; verbatimElevation: 643; verbatimSRS: WGS84; decimalLatitude: 8.243432; decimalLongitude: 0.775189; geodeticDatum: WGS84; **Identification:** identifiedBy: Abotsi, K.E.; dateIdentified: 10-05-16; **Event:** eventDate: 10-05-16; habitat: Rainforest; **Record Level:** institutionID: Herbarium Togoense; collectionID: Abotsi, K.E.; institutionCode: TOGO; basisOfRecord: Preserved specimen

##### Ecological interactions

###### Native status

Native

##### Distribution

Zones 2, 3, 4 and 5

#### Nephrolepis cordifolia

(L.) Presl

##### Materials

- **Type status:** Other material. **Occurrence:** recordNumber: AB0388; recordedBy: Abotsi, K.E.; **Taxon:** scientificName: Nephrolepis cordifolia (L.) Presl; namePublishedIn: Tent. Pterid. 79 (1836), nom. cons.; kingdom: Plantae; phylum: Pteridophyta; class: Polypodiopsida; order: Polypodiales; family: Nephrolepidaceae; genus: Nephrolepis; specificEpithet: cordifolia; scientificNameAuthorship: (L.) Presl; **Location:** continent: Africa; country: Togo; countryCode: TG; locality: Diguengué; verbatimElevation: 437; verbatimSRS: WGS84; decimalLatitude: 8.073798; decimalLongitude: 0.640721; geodeticDatum: WGS84; **Identification:** identifiedBy: Abotsi, K.E.; dateIdentified: 10-06-16; **Event:** eventDate: 10-06-16; habitat: Fallow; **Record Level:** institutionID: Herbarium Togoense; collectionID: Abotsi, K.E.; institutionCode: TOGO; basisOfRecord: Preserved specimen
- **Type status:** Other material. **Occurrence:** recordNumber: AB0547; recordedBy: Abotsi, K.E.; **Taxon:** scientificName: Nephrolepis cordifolia (L.) Presl; namePublishedIn: Tent. Pterid. 79 (1836), nom. cons.; kingdom: Plantae; phylum: Pteridophyta; class: Polypodiopsida; order: Polypodiales; family: Nephrolepidaceae; genus: Nephrolepis; specificEpithet: cordifolia; scientificNameAuthorship: (L.) Presl; **Location:** continent: Africa; country: Togo; countryCode: TG; locality: Koumondè; verbatimElevation: 696; verbatimSRS: WGS84; decimalLatitude: 9.275811; decimalLongitude: 1.223044; geodeticDatum: WGS84; **Identification:** identifiedBy: Abotsi, K.E.; dateIdentified: 07-12-17; **Event:** eventDate: 07-12-17; habitat: Wooded savannah; **Record Level:** institutionID: Herbarium Togoense; collectionID: Abotsi, K.E.; institutionCode: TOGO; basisOfRecord: Preserved specimen
- **Type status:** Other material. **Occurrence:** recordNumber: AB0550; recordedBy: Abotsi, K.E.; **Taxon:** scientificName: Nephrolepis cordifolia (L.) Presl; namePublishedIn: Tent. Pterid. 79 (1836), nom. cons.; kingdom: Plantae; phylum: Pteridophyta; class: Polypodiopsida; order: Polypodiales; family: Nephrolepidaceae; genus: Nephrolepis; specificEpithet: cordifolia; scientificNameAuthorship: (L.) Presl; **Location:** continent: Africa; country: Togo; countryCode: TG; locality: Alédjo; verbatimElevation: 517; verbatimSRS: WGS84; decimalLatitude: 9.272262; decimalLongitude: 1.207198; geodeticDatum: WGS84; **Identification:** identifiedBy: Abotsi, K.E.; dateIdentified: 07-12-17; **Event:** eventDate: 07-12-17; habitat: Dry dense forest; **Record Level:** institutionID: Herbarium Togoense; collectionID: Abotsi, K.E.; institutionCode: TOGO; basisOfRecord: Preserved specimen

##### Ecological interactions

###### Native status

Native

##### Distribution

Zones 2 and 4

#### Nephrolepis delicatula

(Decne.) Pic. Serm.

##### Materials

- **Type status:** Other material. **Occurrence:** recordNumber: AB0272; recordedBy: Abotsi, K.E.; **Taxon:** scientificName: Nephrolepis delicatula (Decne.) Pic. Serm.; namePublishedIn: Webbia 23: 181 (1968); kingdom: Plantae; phylum: Pteridophyta; class: Polypodiopsida; order: Polypodiales; family: Nephrolepidaceae; genus: Nephrolepis; specificEpithet: delicatula; scientificNameAuthorship: (Decne.) Pic. Serm.; **Location:** continent: Africa; country: Togo; countryCode: TG; locality: Mouna; verbatimElevation: 558; verbatimSRS: WGS84; decimalLatitude: 7.642163; decimalLongitude: 0.873798; geodeticDatum: WGS84; **Identification:** identifiedBy: Abotsi, K.E.; dateIdentified: 09-15-16; **Event:** eventDate: 09-15-16; habitat: Rainforest; **Record Level:** institutionID: Herbarium Togoense; collectionID: Abotsi, K.E.; institutionCode: TOGO; basisOfRecord: Preserved specimen
- **Type status:** Other material. **Occurrence:** recordNumber: AB0538; recordedBy: Abotsi, K.E.; **Taxon:** scientificName: Nephrolepis delicatula (Decne.) Pic. Serm.; namePublishedIn: Webbia 23: 181 (1968); kingdom: Plantae; phylum: Pteridophyta; class: Polypodiopsida; order: Polypodiales; family: Nephrolepidaceae; genus: Nephrolepis; specificEpithet: delicatula; scientificNameAuthorship: (Decne.) Pic. Serm.; **Location:** continent: Africa; country: Togo; countryCode: TG; locality: Défalé; verbatimElevation: 293; verbatimSRS: WGS84; decimalLatitude: 9.924749; decimalLongitude: 1.108493; geodeticDatum: WGS84; **Identification:** identifiedBy: Abotsi, K.E.; dateIdentified: 07-11-17; **Event:** eventDate: 07-11-17; habitat: Dry dense forest; **Record Level:** institutionID: Herbarium Togoense; collectionID: Abotsi, K.E.; institutionCode: TOGO; basisOfRecord: Preserved specimen
- **Type status:** Other material. **Occurrence:** recordNumber: AB0568; recordedBy: Abotsi, K.E.; **Taxon:** scientificName: Nephrolepis delicatula (Decne.) Pic. Serm.; namePublishedIn: Webbia 23: 181 (1968); kingdom: Plantae; phylum: Pteridophyta; class: Polypodiopsida; order: Polypodiales; family: Nephrolepidaceae; genus: Nephrolepis; specificEpithet: delicatula; scientificNameAuthorship: (Decne.) Pic. Serm.; **Location:** continent: Africa; country: Togo; countryCode: TG; locality: Tchatchaminadè; verbatimElevation: 397; verbatimSRS: WGS84; decimalLatitude: 9.310474; decimalLongitude: 0.984666; geodeticDatum: WGS84; **Identification:** identifiedBy: Abotsi, K.E.; dateIdentified: 07-14-17; **Event:** eventDate: 07-14-17; habitat: Dry dense forest; **Record Level:** institutionID: Herbarium Togoense; collectionID: Abotsi, K.E.; institutionCode: TOGO; basisOfRecord: Preserved specimen

##### Ecological interactions

###### Native status

Native

##### Distribution

Zones 2 and 4

#### Nephrolepis hirsutula

(G. Forst.) Presl

##### Materials

- **Type status:** Other material. **Occurrence:** recordedBy: Abotsi, K.E.; **Taxon:** scientificName: Nephrolepis hirsutula (G. Forst.) Presl; namePublishedIn: Tent. Pterid. 79 (1836); kingdom: Plantae; phylum: Pteridophyta; class: Polypodiopsida; order: Polypodiales; family: Nephrolepidaceae; genus: Nephrolepis; specificEpithet: hirsutula; scientificNameAuthorship: (G. Forst.) Presl; **Location:** continent: Africa; country: Togo; countryCode: TG; locality: Lomé, in gardens; **Identification:** identifiedBy: Abotsi, K.E.; **Event:** habitat: Gardens; **Record Level:** basisOfRecord: Human observation

##### Ecological interactions

###### Native status

Not native

##### Distribution

In gardens

#### Nephrolepis undulata

(Afzel. ex Sw.) J. Sm.

##### Materials

- **Type status:** Other material. **Occurrence:** recordNumber: AB0078; recordedBy: Abotsi, K.E.; **Taxon:** scientificName: Nephrolepis undulata (Afzel. ex Sw.) J. Sm.; namePublishedIn: Bot. Mag. 72 (Companion): 35 bis (1846); kingdom: Plantae; phylum: Pteridophyta; class: Polypodiopsida; order: Polypodiales; family: Nephrolepidaceae; genus: Nephrolepis; specificEpithet: undulata; scientificNameAuthorship: (Afzel. ex Sw.) J. Sm.; **Location:** continent: Africa; country: Togo; countryCode: TG; locality: Kuma Konda; verbatimElevation: 567; verbatimSRS: WGS84; decimalLatitude: 6.949813; decimalLongitude: 0.57751; geodeticDatum: WGS84; **Identification:** identifiedBy: Abotsi, K.E.; dateIdentified: 06-24-16; **Event:** eventDate: 06-24-16; habitat: Rainforest; **Record Level:** institutionID: Herbarium Togoense; collectionID: Abotsi, K.E.; institutionCode: TOGO; basisOfRecord: Preserved specimen
- **Type status:** Other material. **Occurrence:** recordNumber: AB0178; recordedBy: Abotsi, K.E.; **Taxon:** scientificName: Nephrolepis undulata (Afzel. ex Sw.) J. Sm.; namePublishedIn: Bot. Mag. 72 (Companion): 35 bis (1846); kingdom: Plantae; phylum: Pteridophyta; class: Polypodiopsida; order: Polypodiales; family: Nephrolepidaceae; genus: Nephrolepis; specificEpithet: undulata; scientificNameAuthorship: (Afzel. ex Sw.) J. Sm.; **Location:** continent: Africa; country: Togo; countryCode: TG; locality: Womé; verbatimElevation: 359; verbatimSRS: WGS84; decimalLatitude: 6.858394; decimalLongitude: 0.556622; geodeticDatum: WGS84; **Identification:** identifiedBy: Abotsi, K.E.; dateIdentified: 08-03-16; **Event:** eventDate: 08-03-16; habitat: Palm grove; **Record Level:** institutionID: Herbarium Togoense; collectionID: Abotsi, K.E.; institutionCode: TOGO; basisOfRecord: Preserved specimen
- **Type status:** Other material. **Occurrence:** recordNumber: AB0278; recordedBy: Abotsi, K.E.; **Taxon:** scientificName: Nephrolepis undulata (Afzel. ex Sw.) J. Sm.; namePublishedIn: Bot. Mag. 72 (Companion): 35 bis (1846); kingdom: Plantae; phylum: Pteridophyta; class: Polypodiopsida; order: Polypodiales; family: Nephrolepidaceae; genus: Nephrolepis; specificEpithet: undulata; scientificNameAuthorship: (Afzel. ex Sw.) J. Sm.; **Location:** continent: Africa; country: Togo; countryCode: TG; locality: Vhè N'Kugna; verbatimElevation: 541; verbatimSRS: WGS84; decimalLatitude: 7.660917; decimalLongitude: 0.748113; geodeticDatum: WGS84; **Identification:** identifiedBy: Abotsi, K.E.; dateIdentified: 09-16-16; **Event:** eventDate: 09-16-16; habitat: Wooded savannah; **Record Level:** institutionID: Herbarium Togoense; collectionID: Abotsi, K.E.; institutionCode: TOGO; basisOfRecord: Preserved specimen

##### Ecological interactions

###### Native status

Native

##### Distribution

Zones 2, 3 and 4

#### Oleandraceae

#### Oleandra distenta

Kunze

##### Materials

- **Type status:** Other material. **Occurrence:** recordedBy: Roux, J.P.; **Taxon:** scientificName: Oleandra distenta Kunze; namePublishedIn: Bot. Zeit. (Berlin) 347 (1851); kingdom: Plantae; phylum: Pteridophyta; class: Polypodiopsida; order: Polypodiales; family: Oleandraceae; genus: Oleandra; specificEpithet: distenta; scientificNameAuthorship: Kunze; **Location:** continent: Africa; country: Togo; countryCode: TG; **Event:** habitat: Rainforest; **Record Level:** basisOfRecord: Unknown; source: Synopsis of the Lycopodiophyta and Pteridophyta of Africa, Madagascar and neighbouring islands (Roux, 2009)

##### Ecological interactions

###### Native status

Native

##### Distribution

Zone 4

#### Polypodiaceae

#### Drynarioideae

#### Aglaomorpha laurentii

(Christ ex De Wild. & Durand) Hovenkamp & S.Linds.

##### Materials

- **Type status:** Other material. **Occurrence:** catalogNumber: 12318; recordNumber: 1848; recordedBy: Akpagana, K.; **Taxon:** scientificName: Aglaomorpha laurentii (Christ ex De Wild. & Durand) Hovenkamp & S.Linds.; namePublishedIn: Gard. Bull. Singapore 69(1): 151 (2017); kingdom: Plantae; phylum: Pteridophyta; class: Polypodiopsida; order: Polypodiales; family: Polypodiaceae; genus: Aglaomorpha; specificEpithet: laurentii; scientificNameAuthorship: (Christ ex De Wild. & Durand) Hovenkamp & S.Linds.; **Location:** continent: Africa; country: Togo; countryCode: TG; locality: Danyi Elavagnon; verbatimElevation: 769; verbatimSRS: WGS84; decimalLatitude: 7.2904; decimalLongitude: 0.707708; geodeticDatum: WGS84; **Identification:** identifiedBy: K. Akpagana; dateIdentified: /07/1987; **Event:** eventDate: /07/1987; habitat: Rainforest; **Record Level:** institutionID: Herbarium Togoense; collectionID: Akpagana, K.; institutionCode: TOGO; basisOfRecord: Preserved specimen
- **Type status:** Other material. **Occurrence:** catalogNumber: 12317; recordNumber: 7863; recordedBy: Brunel, J.-F.; **Taxon:** scientificName: Aglaomorpha laurentii (Christ ex De Wild. & Durand) Hovenkamp & S.Linds.; namePublishedIn: Gard. Bull. Singapore 69(1): 151 (2017); kingdom: Plantae; phylum: Pteridophyta; class: Polypodiopsida; order: Polypodiales; family: Polypodiaceae; genus: Aglaomorpha; specificEpithet: laurentii; scientificNameAuthorship: (Christ ex De Wild. & Durand) Hovenkamp & S.Linds.; **Location:** continent: Africa; country: Togo; countryCode: TG; locality: Danyi Dzogbégan; verbatimElevation: 714; verbatimSRS: WGS84; decimalLatitude: 7.232082; decimalLongitude: 0.677545; geodeticDatum: WGS84; **Identification:** identifiedBy: Brunel, J.-F.; dateIdentified: /06/1983; **Event:** eventDate: /06/1983; habitat: Rainforest; **Record Level:** institutionID: Herbarium Togoense; collectionID: Brunel, J.-F.; institutionCode: TOGO; basisOfRecord: Preserved specimen

##### Ecological interactions

###### Native status

Native

##### Distribution

Zone 4

#### Grammitidoideae

#### Cochlidium punctatum

(Raddi) L.E. Bishop

##### Materials

- **Type status:** Other material. **Occurrence:** recordedBy: Abotsi, K.E.; **Taxon:** scientificName: Cochlidium punctatum (Raddi) L.E. Bishop; namePublishedIn: Amer. Fern J. 68(3): 86 (1978); kingdom: Plantae; phylum: Pteridophyta; class: Polypodiopsida; order: Polypodiales; family: Polypodiaceae; genus: Cochlidium; specificEpithet: punctatum; scientificNameAuthorship: (Raddi) L.E. Bishop; **Location:** continent: Africa; country: Togo; countryCode: TG; locality: Lomé, in gardens; **Identification:** identifiedBy: Abotsi, K.E.; **Event:** habitat: Gardens; **Record Level:** basisOfRecord: Human observation

##### Ecological interactions

###### Native status

Not native

##### Distribution

In gardens

#### Loxogrammoideae

#### Loxogramme abyssinica

(Baker) M. G. Price

##### Materials

- **Type status:** Other material. **Occurrence:** recordNumber: AB0113; recordedBy: Abotsi, K.E.; **Taxon:** scientificName: Loxogramme abyssinica (Baker) M. G. Price; namePublishedIn: Amer. Fern J. 74(2): 61 (1984); kingdom: Plantae; phylum: Pteridophyta; class: Polypodiopsida; order: Polypodiales; family: Polypodiaceae; genus: Loxogramme; specificEpithet: abyssinica; scientificNameAuthorship: (Baker) M. G. Price; **Location:** continent: Africa; country: Togo; countryCode: TG; locality: Danyi N'Digbé; verbatimElevation: 675; verbatimSRS: WGS84; decimalLatitude: 7.153184; decimalLongitude: 0.666966; geodeticDatum: WGS84; **Identification:** identifiedBy: Abotsi, K.E.; dateIdentified: 06-27-16; **Event:** eventDate: 06-27-16; habitat: Rainforest; **Record Level:** institutionID: Herbarium Togoense; collectionID: Abotsi, K.E.; institutionCode: TOGO; basisOfRecord: Preserved specimen
- **Type status:** Other material. **Occurrence:** recordNumber: AB0292; recordedBy: Abotsi, K.E.; **Taxon:** scientificName: Loxogramme abyssinica (Baker) M. G. Price; namePublishedIn: Amer. Fern J. 74(2): 61 (1984); kingdom: Plantae; phylum: Pteridophyta; class: Polypodiopsida; order: Polypodiales; family: Polypodiaceae; genus: Loxogramme; specificEpithet: abyssinica; scientificNameAuthorship: (Baker) M. G. Price; **Location:** continent: Africa; country: Togo; countryCode: TG; locality: Sérégbéné; verbatimElevation: 524; verbatimSRS: WGS84; decimalLatitude: 7.865006; decimalLongitude: 0.739799; geodeticDatum: WGS84; **Identification:** identifiedBy: Abotsi, K.E.; dateIdentified: 09-17-16; **Event:** eventDate: 09-17-16; habitat: Rainforest; **Record Level:** institutionID: Herbarium Togoense; collectionID: Abotsi, K.E.; institutionCode: TOGO; basisOfRecord: Preserved specimen
- **Type status:** Other material. **Occurrence:** recordNumber: AB0305; recordedBy: Abotsi, K.E.; **Taxon:** scientificName: Loxogramme abyssinica (Baker) M. G. Price; namePublishedIn: Amer. Fern J. 74(2): 61 (1984); kingdom: Plantae; phylum: Pteridophyta; class: Polypodiopsida; order: Polypodiales; family: Polypodiaceae; genus: Loxogramme; specificEpithet: abyssinica; scientificNameAuthorship: (Baker) M. G. Price; **Location:** continent: Africa; country: Togo; countryCode: TG; locality: Aménosodzi; verbatimElevation: 259; verbatimSRS: WGS84; decimalLatitude: 7.903039; decimalLongitude: 0.650732; geodeticDatum: WGS84; **Identification:** identifiedBy: Abotsi, K.E.; dateIdentified: 09-17-16; **Event:** eventDate: 09-17-16; habitat: Rainforest; **Record Level:** institutionID: Herbarium Togoense; collectionID: Abotsi, K.E.; institutionCode: TOGO; basisOfRecord: Preserved specimen

##### Ecological interactions

###### Native status

Native

##### Distribution

Zone 4

#### Microsoroideae

#### Lepisorus excavatus

(Bory ex Willd.) Ching

##### Materials

- **Type status:** Other material. **Occurrence:** recordNumber: ASM 0267; recordedBy: Abotsi, K.E.; **Taxon:** scientificName: Lepisorus excavatus (Bory ex Willd.) Ching; namePublishedIn: Bull. Fan Mem. Inst. Biol. Bot. 4: 68 (1939); kingdom: Plantae; phylum: Pteridophyta; class: Polypodiopsida; order: Polypodiales; family: Polypodiaceae; genus: Lepisorus; specificEpithet: excavatus; scientificNameAuthorship: (Bory ex Willd.) Ching; **Location:** continent: Africa; country: Togo; countryCode: TG; locality: Danyi Dzogbégan; verbatimElevation: 753; verbatimSRS: WGS84; decimalLatitude: 7.234158085; decimalLongitude: 0.71352374; geodeticDatum: WGS84; **Identification:** identifiedBy: Abotsi, K.E.; dateIdentified: 04-10-13; **Event:** eventDate: 04-10-13; habitat: Rainforest; **Record Level:** institutionID: Herbarium Togoense; collectionID: Abotsi, K.E.; institutionCode: TOGO; basisOfRecord: Preserved specimen
- **Type status:** Other material. **Occurrence:** recordNumber: ASM 0328; recordedBy: Abotsi, K.E.; **Taxon:** scientificName: Lepisorus excavatus (Bory ex Willd.) Ching; namePublishedIn: Bull. Fan Mem. Inst. Biol. Bot. 4: 68 (1939); kingdom: Plantae; phylum: Pteridophyta; class: Polypodiopsida; order: Polypodiales; family: Polypodiaceae; genus: Lepisorus; specificEpithet: excavatus; scientificNameAuthorship: (Bory ex Willd.) Ching; **Location:** continent: Africa; country: Togo; countryCode: TG; locality: Mt Agou; verbatimElevation: 982; verbatimSRS: WGS84; decimalLatitude: 6.872570656; decimalLongitude: 0.748960964; geodeticDatum: WGS84; **Identification:** identifiedBy: Abotsi, K.E.; dateIdentified: 04-16-13; **Event:** eventDate: 04-16-13; habitat: Rainforest; **Record Level:** institutionID: Herbarium Togoense; collectionID: Abotsi, K.E.; institutionCode: TOGO; basisOfRecord: Preserved specimen

##### Ecological interactions

###### Native status

Native

##### Distribution

Zones 2 and 4

#### Microsorum musifolium

(Blume) Copel.

##### Materials

- **Type status:** Other material. **Occurrence:** recordedBy: Abotsi, K.E.; **Taxon:** scientificName: Microsorum musifolium (Blume) Copel.; namePublishedIn: Univ. Calif. Publ. Bot. 16: 111-116 (1929); kingdom: Plantae; phylum: Pteridophyta; class: Polypodiopsida; order: Polypodiales; family: Polypodiaceae; genus: Microsorum; specificEpithet: musifolium; scientificNameAuthorship: (Blume) Copel.; **Location:** continent: Africa; country: Togo; countryCode: TG; locality: Lomé, in gardens; **Identification:** identifiedBy: Abotsi, K.E.; **Event:** habitat: Gardens; **Record Level:** basisOfRecord: Human observation

##### Ecological interactions

###### Native status

Not native

##### Distribution

In gardens

#### Microsorum punctatum

(L.) Copel.

##### Materials

- **Type status:** Other material. **Occurrence:** recordNumber: AB0109; recordedBy: Abotsi, K.E.; **Taxon:** scientificName: Microsorum punctatum (L.) Copel.; namePublishedIn: Univ. Calif. Publ. Bot. 16: 110 (1929); kingdom: Plantae; phylum: Pteridophyta; class: Polypodiopsida; order: Polypodiales; family: Polypodiaceae; genus: Microsorum; specificEpithet: punctatum; scientificNameAuthorship: (L.) Copel.; **Location:** continent: Africa; country: Togo; countryCode: TG; locality: Danyi N'Digbé; verbatimElevation: 685; verbatimSRS: WGS84; decimalLatitude: 7.151191; decimalLongitude: 0.671993; geodeticDatum: WGS84; **Identification:** identifiedBy: Abotsi, K.E.; dateIdentified: 06-27-16; **Event:** eventDate: 06-27-16; habitat: Rainforest; **Record Level:** institutionID: Herbarium Togoense; collectionID: Abotsi, K.E.; institutionCode: TOGO; basisOfRecord: Preserved specimen
- **Type status:** Other material. **Occurrence:** recordNumber: AB0413; recordedBy: Abotsi, K.E.; **Taxon:** scientificName: Microsorum punctatum (L.) Copel.; namePublishedIn: Univ. Calif. Publ. Bot. 16: 110 (1929); kingdom: Plantae; phylum: Pteridophyta; class: Polypodiopsida; order: Polypodiales; family: Polypodiaceae; genus: Microsorum; specificEpithet: punctatum; scientificNameAuthorship: (L.) Copel.; **Location:** continent: Africa; country: Togo; countryCode: TG; locality: Danyi Dzogbégan; verbatimElevation: 711; verbatimSRS: WGS84; decimalLatitude: 7.232356; decimalLongitude: 0.677425; geodeticDatum: WGS84; **Identification:** identifiedBy: Abotsi, K.E.; dateIdentified: 12-27-16; **Event:** eventDate: 12-27-16; habitat: Rainforest; **Record Level:** institutionID: Herbarium Togoense; collectionID: Abotsi, K.E.; institutionCode: TOGO; basisOfRecord: Preserved specimen
- **Type status:** Other material. **Occurrence:** recordNumber: AB0479; recordedBy: Abotsi, K.E.; **Taxon:** scientificName: Microsorum punctatum (L.) Copel.; namePublishedIn: Univ. Calif. Publ. Bot. 16: 110 (1929); kingdom: Plantae; phylum: Pteridophyta; class: Polypodiopsida; order: Polypodiales; family: Polypodiaceae; genus: Microsorum; specificEpithet: punctatum; scientificNameAuthorship: (L.) Copel.; **Location:** continent: Africa; country: Togo; countryCode: TG; locality: Ediwlou; verbatimElevation: 582; verbatimSRS: WGS84; decimalLatitude: 7.404205; decimalLongitude: 0.700612; geodeticDatum: WGS84; **Identification:** identifiedBy: Abotsi, K.E.; dateIdentified: 04-25-17; **Event:** eventDate: 04-25-17; habitat: Rainforest; **Record Level:** institutionID: Herbarium Togoense; collectionID: Abotsi, K.E.; institutionCode: TOGO; basisOfRecord: Preserved specimen

##### Ecological interactions

###### Native status

Native

##### Distribution

Zone 4

#### Microsorum scolopendria

(Burm.f.) Copel.

##### Materials

- **Type status:** Other material. **Occurrence:** recordNumber: AB0533; recordedBy: Abotsi, K.E.; **Taxon:** scientificName: Microsorum scolopendria (Burm.f.) Copel.; namePublishedIn: Univ. Calif. Publ. Bot. 16. 112. (1929); kingdom: Plantae; phylum: Pteridophyta; class: Polypodiopsida; order: Polypodiales; family: Polypodiaceae; genus: Microsorum; specificEpithet: scolopendria; scientificNameAuthorship: (Burm. f.) Copel.; **Location:** continent: Africa; country: Togo; countryCode: TG; locality: Afito; verbatimElevation: 27; verbatimSRS: WGS84; decimalLatitude: 6.753295; decimalLongitude: 1.5874880555556; geodeticDatum: WGS84; **Identification:** identifiedBy: Abotsi, K.E.; dateIdentified: 06-29-17; **Event:** eventDate: 06-29-17; habitat: Palm grove; **Record Level:** institutionID: Herbarium Togoense; collectionID: Abotsi, K.E.; institutionCode: TOGO; basisOfRecord: Preserved specimen
- **Type status:** Other material. **Occurrence:** recordNumber: AB0084; recordedBy: Abotsi, K.E.; **Taxon:** scientificName: Microsorum scolopendria (Burm.f.) Copel.; namePublishedIn: Univ. Calif. Publ. Bot. 16. 112. (1929); kingdom: Plantae; phylum: Pteridophyta; class: Polypodiopsida; order: Polypodiales; family: Polypodiaceae; genus: Microsorum; specificEpithet: scolopendria; scientificNameAuthorship: (Burm. f.) Copel.; **Location:** continent: Africa; country: Togo; countryCode: TG; locality: Kuma Konda; verbatimElevation: 570; verbatimSRS: WGS84; decimalLatitude: 6.948919; decimalLongitude: 0.578778; geodeticDatum: WGS84; **Identification:** identifiedBy: Abotsi, K.E.; dateIdentified: 06-24-16; **Event:** eventDate: 06-24-16; habitat: Rainforest; **Record Level:** institutionID: Herbarium Togoense; collectionID: Abotsi, K.E.; institutionCode: TOGO; basisOfRecord: Preserved specimen
- **Type status:** Other material. **Occurrence:** recordNumber: AB0249; recordedBy: Abotsi, K.E.; **Taxon:** scientificName: Microsorum scolopendria (Burm.f.) Copel.; namePublishedIn: Univ. Calif. Publ. Bot. 16. 112. (1929); kingdom: Plantae; phylum: Pteridophyta; class: Polypodiopsida; order: Polypodiales; family: Polypodiaceae; genus: Microsorum; specificEpithet: scolopendria; scientificNameAuthorship: (Burm. f.) Copel.; **Location:** continent: Africa; country: Togo; countryCode: TG; locality: Kessibo Abrewankor; verbatimElevation: 367; verbatimSRS: WGS84; decimalLatitude: 7.650175; decimalLongitude: 0.59982; geodeticDatum: WGS84; **Identification:** identifiedBy: Abotsi, K.E.; dateIdentified: 08-07-16; **Event:** eventDate: 08-07-16; habitat: Palm grove; **Record Level:** institutionID: Herbarium Togoense; collectionID: Abotsi, K.E.; institutionCode: TOGO; basisOfRecord: Preserved specimen

##### Ecological interactions

###### Native status

Native

##### Distribution

Zones 4 and 5

#### Platycerioideae

#### Platycerium bifurcatum

(Cav.) C. Chr.

##### Materials

- **Type status:** Other material. **Occurrence:** recordedBy: Abotsi, K.E.; **Taxon:** scientificName: Platycerium bifurcatum (Cav.) C. Chr.; namePublishedIn: Ind. 498 (1906); kingdom: Plantae; phylum: Pteridophyta; class: Polypodiopsida; order: Polypodiales; family: Polypodiaceae; genus: Platycerium; specificEpithet: bifurcatum; scientificNameAuthorship: (Cav.) C. Chr.; **Location:** continent: Africa; country: Togo; countryCode: TG; locality: Lomé, in gardens; **Identification:** identifiedBy: Abotsi, K.E.; **Event:** habitat: Gardens; **Record Level:** basisOfRecord: Human observation

##### Ecological interactions

###### Native status

Not native

##### Distribution

In gardens

#### Platycerium elephantotis

Schweinf.

##### Materials

- **Type status:** Other material. **Occurrence:** recordNumber: AB0551; recordedBy: Abotsi, K.E.; **Taxon:** scientificName: Platycerium elephantotis Schweinf.; namePublishedIn: Bot. Zeit. (Berlin) 361 (1871); kingdom: Plantae; phylum: Pteridophyta; class: Polypodiopsida; order: Polypodiales; family: Polypodiaceae; genus: Platycerium; specificEpithet: elephantotis; scientificNameAuthorship: Schweinf.; **Location:** continent: Africa; country: Togo; countryCode: TG; locality: Alédjo; verbatimElevation: 518; verbatimSRS: WGS84; decimalLatitude: 9.272384; decimalLongitude: 1.207383; geodeticDatum: WGS84; **Identification:** identifiedBy: Abotsi, K.E.; dateIdentified: 07-12-17; **Event:** eventDate: 07-12-17; habitat: Dry dense forest; **Record Level:** institutionID: Herbarium Togoense; collectionID: Abotsi, K.E.; institutionCode: TOGO; basisOfRecord: Preserved specimen
- **Type status:** Other material. **Occurrence:** recordNumber: AB0604; recordedBy: Abotsi, K.E.; **Taxon:** scientificName: Platycerium elephantotis Schweinf.; namePublishedIn: Bot. Zeit. (Berlin) 361 (1871); kingdom: Plantae; phylum: Pteridophyta; class: Polypodiopsida; order: Polypodiales; family: Polypodiaceae; genus: Platycerium; specificEpithet: elephantotis; scientificNameAuthorship: Schweinf.; **Location:** continent: Africa; country: Togo; countryCode: TG; locality: Diomé; verbatimElevation: 273; verbatimSRS: WGS84; decimalLatitude: 8.584624; decimalLongitude: 1.305894; geodeticDatum: WGS84; **Identification:** identifiedBy: Abotsi, K.E.; dateIdentified: 07-18-17; **Event:** eventDate: 07-18-17; habitat: Dry dense forest; **Record Level:** institutionID: Herbarium Togoense; collectionID: Abotsi, K.E.; institutionCode: TOGO; basisOfRecord: Preserved specimen
- **Type status:** Other material. **Occurrence:** recordNumber: AB0019; recordedBy: Abotsi, K.E.; **Taxon:** scientificName: Platycerium elephantotis Schweinf.; namePublishedIn: Bot. Zeit. (Berlin) 361 (1871); kingdom: Plantae; phylum: Pteridophyta; class: Polypodiopsida; order: Polypodiales; family: Polypodiaceae; genus: Platycerium; specificEpithet: elephantotis; scientificNameAuthorship: Schweinf.; **Location:** continent: Africa; country: Togo; countryCode: TG; locality: Alédjo Kadara; verbatimElevation: 777; verbatimSRS: WGS84; decimalLatitude: 9.257632; decimalLongitude: 1.210462; geodeticDatum: WGS84; **Identification:** identifiedBy: Abotsi, K.E.; dateIdentified: 07-13-17; **Event:** eventDate: 07-13-17; habitat: Inhabited area, on a tree; **Record Level:** institutionID: Herbarium Togoense; collectionID: Abotsi, K.E.; institutionCode: TOGO; basisOfRecord: Preserved specimen

##### Ecological interactions

###### Native status

Native

##### Distribution

Zone 2

#### Platycerium hillii

T. Moore

##### Materials

- **Type status:** Other material. **Occurrence:** recordedBy: Abotsi, K.E.; **Taxon:** scientificName: Platycerium hillii T. Moore; namePublishedIn: Gard. Chr. n. s. 10: 51, f. 6, 429, f. 74-75 (1878); kingdom: Plantae; phylum: Pteridophyta; class: Polypodiopsida; order: Polypodiales; family: Polypodiaceae; genus: Platycerium; specificEpithet: hillii; scientificNameAuthorship: T. Moore; **Location:** continent: Africa; country: Togo; countryCode: TG; locality: Lomé, in gardens; **Identification:** identifiedBy: Abotsi, K.E.; **Event:** habitat: Gardens; **Record Level:** basisOfRecord: Human observation

##### Ecological interactions

###### Native status

Not native

##### Distribution

In gardens

#### Platycerium stemaria

(P.Beauv.) Desv.

##### Materials

- **Type status:** Other material. **Occurrence:** recordNumber: AB0199; recordedBy: Abotsi, K.E.; **Taxon:** scientificName: Platycerium stemaria (P.Beauv.) Desv.; namePublishedIn: Mem. Soc. Linn. Paris 6, 2: 213 (1827) (stemmaria) ; Chr. 126. NPfl. 339; kingdom: Plantae; phylum: Pteridophyta; class: Polypodiopsida; order: Polypodiales; family: Polypodiaceae; genus: Platycerium; specificEpithet: stemaria; scientificNameAuthorship: (P.Beauv.) Desv.; **Location:** continent: Africa; country: Togo; countryCode: TG; locality: Kpimé Séva; verbatimElevation: 503; verbatimSRS: WGS84; decimalLatitude: 7.008194; decimalLongitude: 0.643401; geodeticDatum: WGS84; **Identification:** identifiedBy: Abotsi, K.E.; dateIdentified: 08-04-16; **Event:** eventDate: 08-04-16; habitat: Inhabited area, on a tree; **Record Level:** institutionID: Herbarium Togoense; collectionID: Abotsi, K.E.; institutionCode: TOGO; basisOfRecord: Preserved specimen
- **Type status:** Other material. **Occurrence:** recordNumber: AB0248; recordedBy: Abotsi, K.E.; **Taxon:** scientificName: Platycerium stemaria (P.Beauv.) Desv.; namePublishedIn: Mem. Soc. Linn. Paris 6, 2: 213 (1827) (stemmaria) ; Chr. 126. NPfl. 339; kingdom: Plantae; phylum: Pteridophyta; class: Polypodiopsida; order: Polypodiales; family: Polypodiaceae; genus: Platycerium; specificEpithet: stemaria; scientificNameAuthorship: (P.Beauv.) Desv.; **Location:** continent: Africa; country: Togo; countryCode: TG; locality: Kessibo Abrewankor; verbatimElevation: 369; verbatimSRS: WGS84; decimalLatitude: 7.650219; decimalLongitude: 0.599818; geodeticDatum: WGS84; **Identification:** identifiedBy: Abotsi, K.E.; dateIdentified: 08-07-16; **Event:** eventDate: 08-07-16; habitat: Palm grove; **Record Level:** institutionID: Herbarium Togoense; collectionID: Abotsi, K.E.; institutionCode: TOGO; basisOfRecord: Preserved specimen
- **Type status:** Other material. **Occurrence:** recordNumber: AB0338; recordedBy: Abotsi, K.E.; **Taxon:** scientificName: Platycerium stemaria (P.Beauv.) Desv.; namePublishedIn: Mem. Soc. Linn. Paris 6, 2: 213 (1827) (stemmaria) ; Chr. 126. NPfl. 339; kingdom: Plantae; phylum: Pteridophyta; class: Polypodiopsida; order: Polypodiales; family: Polypodiaceae; genus: Platycerium; specificEpithet: stemaria; scientificNameAuthorship: (P.Beauv.) Desv.; **Location:** continent: Africa; country: Togo; countryCode: TG; locality: Danyi Dzédramévi; verbatimElevation: 703; verbatimSRS: WGS84; decimalLatitude: 7.162683; decimalLongitude: 0.632576; geodeticDatum: WGS84; **Identification:** identifiedBy: Abotsi, K.E.; dateIdentified: 09-30-16; **Event:** eventDate: 09-30-16; habitat: Wooded savannah; **Record Level:** institutionID: Herbarium Togoense; collectionID: Abotsi, K.E.; institutionCode: TOGO; basisOfRecord: Preserved specimen

##### Ecological interactions

###### Native status

Native

##### Distribution

Zones 3, 4 and 5

#### Platycerium superbum

Joncheere & Hennipman

##### Materials

- **Type status:** Other material. **Occurrence:** recordedBy: Abotsi, K.E.; **Taxon:** scientificName: Platycerium superbum Joncheere & Hennipman; namePublishedIn: Tent. Pterid. 215, t. 9, f. 8 (1836)Brit. Fern Gaz. 10: 114, C 4, 5 (1970), nom. nov.; kingdom: Plantae; phylum: Pteridophyta; class: Polypodiopsida; order: Polypodiales; family: Polypodiaceae; genus: Platycerium; specificEpithet: superbum; scientificNameAuthorship: Joncheere & Hennipman; **Location:** continent: Africa; country: Togo; countryCode: TG; locality: Lomé, in gardens; **Identification:** identifiedBy: Abotsi, K.E.; **Event:** habitat: Gardens; **Record Level:** basisOfRecord: Human observation

##### Ecological interactions

###### Native status

Not native

##### Distribution

In gardens

#### Polypodioideae

#### Microgramma mauritiana

(Willd.) Tardieu

##### Materials

- **Type status:** Other material. **Occurrence:** recordNumber: AB0093; recordedBy: Abotsi, K.E.; **Taxon:** scientificName: Microgramma mauritiana (Willd.) Tardieu; namePublishedIn: Humbert, Fl. Madag. Fam. 5, 2: 108, t. 24(6-8) (1960); kingdom: Plantae; phylum: Pteridophyta; class: Polypodiopsida; order: Polypodiales; family: Polypodiaceae; genus: Microgramma; specificEpithet: mauritiana; scientificNameAuthorship: (Willd.) Tardieu; **Location:** continent: Africa; country: Togo; countryCode: TG; locality: Tové, INFA; verbatimElevation: 224; verbatimSRS: WGS84; decimalLatitude: 6.882528; decimalLongitude: 0.645221; geodeticDatum: WGS84; **Identification:** identifiedBy: Abotsi, K.E.; dateIdentified: 06-25-16; **Event:** eventDate: 06-25-16; habitat: Rainforest; **Record Level:** institutionID: Herbarium Togoense; collectionID: Abotsi, K.E.; institutionCode: TOGO; basisOfRecord: Preserved specimen
- **Type status:** Other material. **Occurrence:** recordNumber: AB0017; recordedBy: Abotsi, K.E.; **Taxon:** scientificName: Microgramma mauritiana (Willd.) Tardieu; namePublishedIn: Humbert, Fl. Madag. Fam. 5, 2: 108, t. 24(6-8) (1960); kingdom: Plantae; phylum: Pteridophyta; class: Polypodiopsida; order: Polypodiales; family: Polypodiaceae; genus: Microgramma; specificEpithet: mauritiana; scientificNameAuthorship: (Willd.) Tardieu; **Location:** continent: Africa; country: Togo; countryCode: TG; locality: Agou Nyogbo; verbatimElevation: 556; verbatimSRS: WGS84; decimalLatitude: 6.876847; decimalLongitude: 0.733947; geodeticDatum: WGS84; **Identification:** identifiedBy: Abotsi, K.E.; dateIdentified: 06-13-16; **Event:** eventDate: 06-13-16; habitat: Coffee/cocoa based agroforest; **Record Level:** institutionID: Herbarium Togoense; collectionID: Abotsi, K.E.; institutionCode: TOGO; basisOfRecord: Preserved specimen
- **Type status:** Other material. **Occurrence:** recordNumber: ASM 0324; recordedBy: Abotsi, K.E.; **Taxon:** scientificName: Microgramma mauritiana (Willd.) Tardieu; namePublishedIn: Humbert, Fl. Madag. Fam. 5, 2: 108, t. 24(6-8) (1960); kingdom: Plantae; phylum: Pteridophyta; class: Polypodiopsida; order: Polypodiales; family: Polypodiaceae; genus: Microgramma; specificEpithet: mauritiana; scientificNameAuthorship: (Willd.) Tardieu; **Location:** continent: Africa; country: Togo; countryCode: TG; locality: Mt Agou; verbatimElevation: 942; verbatimSRS: WGS84; decimalLatitude: 6.870710419; decimalLongitude: 0.751276064; geodeticDatum: WGS84; **Identification:** identifiedBy: Abotsi, K.E.; dateIdentified: 04-16-13; **Event:** eventDate: 04-16-13; habitat: Rainforest; **Record Level:** institutionID: Herbarium Togoense; collectionID: Abotsi, K.E.; institutionCode: TOGO; basisOfRecord: Preserved specimen

##### Ecological interactions

###### Native status

Native

##### Distribution

Zone 4

#### Tectariaceae

#### Arthropteris orientalis

(Gmel.) Posth.

##### Materials

- **Type status:** Other material. **Occurrence:** catalogNumber: 12470; recordNumber: 8965; recordedBy: Brunel, J.-F.; **Taxon:** scientificName: Arthropteris orientalis (Gmel.) Posth.; namePublishedIn: Rec. Trav. Bot. néerl. 21: 218 (1924); kingdom: Plantae; phylum: Pteridophyta; class: Polypodiopsida; order: Polypodiales; family: Tectariaceae; genus: Arthropteris; specificEpithet: orientalis; scientificNameAuthorship: (Gmel.) Posth.; **Location:** continent: Africa; country: Togo; countryCode: TG; locality: Kouma Konda; verbatimElevation: 612; verbatimSRS: WGS84; decimalLatitude: 6.9549; decimalLongitude: 0.5795; geodeticDatum: WGS84; **Identification:** identifiedBy: Brunel, J.-F.; dateIdentified: 10-01-84; **Event:** eventDate: 10-01-84; habitat: Rainforest; **Record Level:** institutionID: Herbarium Togoense; collectionID: Brunel, J.-F.; institutionCode: TOGO; basisOfRecord: Preserved specimen
- **Type status:** Other material. **Occurrence:** catalogNumber: 12471; recordNumber: 1489; recordedBy: Akpagana, K.; **Taxon:** scientificName: Arthropteris orientalis (Gmel.) Posth.; namePublishedIn: Rec. Trav. Bot. néerl. 21: 218 (1924); kingdom: Plantae; phylum: Pteridophyta; class: Polypodiopsida; order: Polypodiales; family: Tectariaceae; genus: Arthropteris; specificEpithet: orientalis; scientificNameAuthorship: (Gmel.) Posth.; **Location:** continent: Africa; country: Togo; countryCode: TG; locality: Agbo kopé; verbatimElevation: 658; verbatimSRS: WGS84; decimalLatitude: 7.43116; decimalLongitude: 0.7085; geodeticDatum: WGS84; **Identification:** identifiedBy: K. Akpagana; dateIdentified: 03-01-87; **Event:** eventDate: 03-01-87; habitat: Rainforest; **Record Level:** institutionID: Herbarium Togoense; collectionID: Akpagana, K.; institutionCode: TOGO; basisOfRecord: Preserved specimen
- **Type status:** Other material. **Occurrence:** catalogNumber: 12487; recordNumber: 5560; recordedBy: Brunel, J.-F.; **Taxon:** scientificName: Arthropteris orientalis (Gmel.) Posth.; namePublishedIn: Rec. Trav. Bot. néerl. 21: 218 (1924); kingdom: Plantae; phylum: Pteridophyta; class: Polypodiopsida; order: Polypodiales; family: Tectariaceae; genus: Arthropteris; specificEpithet: orientalis; scientificNameAuthorship: (Gmel.) Posth.; **Location:** continent: Africa; country: Togo; countryCode: TG; locality: Kpimé; verbatimElevation: 302; verbatimSRS: WGS84; decimalLatitude: 7.003; decimalLongitude: 0.644; geodeticDatum: WGS84; **Identification:** identifiedBy: Brunel, J.-F.; dateIdentified: 1980; **Event:** eventDate: 12-01-78; habitat: Rainforest; **Record Level:** institutionID: Herbarium Togoense; collectionID: Brunel, J.-F.; institutionCode: TOGO; basisOfRecord: Preserved specimen

##### Ecological interactions

###### Native status

Native

##### Distribution

Zone 4

#### Tectaria angelicifolia

(Schum.) Copel.

##### Materials

- **Type status:** Other material. **Occurrence:** recordNumber: AB0241; recordedBy: Abotsi, K.E.; **Taxon:** scientificName: Tectaria angelicifolia (Schum.) Copel.; namePublishedIn: Phil. Journ. Sci. 2C: 410 (1907); kingdom: Plantae; phylum: Pteridophyta; class: Polypodiopsida; order: Polypodiales; family: Tectariaceae; genus: Tectaria; specificEpithet: angelicifolia; scientificNameAuthorship: (Schum.) Copel.; **Location:** continent: Africa; country: Togo; countryCode: TG; locality: Akloa; verbatimElevation: 369; verbatimSRS: WGS84; decimalLatitude: 7.514911; decimalLongitude: 0.619492; geodeticDatum: WGS84; **Identification:** identifiedBy: Abotsi, K.E.; dateIdentified: 08-06-16; **Event:** eventDate: 08-06-16; habitat: Rainforest; **Record Level:** institutionID: Herbarium Togoense; collectionID: Abotsi, K.E.; institutionCode: TOGO; basisOfRecord: Preserved specimen
- **Type status:** Other material. **Occurrence:** recordNumber: AB0506; recordedBy: Abotsi, K.E.; **Taxon:** scientificName: Tectaria angelicifolia (Schum.) Copel.; namePublishedIn: Phil. Journ. Sci. 2C: 410 (1907); kingdom: Plantae; phylum: Pteridophyta; class: Polypodiopsida; order: Polypodiales; family: Tectariaceae; genus: Tectaria; specificEpithet: angelicifolia; scientificNameAuthorship: (Schum.) Copel.; **Location:** continent: Africa; country: Togo; countryCode: TG; locality: Gbadi Gawodo; verbatimElevation: 638; verbatimSRS: WGS84; decimalLatitude: 7.484815; decimalLongitude: 0.76634; geodeticDatum: WGS84; **Identification:** identifiedBy: Abotsi, K.E.; dateIdentified: 04-26-17; **Event:** eventDate: 04-26-17; habitat: Rainforest; **Record Level:** institutionID: Herbarium Togoense; collectionID: Abotsi, K.E.; institutionCode: TOGO; basisOfRecord: Preserved specimen
- **Type status:** Other material. **Occurrence:** recordNumber: AB0032; recordedBy: Abotsi, K.E.; **Taxon:** scientificName: Tectaria angelicifolia (Schum.) Copel.; namePublishedIn: Phil. Journ. Sci. 2C: 410 (1907); kingdom: Plantae; phylum: Pteridophyta; class: Polypodiopsida; order: Polypodiales; family: Tectariaceae; genus: Tectaria; specificEpithet: angelicifolia; scientificNameAuthorship: (Schum.) Copel.; **Location:** continent: Africa; country: Togo; countryCode: TG; locality: Agou Kébo-Dzigbé; verbatimElevation: 765; verbatimSRS: WGS84; decimalLatitude: 6.863635; decimalLongitude: 0.75568; geodeticDatum: WGS84; **Identification:** identifiedBy: Abotsi, K.E.; dateIdentified: 06-14-16; **Event:** eventDate: 06-14-16; habitat: Rainforest; **Record Level:** institutionID: Herbarium Togoense; collectionID: Abotsi, K.E.; institutionCode: TOGO; basisOfRecord: Preserved specimen

##### Ecological interactions

###### Native status

Native

##### Distribution

Zone 4

#### Tectaria fernandensis

(Baker) C.Chr.

##### Materials

- **Type status:** Other material. **Occurrence:** recordNumber: AB0045; recordedBy: Abotsi, K.E.; **Taxon:** scientificName: Tectaria fernandensis (Baker) C.Chr.; namePublishedIn: Ind. Fil., Suppl. III: 179 (1934); kingdom: Plantae; phylum: Pteridophyta; class: Polypodiopsida; order: Polypodiales; family: Tectariaceae; genus: Tectaria; specificEpithet: fernandensis; scientificNameAuthorship: (Baker) C.Chr.; **Location:** continent: Africa; country: Togo; countryCode: TG; locality: Agomé Yoh; verbatimElevation: 371; verbatimSRS: WGS84; decimalLatitude: 6.950836; decimalLongitude: 0.597365; geodeticDatum: WGS84; **Identification:** identifiedBy: Abotsi, K.E.; dateIdentified: 06-15-16; **Event:** eventDate: 06-15-16; habitat: Coffee/cocoa based agroforest; **Record Level:** institutionID: Herbarium Togoense; collectionID: Abotsi, K.E.; institutionCode: TOGO; basisOfRecord: Preserved specimen
- **Type status:** Other material. **Occurrence:** recordNumber: AB0255; recordedBy: Abotsi, K.E.; **Taxon:** scientificName: Tectaria fernandensis (Baker) C.Chr.; namePublishedIn: Ind. Fil., Suppl. III: 179 (1934); kingdom: Plantae; phylum: Pteridophyta; class: Polypodiopsida; order: Polypodiales; family: Tectariaceae; genus: Tectaria; specificEpithet: fernandensis; scientificNameAuthorship: (Baker) C.Chr.; **Location:** continent: Africa; country: Togo; countryCode: TG; locality: Badou; verbatimElevation: 323; verbatimSRS: WGS84; decimalLatitude: 7.581904; decimalLongitude: 0.621121; geodeticDatum: WGS84; **Identification:** identifiedBy: Abotsi, K.E.; dateIdentified: 08-08-16; **Event:** eventDate: 08-08-16; habitat: Rainforest; **Record Level:** institutionID: Herbarium Togoense; collectionID: Abotsi, K.E.; institutionCode: TOGO; basisOfRecord: Preserved specimen
- **Type status:** Other material. **Occurrence:** recordNumber: AB0405; recordedBy: Abotsi, K.E.; **Taxon:** scientificName: Tectaria fernandensis (Baker) C.Chr.; namePublishedIn: Ind. Fil., Suppl. III: 179 (1934); kingdom: Plantae; phylum: Pteridophyta; class: Polypodiopsida; order: Polypodiales; family: Tectariaceae; genus: Tectaria; specificEpithet: fernandensis; scientificNameAuthorship: (Baker) C.Chr.; **Location:** continent: Africa; country: Togo; countryCode: TG; locality: Agou Kébo-Dzigbé; verbatimElevation: 847; verbatimSRS: WGS84; decimalLatitude: 6.867475; decimalLongitude: 0.751468; geodeticDatum: WGS84; **Identification:** identifiedBy: Abotsi, K.E.; dateIdentified: 12-26-16; **Event:** eventDate: 12-26-16; habitat: Rainforest; **Record Level:** institutionID: Herbarium Togoense; collectionID: Abotsi, K.E.; institutionCode: TOGO; basisOfRecord: Preserved specimen

##### Ecological interactions

###### Native status

Native

##### Distribution

Zone 4

#### Triplophyllum buchholzii

(Kuhn) Holttum

##### Materials

- **Type status:** Other material. **Occurrence:** recordNumber: ASM 0245; recordedBy: Abotsi, K.E., Sodjinou E. & Mingou P.; **Taxon:** scientificName: Triplophyllum buchholzii (Kuhn) Holttum; namePublishedIn: Kew Bull. 41(2): 251 (1986); kingdom: Plantae; phylum: Pteridophyta; class: Polypodiopsida; order: Polypodiales; family: Tectariaceae; genus: Triplophyllum; specificEpithet: buchholzii; scientificNameAuthorship: (Kuhn) Holttum; **Location:** continent: Africa; country: Togo; countryCode: TG; locality: Danyi N'Digbé; verbatimElevation: 558; verbatimSRS: WGS84; decimalLatitude: 7.121720933; decimalLongitude: 0.653457481; geodeticDatum: WGS84; **Identification:** identifiedBy: Abotsi, K.E.; dateIdentified: 04-09-13; **Event:** eventDate: 04-09-13; habitat: Rainforest; **Record Level:** institutionID: Herbarium Togoense; collectionID: Abotsi, K.E.; institutionCode: TOGO; basisOfRecord: Preserved specimen
- **Type status:** Other material. **Occurrence:** catalogNumber: 12142; recordNumber: 5495; recordedBy: Brunel, J.-F.; **Taxon:** scientificName: Triplophyllum buchholzii (Kuhn) Holttum; namePublishedIn: Kew Bull. 41(2): 251 (1986); kingdom: Plantae; phylum: Pteridophyta; class: Polypodiopsida; order: Polypodiales; family: Tectariaceae; genus: Triplophyllum; specificEpithet: buchholzii; scientificNameAuthorship: (Kuhn) Holttum; **Location:** continent: Africa; country: Togo; countryCode: TG; locality: Agou Nyogbo; verbatimElevation: 470; verbatimSRS: WGS84; decimalLatitude: 6.895432; decimalLongitude: 0.738209; geodeticDatum: WGS84; **Identification:** identifiedBy: Brunel, J.-F.; dateIdentified: /10/1978; **Event:** eventDate: /10/1978; habitat: Rainforest; **Record Level:** institutionID: Herbarium Togoense; collectionID: Brunel, J.-F.; institutionCode: TOGO; basisOfRecord: Preserved specimen

##### Ecological interactions

###### Native status

Native

##### Distribution

Zone 4

#### Triplophyllum heudelotii

Pic. Serm.

##### Materials

- **Type status:** Other material. **Occurrence:** recordedBy: Roux, J.P.; **Taxon:** scientificName: Triplophyllum heudelotii Pic. Serm.; namePublishedIn: Webbia 45(1): 126 (1991); kingdom: Plantae; phylum: Pteridophyta; class: Polypodiopsida; order: Polypodiales; family: Tectariaceae; genus: Triplophyllum; specificEpithet: heudelotii; scientificNameAuthorship: Pic. Serm.; **Location:** continent: Africa; country: Togo; countryCode: TG; **Event:** habitat: Rainforest; **Record Level:** basisOfRecord: Unknown; source: Synopsis of the Lycopodiophyta and Pteridophyta of Africa, Madagascar and neighbouring islands (Roux, 2009)

##### Ecological interactions

###### Native status

Native

##### Distribution

Uncertain

#### Triplophyllum jenseniae

(C. Chr.) Holttum

##### Materials

- **Type status:** Other material. **Occurrence:** catalogNumber: 12141; recordNumber: 2382; recordedBy: Akpagana, K.; **Taxon:** scientificName: Triplophyllum jenseniae (C. Chr.) Holttum; namePublishedIn: Kew Bull. 41(2): 253 (1986); kingdom: Plantae; phylum: Pteridophyta; class: Polypodiopsida; order: Polypodiales; family: Tectariaceae; genus: Triplophyllum; specificEpithet: jenseniae; scientificNameAuthorship: (C. Chr.) Holttum; **Location:** continent: Africa; country: Togo; countryCode: TG; locality: Danyi; verbatimElevation: 706; verbatimSRS: WGS84; decimalLatitude: 7.169022; decimalLongitude: 0.68099; geodeticDatum: WGS84; **Identification:** identifiedBy: K. Akpagana; dateIdentified: /02/1991; **Event:** eventDate: /02/1991; habitat: Rainforest; **Record Level:** institutionID: Herbarium Togoense; collectionID: Akpagana, K.; institutionCode: TOGO; basisOfRecord: Preserved specimen
- **Type status:** Other material. **Occurrence:** catalogNumber: 12140; recordNumber: 10074; recordedBy: Brunel, J.-F.; **Taxon:** scientificName: Triplophyllum jenseniae (C. Chr.) Holttum; namePublishedIn: Kew Bull. 41(2): 253 (1986); kingdom: Plantae; phylum: Pteridophyta; class: Polypodiopsida; order: Polypodiales; family: Tectariaceae; genus: Triplophyllum; specificEpithet: jenseniae; scientificNameAuthorship: (C. Chr.) Holttum; **Location:** continent: Africa; country: Togo; countryCode: TG; locality: Agadja; verbatimElevation: 656; verbatimSRS: WGS84; decimalLatitude: 7.491346; decimalLongitude: 0.770762; geodeticDatum: WGS84; **Identification:** identifiedBy: Brunel, J.-F.; dateIdentified: /03/1987; **Event:** eventDate: /03/1987; habitat: Rainforest; **Record Level:** institutionID: Herbarium Togoense; collectionID: Brunel, J.-F.; institutionCode: TOGO; basisOfRecord: Preserved specimen

##### Ecological interactions

###### Native status

Native

##### Distribution

Zone 4

#### Triplophyllum pilosissimum

(J. Sm. ex T. Moore) Holttum

##### Materials

- **Type status:** Other material. **Occurrence:** catalogNumber: 12150; recordNumber: 8133; recordedBy: Brunel, J.-F.; **Taxon:** scientificName: Triplophyllum pilosissimum (J. Sm. ex T. Moore) Holttum; namePublishedIn: Kew Bull. 41(2): 246 (1986); kingdom: Plantae; phylum: Pteridophyta; class: Polypodiopsida; order: Polypodiales; family: Tectariaceae; genus: Triplophyllum; specificEpithet: pilosissimum; scientificNameAuthorship: (J. Sm. ex T. Moore) Holttum; **Location:** continent: Africa; country: Togo; countryCode: TG; locality: Kougnohou; verbatimElevation: 669; verbatimSRS: WGS84; decimalLatitude: 7.636132; decimalLongitude: 0.796726; geodeticDatum: WGS84; **Identification:** identifiedBy: Brunel, J.-F.; dateIdentified: /12/1983; **Event:** eventDate: /12/1983; habitat: Rainforest; **Record Level:** institutionID: Herbarium Togoense; collectionID: Brunel, J.-F.; institutionCode: TOGO; basisOfRecord: Preserved specimen

##### Ecological interactions

###### Native status

Native

##### Distribution

Zone 4

#### Triplophyllum protensum

(Afzel. ex Sw.) Holttum

##### Materials

- **Type status:** Other material. **Occurrence:** recordNumber: AB0504; recordedBy: Abotsi, K.E.; **Taxon:** scientificName: Triplophyllum protensum (Afzel. ex Sw.) Holttum; namePublishedIn: Kew Bull. 41(2): 247 (1986); kingdom: Plantae; phylum: Pteridophyta; class: Polypodiopsida; order: Polypodiales; family: Tectariaceae; genus: Triplophyllum; specificEpithet: protensum; scientificNameAuthorship: (Afzel. ex Sw.) Holttum; **Location:** continent: Africa; country: Togo; countryCode: TG; locality: Gbadi Gawodo; verbatimElevation: 635; verbatimSRS: WGS84; decimalLatitude: 7.484742; decimalLongitude: 0.766472; geodeticDatum: WGS84; **Identification:** identifiedBy: Abotsi, K.E.; dateIdentified: 04-26-17; **Event:** eventDate: 04-26-17; habitat: Rainforest; **Record Level:** institutionID: Herbarium Togoense; collectionID: Abotsi, K.E.; institutionCode: TOGO; basisOfRecord: Preserved specimen
- **Type status:** Other material. **Occurrence:** recordNumber: AB0508; recordedBy: Abotsi, K.E.; **Taxon:** scientificName: Triplophyllum protensum (Afzel. ex Sw.) Holttum; namePublishedIn: Kew Bull. 41(2): 247 (1986); kingdom: Plantae; phylum: Pteridophyta; class: Polypodiopsida; order: Polypodiales; family: Tectariaceae; genus: Triplophyllum; specificEpithet: protensum; scientificNameAuthorship: (Afzel. ex Sw.) Holttum; **Location:** continent: Africa; country: Togo; countryCode: TG; locality: Gbadi Gawodo; verbatimElevation: 639; verbatimSRS: WGS84; decimalLatitude: 7.484761; decimalLongitude: 0.766336; geodeticDatum: WGS84; **Identification:** identifiedBy: Abotsi, K.E.; dateIdentified: 04-26-17; **Event:** eventDate: 04-26-17; habitat: Rainforest; **Record Level:** institutionID: Herbarium Togoense; collectionID: Abotsi, K.E.; institutionCode: TOGO; basisOfRecord: Preserved specimen
- **Type status:** Other material. **Occurrence:** catalogNumber: 12106; recordNumber: 71; recordedBy: Brunel, J.-F.; **Taxon:** scientificName: Triplophyllum protensum (Afzel. ex Sw.) Holttum; namePublishedIn: Kew Bull. 41(2): 247 (1986); kingdom: Plantae; phylum: Pteridophyta; class: Polypodiopsida; order: Polypodiales; family: Tectariaceae; genus: Triplophyllum; specificEpithet: protensum; scientificNameAuthorship: (Afzel. ex Sw.) Holttum; **Location:** continent: Africa; country: Togo; countryCode: TG; locality: Yoh; verbatimSRS: WGS84; decimalLatitude: 7; decimalLongitude: 0.75; geodeticDatum: WGS84; **Identification:** identifiedBy: Brunel, J.-F.; dateIdentified: 1980; **Event:** eventDate: 05-26-05; habitat: Rainforest; **Record Level:** institutionID: Herbarium Togoense; collectionID: Brunel, J.-F.; institutionCode: TOGO; basisOfRecord: Preserved specimen

##### Ecological interactions

###### Native status

Native

##### Distribution

Zone 4

#### Triplophyllum speciosum

(Mett. ex Kuhn) Holttum

##### Materials

- **Type status:** Other material. **Occurrence:** catalogNumber: 12153; recordNumber: 10085; recordedBy: Brunel, J.-F.; **Taxon:** scientificName: Triplophyllum speciosum (Mett. ex Kuhn) Holttum; namePublishedIn: Kew Bull. 41(2): 247 (1986); kingdom: Plantae; phylum: Pteridophyta; class: Polypodiopsida; order: Polypodiales; family: Tectariaceae; genus: Triplophyllum; specificEpithet: speciosum; scientificNameAuthorship: (Mett. ex Kuhn) Holttum; **Location:** continent: Africa; country: Togo; countryCode: TG; locality: Agadja; verbatimElevation: 656; verbatimSRS: WGS84; decimalLatitude: 7.491346; decimalLongitude: 0.770762; geodeticDatum: WGS84; **Identification:** identifiedBy: Brunel, J.-F.; dateIdentified: /03/1987; **Event:** eventDate: /03/1987; habitat: Rainforest; **Record Level:** institutionID: Herbarium Togoense; collectionID: Brunel, J.-F.; institutionCode: TOGO; basisOfRecord: Preserved specimen
- **Type status:** Other material. **Occurrence:** catalogNumber: 12152; recordNumber: 1476; recordedBy: Akpagana, K.; **Taxon:** scientificName: Triplophyllum speciosum (Mett. ex Kuhn) Holttum; namePublishedIn: Kew Bull. 41(2): 247 (1986); kingdom: Plantae; phylum: Pteridophyta; class: Polypodiopsida; order: Polypodiales; family: Tectariaceae; genus: Triplophyllum; specificEpithet: speciosum; scientificNameAuthorship: (Mett. ex Kuhn) Holttum; **Location:** continent: Africa; country: Togo; countryCode: TG; locality: Gbadi N'Kougna; verbatimElevation: 691; verbatimSRS: WGS84; decimalLatitude: 7.44316; decimalLongitude: 0.706923; geodeticDatum: WGS84; **Identification:** identifiedBy: Akpagana, K.; dateIdentified: /03/1987; **Event:** eventDate: /03/1987; habitat: Rainforest; **Record Level:** institutionID: Herbarium Togoense; collectionID: Akpagana, K.; institutionCode: TOGO; basisOfRecord: Preserved specimen

##### Ecological interactions

###### Native status

Native

##### Distribution

Zone 4

#### Triplophyllum subquinquefidum

(P. Beauv.) Pic. Serm.

##### Materials

- **Type status:** Other material. **Occurrence:** recordedBy: Roux, J.P.; **Taxon:** scientificName: Triplophyllum subquinquefidum (P. Beauv.) Pic. Serm.; namePublishedIn: Webbia 45(1): 129 (1991); kingdom: Plantae; phylum: Pteridophyta; class: Polypodiopsida; order: Polypodiales; family: Tectariaceae; genus: Triplophyllum; specificEpithet: subquinquefidum; scientificNameAuthorship: (P. Beauv.) Pic. Serm.; **Location:** continent: Africa; country: Togo; countryCode: TG; **Event:** habitat: Rainforest; **Record Level:** basisOfRecord: Unknown; source: Synopsis of the Lycopodiophyta and Pteridophyta of Africa, Madagascar and neighbouring islands (Roux, 2009)

##### Ecological interactions

###### Native status

Native

##### Distribution

Zone 4

#### Triplophyllum vogelii

(Hook.) Holttum

##### Materials

- **Type status:** Other material. **Occurrence:** recordNumber: AB0097; recordedBy: Abotsi, K.E.; **Taxon:** scientificName: Triplophyllum vogelii (Hook.) Holttum; namePublishedIn: Kew Bull. 41(2): 249 (1986); kingdom: Plantae; phylum: Pteridophyta; class: Polypodiopsida; order: Polypodiales; family: Tectariaceae; genus: Triplophyllum; specificEpithet: vogelii; scientificNameAuthorship: (Hook.) Holttum; **Location:** continent: Africa; country: Togo; countryCode: TG; locality: Danyi N'Digbé; verbatimElevation: 692; verbatimSRS: WGS84; decimalLatitude: 7.151174; decimalLongitude: 0.673605; geodeticDatum: WGS84; **Identification:** identifiedBy: Abotsi, K.E.; dateIdentified: 06-27-16; **Event:** eventDate: 06-27-16; habitat: Rainforest; **Record Level:** institutionID: Herbarium Togoense; collectionID: Abotsi, K.E.; institutionCode: TOGO; basisOfRecord: Preserved specimen
- **Type status:** Other material. **Occurrence:** recordNumber: AB0325; recordedBy: Abotsi, K.E.; **Taxon:** scientificName: Triplophyllum vogelii (Hook.) Holttum; namePublishedIn: Kew Bull. 41(2): 249 (1986); kingdom: Plantae; phylum: Pteridophyta; class: Polypodiopsida; order: Polypodiales; family: Tectariaceae; genus: Triplophyllum; specificEpithet: vogelii; scientificNameAuthorship: (Hook.) Holttum; **Location:** continent: Africa; country: Togo; countryCode: TG; locality: Assoukoko; verbatimElevation: 337; verbatimSRS: WGS84; decimalLatitude: 8.003312; decimalLongitude: 0.613371; geodeticDatum: WGS84; **Identification:** identifiedBy: Abotsi, K.E.; dateIdentified: 09-18-16; **Event:** eventDate: 09-18-16; habitat: Rainforest; **Record Level:** institutionID: Herbarium Togoense; collectionID: Abotsi, K.E.; institutionCode: TOGO; basisOfRecord: Preserved specimen
- **Type status:** Other material. **Occurrence:** recordNumber: AB0063; recordedBy: Abotsi, K.E.; **Taxon:** scientificName: Triplophyllum vogelii (Hook.) Holttum; namePublishedIn: Kew Bull. 41(2): 249 (1986); kingdom: Plantae; phylum: Pteridophyta; class: Polypodiopsida; order: Polypodiales; family: Tectariaceae; genus: Triplophyllum; specificEpithet: vogelii; scientificNameAuthorship: (Hook.) Holttum; **Location:** continent: Africa; country: Togo; countryCode: TG; locality: Missahoé; verbatimElevation: 529; verbatimSRS: WGS84; decimalLatitude: 6.946928; decimalLongitude: 0.580442; geodeticDatum: WGS84; **Identification:** identifiedBy: Abotsi, K.E.; dateIdentified: 06-24-16; **Event:** eventDate: 06-24-16; habitat: Rainforest; **Record Level:** institutionID: Herbarium Togoense; collectionID: Abotsi, K.E.; institutionCode: TOGO; basisOfRecord: Preserved specimen

##### Ecological interactions

###### Native status

Native

##### Distribution

Zone 4

#### Pteridineae

#### Pteridaceae

#### Cheilanthoideae

#### Doryopteris kirkii

(Hook.) Alston

##### Materials

- **Type status:** Other material. **Occurrence:** recordNumber: AB0210; recordedBy: Abotsi, K.E.; **Taxon:** scientificName: Doryopteris kirkii (Hook.) Alston; namePublishedIn: Bol. Soc. Broter. ser. 2, 80: 14 (1956); kingdom: Plantae; phylum: Pteridophyta; class: Polypodiopsida; order: Polypodiales; family: Pteridaceae; genus: Doryopteris; specificEpithet: kirkii; scientificNameAuthorship: (Hook.) Alston; **Location:** continent: Africa; country: Togo; countryCode: TG; locality: Kpimé Séva; verbatimElevation: 525; verbatimSRS: WGS84; decimalLatitude: 7.0169; decimalLongitude: 0.640521; geodeticDatum: WGS84; **Identification:** identifiedBy: Abotsi, K.E.; dateIdentified: 08-04-16; **Event:** eventDate: 08-04-16; habitat: Rainforest; **Record Level:** institutionID: Herbarium Togoense; collectionID: Abotsi, K.E.; institutionCode: TOGO; basisOfRecord: Preserved specimen
- **Type status:** Other material. **Occurrence:** recordNumber: AB0226; recordedBy: Abotsi, K.E.; **Taxon:** scientificName: Doryopteris kirkii (Hook.) Alston; namePublishedIn: Bol. Soc. Broter. ser. 2, 80: 14 (1956); kingdom: Plantae; phylum: Pteridophyta; class: Polypodiopsida; order: Polypodiales; family: Pteridaceae; genus: Doryopteris; specificEpithet: kirkii; scientificNameAuthorship: (Hook.) Alston; **Location:** continent: Africa; country: Togo; countryCode: TG; locality: Bèna Olé; verbatimElevation: 707; verbatimSRS: WGS84; decimalLatitude: 7.555646; decimalLongitude: 0.863315; geodeticDatum: WGS84; **Identification:** identifiedBy: Abotsi, K.E.; dateIdentified: 08-05-16; **Event:** eventDate: 08-05-16; habitat: Coffee/cocoa based agroforest; **Record Level:** institutionID: Herbarium Togoense; collectionID: Abotsi, K.E.; institutionCode: TOGO; basisOfRecord: Preserved specimen
- **Type status:** Other material. **Occurrence:** recordNumber: AB0312; recordedBy: Abotsi, K.E.; **Taxon:** scientificName: Doryopteris kirkii (Hook.) Alston; namePublishedIn: Bol. Soc. Broter. ser. 2, 80: 14 (1956); kingdom: Plantae; phylum: Pteridophyta; class: Polypodiopsida; order: Polypodiales; family: Pteridaceae; genus: Doryopteris; specificEpithet: kirkii; scientificNameAuthorship: (Hook.) Alston; **Location:** continent: Africa; country: Togo; countryCode: TG; locality: Assoukoko; verbatimElevation: 635; verbatimSRS: WGS84; decimalLatitude: 8.004828; decimalLongitude: 0.625982; geodeticDatum: WGS84; **Identification:** identifiedBy: Abotsi, K.E.; dateIdentified: 09-18-16; **Event:** eventDate: 09-18-16; habitat: Rainforest; **Record Level:** institutionID: Herbarium Togoense; collectionID: Abotsi, K.E.; institutionCode: TOGO; basisOfRecord: Preserved specimen

##### Ecological interactions

###### Native status

Native

##### Distribution

Zone 4

#### Pellaea doniana

(J. Sm.) Hook.

##### Materials

- **Type status:** Other material. **Occurrence:** recordNumber: AB0171; recordedBy: Abotsi, K.E.; **Taxon:** scientificName: Pellaea doniana (J. Sm.) Hook.; namePublishedIn: Sp. 2: 137, t. 125 A (1858); kingdom: Plantae; phylum: Pteridophyta; class: Polypodiopsida; order: Polypodiales; family: Pteridaceae; genus: Pellaea; specificEpithet: doniana; scientificNameAuthorship: (J. Sm.) Hook.; **Location:** continent: Africa; country: Togo; countryCode: TG; locality: Kpimé Séva; verbatimElevation: 323; verbatimSRS: WGS84; decimalLatitude: 7.007999; decimalLongitude: 0.645665; geodeticDatum: WGS84; **Identification:** identifiedBy: Abotsi, K.E.; dateIdentified: 07-23-16; **Event:** eventDate: 07-23-16; habitat: Rainforest; **Record Level:** institutionID: Herbarium Togoense; collectionID: Abotsi, K.E.; institutionCode: TOGO; basisOfRecord: Preserved specimen
- **Type status:** Other material. **Occurrence:** recordNumber: AB0231; recordedBy: Abotsi, K.E.; **Taxon:** scientificName: Pellaea doniana (J. Sm.) Hook.; namePublishedIn: Sp. 2: 137, t. 125 A (1858); kingdom: Plantae; phylum: Pteridophyta; class: Polypodiopsida; order: Polypodiales; family: Pteridaceae; genus: Pellaea; specificEpithet: doniana; scientificNameAuthorship: (J. Sm.) Hook.; **Location:** continent: Africa; country: Togo; countryCode: TG; locality: Akloa; verbatimElevation: 321; verbatimSRS: WGS84; decimalLatitude: 7.514973; decimalLongitude: 0.615899; geodeticDatum: WGS84; **Identification:** identifiedBy: Abotsi, K.E.; dateIdentified: 08-06-16; **Event:** eventDate: 08-06-16; habitat: Rainforest; **Record Level:** institutionID: Herbarium Togoense; collectionID: Abotsi, K.E.; institutionCode: TOGO; basisOfRecord: Preserved specimen
- **Type status:** Other material. **Occurrence:** recordNumber: AB0303; recordedBy: Abotsi, K.E.; **Taxon:** scientificName: Pellaea doniana (J. Sm.) Hook.; namePublishedIn: Sp. 2: 137, t. 125 A (1858); kingdom: Plantae; phylum: Pteridophyta; class: Polypodiopsida; order: Polypodiales; family: Pteridaceae; genus: Pellaea; specificEpithet: doniana; scientificNameAuthorship: (J. Sm.) Hook.; **Location:** continent: Africa; country: Togo; countryCode: TG; locality: Aménosodzi; verbatimElevation: 265; verbatimSRS: WGS84; decimalLatitude: 7.903048; decimalLongitude: 0.650699; geodeticDatum: WGS84; **Identification:** identifiedBy: Abotsi, K.E.; dateIdentified: 09-17-16; **Event:** eventDate: 09-17-16; habitat: Rainforest; **Record Level:** institutionID: Herbarium Togoense; collectionID: Abotsi, K.E.; institutionCode: TOGO; basisOfRecord: Preserved specimen

##### Ecological interactions

###### Native status

Native

##### Distribution

Zone 4

#### Parkerioideae

#### Acrostichum aureum

L.

##### Materials

- **Type status:** Other material. **Occurrence:** recordNumber: AB0468; recordedBy: Abotsi, K.E.; **Taxon:** scientificName: Acrostichum aureum L.; namePublishedIn: Sp. Pl. 2: 1069 (1753)]; kingdom: Plantae; phylum: Pteridophyta; class: Polypodiopsida; order: Polypodiales; family: Pteridaceae; genus: Acrostichum; specificEpithet: aureum; scientificNameAuthorship: L.; **Location:** continent: Africa; country: Togo; countryCode: TG; locality: Zanvé; verbatimElevation: 4; verbatimSRS: WGS84; decimalLatitude: 6.282995; decimalLongitude: 1.749436; geodeticDatum: WGS84; **Identification:** identifiedBy: Abotsi, K.E.; dateIdentified: 02-01-17; **Event:** eventDate: 02-01-17; habitat: Flooded savannah; **Record Level:** institutionID: Herbarium Togoense; collectionID: Abotsi, K.E.; institutionCode: TOGO; basisOfRecord: Preserved specimen
- **Type status:** Other material. **Occurrence:** recordNumber: AB0609; recordedBy: Abotsi, K.E.; **Taxon:** scientificName: Acrostichum aureum L.; namePublishedIn: Sp. Pl. 2: 1069 (1753)]; kingdom: Plantae; phylum: Pteridophyta; class: Polypodiopsida; order: Polypodiales; family: Pteridaceae; genus: Acrostichum; specificEpithet: aureum; scientificNameAuthorship: L.; **Location:** continent: Africa; country: Togo; countryCode: TG; locality: Godjinmé; verbatimElevation: 60; verbatimSRS: WGS84; decimalLatitude: 6.71972; decimalLongitude: 1.51583; geodeticDatum: WGS84; **Identification:** identifiedBy: Abotsi, K.E.; dateIdentified: 08-30-17; **Event:** eventDate: 08-30-17; habitat: Rainforest; **Record Level:** institutionID: Herbarium Togoense; collectionID: Abotsi, K.E.; institutionCode: TOGO; basisOfRecord: Preserved specimen
- **Type status:** Other material. **Occurrence:** recordNumber: AB0145; recordedBy: Abotsi, K.E.; **Taxon:** scientificName: Acrostichum aureum L.; namePublishedIn: Sp. Pl. 2: 1069 (1753)]; kingdom: Plantae; phylum: Pteridophyta; class: Polypodiopsida; order: Polypodiales; family: Pteridaceae; genus: Acrostichum; specificEpithet: aureum; scientificNameAuthorship: L.; **Location:** continent: Africa; country: Togo; countryCode: TG; locality: Zowlagan; verbatimElevation: 5; verbatimSRS: WGS84; decimalLatitude: 6.273027; decimalLongitude: 1.570112; geodeticDatum: WGS84; **Identification:** identifiedBy: Abotsi, K.E.; dateIdentified: 07-11-16; **Event:** eventDate: 07-11-16; habitat: Mangrove; **Record Level:** institutionID: Herbarium Togoense; collectionID: Abotsi, K.E.; institutionCode: TOGO; basisOfRecord: Preserved specimen

##### Ecological interactions

###### Native status

Native

##### Distribution

Zone 5

#### Ceratopteris thalictroides

(L.) Brongn.

##### Materials

- **Type status:** Other material. **Occurrence:** recordNumber: AB0466; recordedBy: Abotsi, K.E.; **Taxon:** scientificName: Ceratopteris thalictroides (L) Brongn.; namePublishedIn: Bull. Sci. Soc. Philom. Paris, sér. 3, 8: 186 (1821); kingdom: Plantae; phylum: Pteridophyta; class: Polypodiopsida; order: Polypodiales; family: Pteridaceae; genus: Ceratopteris; specificEpithet: thalictroides; scientificNameAuthorship: (L.) Brongn.; **Location:** continent: Africa; country: Togo; countryCode: TG; locality: Godjinmé; verbatimElevation: 65; verbatimSRS: WGS84; decimalLatitude: 6.71827; decimalLongitude: 1.521428; geodeticDatum: WGS84; **Identification:** identifiedBy: Abotsi, K.E.; dateIdentified: 01-27-17; **Event:** eventDate: 01-27-17; habitat: Rainforest; **Record Level:** institutionID: Herbarium Togoense; collectionID: Abotsi, K.E.; institutionCode: TOGO; basisOfRecord: Preserved specimen
- **Type status:** Other material. **Occurrence:** recordNumber: AB0471; recordedBy: Abotsi, K.E.; **Taxon:** scientificName: Ceratopteris thalictroides (L) Brongn.; namePublishedIn: Bull. Sci. Soc. Philom. Paris, sér. 3, 8: 186 (1821); kingdom: Plantae; phylum: Pteridophyta; class: Polypodiopsida; order: Polypodiales; family: Pteridaceae; genus: Ceratopteris; specificEpithet: thalictroides; scientificNameAuthorship: (L.) Brongn.; **Location:** continent: Africa; country: Togo; countryCode: TG; locality: Afito; verbatimElevation: 28; verbatimSRS: WGS84; decimalLatitude: 6.75203; decimalLongitude: 1.592489; geodeticDatum: WGS84; **Identification:** identifiedBy: Abotsi, K.E.; dateIdentified: 02-03-17; **Event:** eventDate: 02-03-17; habitat: Flooded savannah; **Record Level:** institutionID: Herbarium Togoense; collectionID: Abotsi, K.E.; institutionCode: TOGO; basisOfRecord: Preserved specimen
- **Type status:** Other material. **Occurrence:** recordNumber: AB0465; recordedBy: Abotsi, K.E.; **Taxon:** scientificName: Ceratopteris thalictroides (L) Brongn.; namePublishedIn: Bull. Sci. Soc. Philom. Paris, sér. 3, 8: 186 (1821); kingdom: Plantae; phylum: Pteridophyta; class: Polypodiopsida; order: Polypodiales; family: Pteridaceae; genus: Ceratopteris; specificEpithet: thalictroides; scientificNameAuthorship: (L.) Brongn.; **Location:** continent: Africa; country: Togo; countryCode: TG; locality: Godjinmé; verbatimElevation: 70; verbatimSRS: WGS84; decimalLatitude: 6.718534; decimalLongitude: 1.520319; geodeticDatum: WGS84; **Identification:** identifiedBy: Abotsi, K.E.; dateIdentified: 01-27-17; **Event:** eventDate: 01-27-17; habitat: Rainforest; **Record Level:** institutionID: Herbarium Togoense; collectionID: Abotsi, K.E.; institutionCode: TOGO; basisOfRecord: Preserved specimen

##### Ecological interactions

###### Native status

Native

##### Distribution

Zones 4 and 5

#### Pteridoideae

#### Actiniopteris radiata

(Koenig ex Sw.) Link.

##### Materials

- **Type status:** Other material. **Occurrence:** recordNumber: AB0546; recordedBy: Abotsi, K.E.; **Taxon:** scientificName: Actiniopteris radiata (Koenig ex Sw.) Link.; namePublishedIn: Fil. Sp. 80 (1841); kingdom: Plantae; phylum: Pteridophyta; class: Polypodiopsida; order: Polypodiales; family: Pteridaceae; genus: Actiniopteris; specificEpithet: radiata; scientificNameAuthorship: (Koenig ex Sw.) Link.; **Location:** continent: Africa; country: Togo; countryCode: TG; locality: Yadè Tchallada; verbatimElevation: 365; verbatimSRS: WGS84; decimalLatitude: 9.607637; decimalLongitude: 1.150443; geodeticDatum: WGS84; **Identification:** identifiedBy: Abotsi, K.E.; dateIdentified: 07-12-17; **Event:** eventDate: 07-12-17; habitat: Inhabited area, on a wall; **Record Level:** institutionID: Herbarium Togoense; collectionID: Abotsi, K.E.; institutionCode: TOGO; basisOfRecord: Preserved specimen

##### Ecological interactions

###### Native status

Native

##### Distribution

Zone 2

#### Pityrogramma calomelanos

(L.) Link.

##### Materials

- **Type status:** Other material. **Occurrence:** recordNumber: AB0169; recordedBy: Abotsi, K.E.; **Taxon:** scientificName: Pityrogramma calomelanos (L.) Link.; namePublishedIn: Handb. Gew. 3: 20 (1833); kingdom: Plantae; phylum: Pteridophyta; class: Polypodiopsida; order: Polypodiales; family: Pteridaceae; genus: Pityrogramma; specificEpithet: calomelanos; scientificNameAuthorship: (L.) Link.; **Location:** continent: Africa; country: Togo; countryCode: TG; locality: Kpimé Séva; verbatimElevation: 310; verbatimSRS: WGS84; decimalLatitude: 7.008411; decimalLongitude: 0.64556; geodeticDatum: WGS84; **Identification:** identifiedBy: Abotsi, K.E.; dateIdentified: 07-23-16; **Event:** eventDate: 07-23-16; habitat: Rainforest; **Record Level:** institutionID: Herbarium Togoense; collectionID: Abotsi, K.E.; institutionCode: TOGO; basisOfRecord: Preserved specimen
- **Type status:** Other material. **Occurrence:** recordNumber: AB0224; recordedBy: Abotsi, K.E.; **Taxon:** scientificName: Pityrogramma calomelanos (L.) Link.; namePublishedIn: Handb. Gew. 3: 20 (1833); kingdom: Plantae; phylum: Pteridophyta; class: Polypodiopsida; order: Polypodiales; family: Pteridaceae; genus: Pityrogramma; specificEpithet: calomelanos; scientificNameAuthorship: (L.) Link.; **Location:** continent: Africa; country: Togo; countryCode: TG; locality: Ounabè; verbatimElevation: 621; verbatimSRS: WGS84; decimalLatitude: 7.555633; decimalLongitude: 1.015385; geodeticDatum: WGS84; **Identification:** identifiedBy: Abotsi, K.E.; dateIdentified: 08-05-16; **Event:** eventDate: 08-05-16; habitat: Wooded savannah; **Record Level:** institutionID: Herbarium Togoense; collectionID: Abotsi, K.E.; institutionCode: TOGO; basisOfRecord: Preserved specimen
- **Type status:** Other material. **Occurrence:** recordNumber: AB0232; recordedBy: Abotsi, K.E.; **Taxon:** scientificName: Pityrogramma calomelanos (L.) Link.; namePublishedIn: Handb. Gew. 3: 20 (1833); kingdom: Plantae; phylum: Pteridophyta; class: Polypodiopsida; order: Polypodiales; family: Pteridaceae; genus: Pityrogramma; specificEpithet: calomelanos; scientificNameAuthorship: (L.) Link.; **Location:** continent: Africa; country: Togo; countryCode: TG; locality: Akloa; verbatimElevation: 332; verbatimSRS: WGS84; decimalLatitude: 7.515065; decimalLongitude: 0.615951; geodeticDatum: WGS84; **Identification:** identifiedBy: Abotsi, K.E.; dateIdentified: 08-06-16; **Event:** eventDate: 08-06-16; habitat: Rainforest; **Record Level:** institutionID: Herbarium Togoense; collectionID: Abotsi, K.E.; institutionCode: TOGO; basisOfRecord: Preserved specimen

##### Ecological interactions

###### Native status

Native

##### Distribution

Zones 2, 3 and 4

#### Pteris atrovirens

Willd.

##### Materials

- **Type status:** Other material. **Occurrence:** recordNumber: AB0201; recordedBy: Abotsi, K.E.; **Taxon:** scientificName: Pteris atrovirens Willd.; namePublishedIn: Sp. 5: 385 (1810); kingdom: Plantae; phylum: Pteridophyta; class: Polypodiopsida; order: Polypodiales; family: Pteridaceae; genus: Pteris; specificEpithet: atrovirens; scientificNameAuthorship: Willd.; **Location:** continent: Africa; country: Togo; countryCode: TG; locality: Kpimé Séva; verbatimElevation: 496; verbatimSRS: WGS84; decimalLatitude: 7.008876; decimalLongitude: 0.640674; geodeticDatum: WGS84; **Identification:** identifiedBy: Abotsi, K.E.; dateIdentified: 08-04-16; **Event:** eventDate: 08-04-16; habitat: Rainforest; **Record Level:** institutionID: Herbarium Togoense; collectionID: Abotsi, K.E.; institutionCode: TOGO; basisOfRecord: Preserved specimen
- **Type status:** Other material. **Occurrence:** recordNumber: AB0291; recordedBy: Abotsi, K.E.; **Taxon:** scientificName: Pteris atrovirens Willd.; namePublishedIn: Sp. 5: 385 (1810); kingdom: Plantae; phylum: Pteridophyta; class: Polypodiopsida; order: Polypodiales; family: Pteridaceae; genus: Pteris; specificEpithet: atrovirens; scientificNameAuthorship: Willd.; **Location:** continent: Africa; country: Togo; countryCode: TG; locality: Sérégbéné; verbatimElevation: 523; verbatimSRS: WGS84; decimalLatitude: 7.864962; decimalLongitude: 0.739712; geodeticDatum: WGS84; **Identification:** identifiedBy: Abotsi, K.E.; dateIdentified: 09-17-16; **Event:** eventDate: 09-17-16; habitat: Rainforest; **Record Level:** institutionID: Herbarium Togoense; collectionID: Abotsi, K.E.; institutionCode: TOGO; basisOfRecord: Preserved specimen
- **Type status:** Other material. **Occurrence:** recordNumber: AB0318; recordedBy: Abotsi, K.E.; **Taxon:** scientificName: Pteris atrovirens Willd.; namePublishedIn: Sp. 5: 385 (1810); kingdom: Plantae; phylum: Pteridophyta; class: Polypodiopsida; order: Polypodiales; family: Pteridaceae; genus: Pteris; specificEpithet: atrovirens; scientificNameAuthorship: Willd.; **Location:** continent: Africa; country: Togo; countryCode: TG; locality: Assoukoko; verbatimElevation: 346; verbatimSRS: WGS84; decimalLatitude: 8.000469; decimalLongitude: 0.61531; geodeticDatum: WGS84; **Identification:** identifiedBy: Abotsi, K.E.; dateIdentified: 09-18-16; **Event:** eventDate: 09-18-16; habitat: Rainforest; **Record Level:** institutionID: Herbarium Togoense; collectionID: Abotsi, K.E.; institutionCode: TOGO; basisOfRecord: Preserved specimen

##### Ecological interactions

###### Native status

Native

##### Distribution

Zone 4

#### Pteris burtonii

Baker

##### Materials

- **Type status:** Other material. **Occurrence:** recordNumber: AB0107; recordedBy: Abotsi, K.E.; **Taxon:** scientificName: Pteris burtonii Baker; namePublishedIn: Ann. Bot. 5: 218 (1891); kingdom: Plantae; phylum: Pteridophyta; class: Polypodiopsida; order: Polypodiales; family: Pteridaceae; genus: Pteris; specificEpithet: burtonii; scientificNameAuthorship: Baker; **Location:** continent: Africa; country: Togo; countryCode: TG; locality: Danyi N'Digbé; verbatimElevation: 688; verbatimSRS: WGS84; decimalLatitude: 7.150935; decimalLongitude: 0.67243; geodeticDatum: WGS84; **Identification:** identifiedBy: Abotsi, K.E.; dateIdentified: 06-27-16; **Event:** eventDate: 06-27-16; habitat: Rainforest; **Record Level:** institutionID: Herbarium Togoense; collectionID: Abotsi, K.E.; institutionCode: TOGO; basisOfRecord: Preserved specimen
- **Type status:** Other material. **Occurrence:** recordNumber: AB0326; recordedBy: Abotsi, K.E.; **Taxon:** scientificName: Pteris burtonii Baker; namePublishedIn: Ann. Bot. 5: 218 (1891); kingdom: Plantae; phylum: Pteridophyta; class: Polypodiopsida; order: Polypodiales; family: Pteridaceae; genus: Pteris; specificEpithet: burtonii; scientificNameAuthorship: Baker; **Location:** continent: Africa; country: Togo; countryCode: TG; locality: Assoukoko; verbatimElevation: 342; verbatimSRS: WGS84; decimalLatitude: 8.00328; decimalLongitude: 0.61336; geodeticDatum: WGS84; **Identification:** identifiedBy: Abotsi, K.E.; dateIdentified: 09-18-16; **Event:** eventDate: 09-18-16; habitat: Rainforest; **Record Level:** institutionID: Herbarium Togoense; collectionID: Abotsi, K.E.; institutionCode: TOGO; basisOfRecord: Preserved specimen
- **Type status:** Other material. **Occurrence:** recordNumber: AB0415; recordedBy: Abotsi, K.E.; **Taxon:** scientificName: Pteris burtonii Baker; namePublishedIn: Ann. Bot. 5: 218 (1891); kingdom: Plantae; phylum: Pteridophyta; class: Polypodiopsida; order: Polypodiales; family: Pteridaceae; genus: Pteris; specificEpithet: burtonii; scientificNameAuthorship: Baker; **Location:** continent: Africa; country: Togo; countryCode: TG; locality: Danyi Dzogbégan; verbatimElevation: 712; verbatimSRS: WGS84; decimalLatitude: 7.232315; decimalLongitude: 0.677387; geodeticDatum: WGS84; **Identification:** identifiedBy: Abotsi, K.E.; dateIdentified: 12-27-16; **Event:** eventDate: 12-27-16; habitat: Rainforest; **Record Level:** institutionID: Herbarium Togoense; collectionID: Abotsi, K.E.; institutionCode: TOGO; basisOfRecord: Preserved specimen

##### Ecological interactions

###### Native status

Native

##### Distribution

Zone 4

#### Pteris cretica

L.

##### Materials

- **Type status:** Other material. **Occurrence:** recordedBy: Abotsi, K.E.; **Taxon:** scientificName: Pteris cretica L.; namePublishedIn: Mant. Pl. 130 (1767); kingdom: Plantae; phylum: Pteridophyta; class: Polypodiopsida; order: Polypodiales; family: Pteridaceae; genus: Pteris; specificEpithet: cretica; scientificNameAuthorship: L.; **Location:** continent: Africa; country: Togo; countryCode: TG; locality: Lomé, in gardens; **Identification:** identifiedBy: Abotsi, K.E.; **Event:** habitat: Gardens; **Record Level:** basisOfRecord: Human observation

##### Ecological interactions

###### Native status

Not native

##### Distribution

In gardens

#### Pteris ensiformis

Burm.

##### Materials

- **Type status:** Other material. **Occurrence:** recordedBy: Abotsi, K.E.; **Taxon:** scientificName: Pteris ensiformis Burm.; namePublishedIn: Fl. Ind. 230 (1768); kingdom: Plantae; phylum: Pteridophyta; class: Polypodiopsida; order: Polypodiales; family: Pteridaceae; genus: Pteris; specificEpithet: ensiformis; scientificNameAuthorship: Burm.; **Location:** continent: Africa; country: Togo; countryCode: TG; locality: Lomé, in gardens; **Identification:** identifiedBy: Abotsi, K.E.; **Event:** habitat: Gardens; **Record Level:** basisOfRecord: Human observation

##### Ecological interactions

###### Native status

Not native

##### Distribution

In gardens

#### Pteris hamulosa

(H. Christ) H. Christ

##### Materials

- **Type status:** Other material. **Occurrence:** recordNumber: AB0307; recordedBy: Abotsi, K.E.; **Taxon:** scientificName: Pteris hamulosa (H. Christ) H. Christ; namePublishedIn: Ann. Mus. Congo V, 8: 30 (1909); kingdom: Plantae; phylum: Pteridophyta; class: Polypodiopsida; order: Polypodiales; family: Pteridaceae; genus: Pteris; specificEpithet: hamulosa; scientificNameAuthorship: (H. Christ) H. Christ; **Location:** continent: Africa; country: Togo; countryCode: TG; locality: Assoukoko; verbatimElevation: 586; verbatimSRS: WGS84; decimalLatitude: 8.010176; decimalLongitude: 0.656913; geodeticDatum: WGS84; **Identification:** identifiedBy: Abotsi, K.E.; dateIdentified: 09-17-16; **Event:** eventDate: 09-17-16; habitat: Rainforest; **Record Level:** institutionID: Herbarium Togoense; collectionID: Abotsi, K.E.; institutionCode: TOGO; basisOfRecord: Preserved specimen
- **Type status:** Other material. **Occurrence:** recordNumber: AB0379; recordedBy: Abotsi, K.E.; **Taxon:** scientificName: Pteris hamulosa (H. Christ) H. Christ; namePublishedIn: Ann. Mus. Congo V, 8: 30 (1909); kingdom: Plantae; phylum: Pteridophyta; class: Polypodiopsida; order: Polypodiales; family: Pteridaceae; genus: Pteris; specificEpithet: hamulosa; scientificNameAuthorship: (H. Christ) H. Christ; **Location:** continent: Africa; country: Togo; countryCode: TG; locality: Lalamila; verbatimElevation: 573; verbatimSRS: WGS84; decimalLatitude: 8.164824; decimalLongitude: 0.807288; geodeticDatum: WGS84; **Identification:** identifiedBy: Abotsi, K.E.; dateIdentified: 10-05-16; **Event:** eventDate: 10-05-16; habitat: Rainforest; **Record Level:** institutionID: Herbarium Togoense; collectionID: Abotsi, K.E.; institutionCode: TOGO; basisOfRecord: Preserved specimen
- **Type status:** Other material. **Occurrence:** recordNumber: AB0449; recordedBy: Abotsi, K.E.; **Taxon:** scientificName: Pteris hamulosa (H. Christ) H. Christ; namePublishedIn: Ann. Mus. Congo V, 8: 30 (1909); kingdom: Plantae; phylum: Pteridophyta; class: Polypodiopsida; order: Polypodiales; family: Pteridaceae; genus: Pteris; specificEpithet: hamulosa; scientificNameAuthorship: (H. Christ) H. Christ; **Location:** continent: Africa; country: Togo; countryCode: TG; locality: Agomé Yoh; verbatimElevation: 346; verbatimSRS: WGS84; decimalLatitude: 6.948842; decimalLongitude: 0.597537; geodeticDatum: WGS84; **Identification:** identifiedBy: Abotsi, K.E.; dateIdentified: 12-29-16; **Event:** eventDate: 12-29-16; habitat: Coffee/cocoa based agroforest; **Record Level:** institutionID: Herbarium Togoense; collectionID: Abotsi, K.E.; institutionCode: TOGO; basisOfRecord: Preserved specimen

##### Ecological interactions

###### Native status

Native

##### Distribution

Zone 4

#### Pteris linearis

Poir.

##### Materials

- **Type status:** Other material. **Occurrence:** recordNumber: AB0287; recordedBy: Abotsi, K.E.; **Taxon:** scientificName: Pteris linearis Poir.; namePublishedIn: Encycl. 5: 723 (1804); kingdom: Plantae; phylum: Pteridophyta; class: Polypodiopsida; order: Polypodiales; family: Pteridaceae; genus: Pteris; specificEpithet: linearis; scientificNameAuthorship: Poir.; **Location:** continent: Africa; country: Togo; countryCode: TG; locality: Sérégbéné; verbatimElevation: 527; verbatimSRS: WGS84; decimalLatitude: 7.865099; decimalLongitude: 0.739534; geodeticDatum: WGS84; **Identification:** identifiedBy: Abotsi, K.E.; dateIdentified: 09-17-16; **Event:** eventDate: 09-17-16; habitat: Rainforest; **Record Level:** institutionID: Herbarium Togoense; collectionID: Abotsi, K.E.; institutionCode: TOGO; basisOfRecord: Preserved specimen
- **Type status:** Other material. **Occurrence:** recordNumber: AB0299; recordedBy: Abotsi, K.E.; **Taxon:** scientificName: Pteris linearis Poir.; namePublishedIn: Encycl. 5: 723 (1804); kingdom: Plantae; phylum: Pteridophyta; class: Polypodiopsida; order: Polypodiales; family: Pteridaceae; genus: Pteris; specificEpithet: linearis; scientificNameAuthorship: Poir.; **Location:** continent: Africa; country: Togo; countryCode: TG; locality: Sakoundè; verbatimElevation: 417; verbatimSRS: WGS84; decimalLatitude: 7.868141; decimalLongitude: 0.657472; geodeticDatum: WGS84; **Identification:** identifiedBy: Abotsi, K.E.; dateIdentified: 09-17-16; **Event:** eventDate: 09-17-16; habitat: Rainforest; **Record Level:** institutionID: Herbarium Togoense; collectionID: Abotsi, K.E.; institutionCode: TOGO; basisOfRecord: Preserved specimen
- **Type status:** Other material. **Occurrence:** recordNumber: AB0347; recordedBy: Abotsi, K.E.; **Taxon:** scientificName: Pteris linearis Poir.; namePublishedIn: Encycl. 5: 723 (1804); kingdom: Plantae; phylum: Pteridophyta; class: Polypodiopsida; order: Polypodiales; family: Pteridaceae; genus: Pteris; specificEpithet: linearis; scientificNameAuthorship: Poir.; **Location:** continent: Africa; country: Togo; countryCode: TG; locality: Danyi Gbaladzé; verbatimElevation: 821; verbatimSRS: WGS84; decimalLatitude: 7.210018; decimalLongitude: 0.732149; geodeticDatum: WGS84; **Identification:** identifiedBy: Abotsi, K.E.; dateIdentified: 10-01-16; **Event:** eventDate: 10-01-16; habitat: Coffee/cocoa based agroforest; **Record Level:** institutionID: Herbarium Togoense; collectionID: Abotsi, K.E.; institutionCode: TOGO; basisOfRecord: Preserved specimen

##### Ecological interactions

###### Native status

Native

##### Distribution

Zone 4

#### Pteris mildbraedii

Hieron.

##### Materials

- **Type status:** Other material. **Occurrence:** recordNumber: AB0342; recordedBy: Abotsi, K.E.; **Taxon:** scientificName: Pteris mildbraedii Hieron.; namePublishedIn: Engl. Jahrb. 68: 415 (1915); kingdom: Plantae; phylum: Pteridophyta; class: Polypodiopsida; order: Polypodiales; family: Pteridaceae; genus: Pteris; specificEpithet: mildbraedii; scientificNameAuthorship: Hieron.; **Location:** continent: Africa; country: Togo; countryCode: TG; locality: Danyi Kounsountou; verbatimElevation: 723; verbatimSRS: WGS84; decimalLatitude: 7.210024; decimalLongitude: 0.636543; geodeticDatum: WGS84; **Identification:** identifiedBy: Abotsi, K.E.; dateIdentified: 09-30-16; **Event:** eventDate: 09-30-16; habitat: Coffee/cocoa based agroforest; **Record Level:** institutionID: Herbarium Togoense; collectionID: Abotsi, K.E.; institutionCode: TOGO; basisOfRecord: Preserved specimen
- **Type status:** Other material. **Occurrence:** recordNumber: AB0062; recordedBy: Abotsi, K.E.; **Taxon:** scientificName: Pteris mildbraedii Hieron.; namePublishedIn: Engl. Jahrb. 68: 415 (1915); kingdom: Plantae; phylum: Pteridophyta; class: Polypodiopsida; order: Polypodiales; family: Pteridaceae; genus: Pteris; specificEpithet: mildbraedii; scientificNameAuthorship: Hieron.; **Location:** continent: Africa; country: Togo; countryCode: TG; locality: Missahoé; verbatimElevation: 527; verbatimSRS: WGS84; decimalLatitude: 6.946945; decimalLongitude: 0.580444; geodeticDatum: WGS84; **Identification:** identifiedBy: Abotsi, K.E.; dateIdentified: 06-24-16; **Event:** eventDate: 06-24-16; habitat: Rainforest; **Record Level:** institutionID: Herbarium Togoense; collectionID: Abotsi, K.E.; institutionCode: TOGO; basisOfRecord: Preserved specimen
- **Type status:** Other material. **Occurrence:** recordNumber: AB0510; recordedBy: Abotsi, K.E.; **Taxon:** scientificName: Pteris mildbraedii Hieron.; namePublishedIn: Engl. Jahrb. 68: 415 (1915); kingdom: Plantae; phylum: Pteridophyta; class: Polypodiopsida; order: Polypodiales; family: Pteridaceae; genus: Pteris; specificEpithet: mildbraedii; scientificNameAuthorship: Hieron.; **Location:** continent: Africa; country: Togo; countryCode: TG; locality: Gbadi Gawodo; verbatimElevation: 639; verbatimSRS: WGS84; decimalLatitude: 7.484727; decimalLongitude: 0.766313; geodeticDatum: WGS84; **Identification:** identifiedBy: Abotsi, K.E.; dateIdentified: 04-26-17; **Event:** eventDate: 04-26-17; habitat: Rainforest; **Record Level:** institutionID: Herbarium Togoense; collectionID: Abotsi, K.E.; institutionCode: TOGO; basisOfRecord: Preserved specimen

##### Ecological interactions

###### Native status

Native

##### Distribution

Zone 4

#### Pteris similis

Kuhn.

##### Materials

- **Type status:** Other material. **Occurrence:** recordNumber: AB0375; recordedBy: Abotsi, K.E.; **Taxon:** scientificName: Pteris similis Kuhn.; namePublishedIn: v. Decken, Reis. 3(3), Bot. 21 (1879); kingdom: Plantae; phylum: Pteridophyta; class: Polypodiopsida; order: Polypodiales; family: Pteridaceae; genus: Pteris; specificEpithet: similis; scientificNameAuthorship: Kuhn.; **Location:** continent: Africa; country: Togo; countryCode: TG; locality: Katchenké; verbatimElevation: 619; verbatimSRS: WGS84; decimalLatitude: 8.196816; decimalLongitude: 0.682541; geodeticDatum: WGS84; **Identification:** identifiedBy: Abotsi, K.E.; dateIdentified: 10-05-16; **Event:** eventDate: 10-05-16; habitat: Rainforest; **Record Level:** institutionID: Herbarium Togoense; collectionID: Abotsi, K.E.; institutionCode: TOGO; basisOfRecord: Preserved specimen
- **Type status:** Other material. **Occurrence:** recordNumber: AB0410; recordedBy: Abotsi, K.E.; **Taxon:** scientificName: Pteris similis Kuhn.; namePublishedIn: v. Decken, Reis. 3(3), Bot. 21 (1879); kingdom: Plantae; phylum: Pteridophyta; class: Polypodiopsida; order: Polypodiales; family: Pteridaceae; genus: Pteris; specificEpithet: similis; scientificNameAuthorship: Kuhn.; **Location:** continent: Africa; country: Togo; countryCode: TG; locality: Danyi Apéyémé; verbatimElevation: 689; verbatimSRS: WGS84; decimalLatitude: 7.184642; decimalLongitude: 0.692076; geodeticDatum: WGS84; **Identification:** identifiedBy: Abotsi, K.E.; dateIdentified: 12-27-16; **Event:** eventDate: 12-27-16; habitat: Rainforest; **Record Level:** institutionID: Herbarium Togoense; collectionID: Abotsi, K.E.; institutionCode: TOGO; basisOfRecord: Preserved specimen
- **Type status:** Other material. **Occurrence:** recordNumber: AB0496; recordedBy: Abotsi, K.E.; **Taxon:** scientificName: Pteris similis Kuhn.; namePublishedIn: v. Decken, Reis. 3(3), Bot. 21 (1879); kingdom: Plantae; phylum: Pteridophyta; class: Polypodiopsida; order: Polypodiales; family: Pteridaceae; genus: Pteris; specificEpithet: similis; scientificNameAuthorship: Kuhn.; **Location:** continent: Africa; country: Togo; countryCode: TG; locality: Agbo Kopé; verbatimElevation: 669; verbatimSRS: WGS84; decimalLatitude: 7.454767; decimalLongitude: 0.676454; geodeticDatum: WGS84; **Identification:** identifiedBy: Abotsi, K.E.; dateIdentified: 04-25-17; **Event:** eventDate: 04-25-17; habitat: Rainforest; **Record Level:** institutionID: Herbarium Togoense; collectionID: Abotsi, K.E.; institutionCode: TOGO; basisOfRecord: Preserved specimen

##### Ecological interactions

###### Native status

Native

##### Distribution

Zone 4

#### Pteris togoensis

Hieron.

##### Materials

- **Type status:** Other material. **Occurrence:** recordNumber: AB0213; recordedBy: Abotsi, K.E.; **Taxon:** scientificName: Pteris togoensis Hieron.; namePublishedIn: Engl. Jahrb. 68: 402 (1915); kingdom: Plantae; phylum: Pteridophyta; class: Polypodiopsida; order: Polypodiales; family: Pteridaceae; genus: Pteris; specificEpithet: togoensis; scientificNameAuthorship: Hieron.; **Location:** continent: Africa; country: Togo; countryCode: TG; locality: Blifou; verbatimElevation: 773; verbatimSRS: WGS84; decimalLatitude: 7.022064; decimalLongitude: 0.612798; geodeticDatum: WGS84; **Identification:** identifiedBy: Abotsi, K.E.; dateIdentified: 08-04-16; **Event:** eventDate: 08-04-16; habitat: Rainforest; **Record Level:** institutionID: Herbarium Togoense; collectionID: Abotsi, K.E.; institutionCode: TOGO; basisOfRecord: Preserved specimen
- **Type status:** Other material. **Occurrence:** recordNumber: AB0381; recordedBy: Abotsi, K.E.; **Taxon:** scientificName: Pteris togoensis Hieron.; namePublishedIn: Engl. Jahrb. 68: 402 (1915); kingdom: Plantae; phylum: Pteridophyta; class: Polypodiopsida; order: Polypodiales; family: Pteridaceae; genus: Pteris; specificEpithet: togoensis; scientificNameAuthorship: Hieron.; **Location:** continent: Africa; country: Togo; countryCode: TG; locality: Yégué; verbatimElevation: 495; verbatimSRS: WGS84; decimalLatitude: 8.150762; decimalLongitude: 0.646497; geodeticDatum: WGS84; **Identification:** identifiedBy: Abotsi, K.E.; dateIdentified: 10-06-16; **Event:** eventDate: 10-06-16; habitat: Wooded savannah; **Record Level:** institutionID: Herbarium Togoense; collectionID: Abotsi, K.E.; institutionCode: TOGO; basisOfRecord: Preserved specimen
- **Type status:** Other material. **Occurrence:** recordNumber: AB0580; recordedBy: Abotsi, K.E.; **Taxon:** scientificName: Pteris togoensis Hieron.; namePublishedIn: Engl. Jahrb. 68: 402 (1915); kingdom: Plantae; phylum: Pteridophyta; class: Polypodiopsida; order: Polypodiales; family: Pteridaceae; genus: Pteris; specificEpithet: togoensis; scientificNameAuthorship: Hieron.; **Location:** continent: Africa; country: Togo; countryCode: TG; locality: Souroukou; verbatimElevation: 322; verbatimSRS: WGS84; decimalLatitude: 8.756245; decimalLongitude: 0.680874; geodeticDatum: WGS84; **Identification:** identifiedBy: Abotsi, K.E.; dateIdentified: 07-15-17; **Event:** eventDate: 07-15-17; habitat: Dry dense forest; **Record Level:** institutionID: Herbarium Togoense; collectionID: Abotsi, K.E.; institutionCode: TOGO; basisOfRecord: Preserved specimen

##### Ecological interactions

###### Native status

Native

##### Distribution

Zones 2 and 4

#### Pteris tripartita

Sw.

##### Materials

- **Type status:** Other material. **Occurrence:** recordNumber: ASM 0118; recordedBy: Abotsi, K.E.; **Taxon:** scientificName: Pteris tripartita Sw.; namePublishedIn: Schrad., J. Bot. 1800(2): 67 (1801); kingdom: Plantae; phylum: Pteridophyta; class: Polypodiopsida; order: Polypodiales; family: Pteridaceae; genus: Pteris; specificEpithet: tripartita; scientificNameAuthorship: Sw.; **Location:** continent: Africa; country: Togo; countryCode: TG; locality: Badou, RNET; verbatimElevation: 333; verbatimSRS: WGS84; decimalLatitude: 7.582054548; decimalLongitude: 0.621264783; geodeticDatum: WGS84; **Identification:** identifiedBy: Abotsi, K.E.; dateIdentified: 04-05-13; **Event:** eventDate: 04-05-13; habitat: Rainforest; **Record Level:** institutionID: Herbarium Togoense; collectionID: Abotsi, K.E.; institutionCode: TOGO; basisOfRecord: Preserved specimen
- **Type status:** Other material. **Occurrence:** recordNumber: AB0612; recordedBy: Abotsi, K.E.; **Taxon:** scientificName: Pteris tripartita Sw.; namePublishedIn: Schrad., J. Bot. 1800(2): 67 (1801); kingdom: Plantae; phylum: Pteridophyta; class: Polypodiopsida; order: Polypodiales; family: Pteridaceae; genus: Pteris; specificEpithet: tripartita; scientificNameAuthorship: Sw.; **Location:** continent: Africa; country: Togo; countryCode: TG; locality: Womé, near the waterfalls; verbatimElevation: 299; verbatimSRS: WGS84; decimalLatitude: 6.857523; decimalLongitude: 0.55423; geodeticDatum: WGS84; **Identification:** identifiedBy: Abotsi, K.E.; dateIdentified: 01-21-18; **Event:** eventDate: 01-21-18; habitat: Rainforest; **Record Level:** institutionID: Herbarium Togoense; collectionID: Abotsi, K.E.; institutionCode: TOGO; basisOfRecord: Preserved specimen

##### Ecological interactions

###### Native status

Native

##### Distribution

Zone 4

#### Vittarioideae

#### Adiantum comoroense

(Tard.) Verdc.

##### Materials

- **Type status:** Other material. **Occurrence:** recordedBy: Roux, J.P.; **Taxon:** scientificName: Adiantum comoroense (Tard.) Verdc.; namePublishedIn: Kew Bull. 46(2): 272 (1991); kingdom: Plantae; phylum: Pteridophyta; class: Polypodiopsida; order: Polypodiales; family: Pteridaceae; genus: Adiantum; specificEpithet: comoroense; scientificNameAuthorship: (Tard.) Verdc.; **Location:** continent: Africa; country: Togo; countryCode: TG; **Event:** habitat: Rainforest; **Record Level:** basisOfRecord: Unknown; source: Synopsis of the Lycopodiophyta and Pteridophyta of Africa, Madagascar and neighbouring islands (Roux, 2009)

##### Ecological interactions

###### Native status

Native

##### Distribution

Uncertain

#### Adiantum confine

Fée.

##### Materials

- **Type status:** Other material. **Occurrence:** recordNumber: AB0007; recordedBy: Abotsi, K.E.; **Taxon:** scientificName: Adiantum confine Fée.; namePublishedIn: Mémoires de la Société des Sciences Naturelles de Strasbourg 6, 1: 14, t. 32, fig. 1 (1866); kingdom: Plantae; phylum: Pteridophyta; class: Polypodiopsida; order: Polypodiales; family: Pteridaceae; genus: Adiantum; specificEpithet: confine; scientificNameAuthorship: Fée.; **Location:** continent: Africa; country: Togo; countryCode: TG; locality: Agou Nyogbo; verbatimElevation: 410; verbatimSRS: WGS84; decimalLatitude: 6.878968; decimalLongitude: 0.730342; geodeticDatum: WGS84; **Identification:** identifiedBy: Abotsi, K.E.; dateIdentified: 06-13-16; **Event:** eventDate: 06-13-16; habitat: Coffee/cocoa based agroforest; **Record Level:** institutionID: Herbarium Togoense; collectionID: Abotsi, K.E.; institutionCode: TOGO; basisOfRecord: Preserved specimen
- **Type status:** Other material. **Occurrence:** recordNumber: AB0011; recordedBy: Abotsi, K.E.; **Taxon:** scientificName: Adiantum confine Fée.; namePublishedIn: Mémoires de la Société des Sciences Naturelles de Strasbourg 6, 1: 14, t. 32, fig. 1 (1866); kingdom: Plantae; phylum: Pteridophyta; class: Polypodiopsida; order: Polypodiales; family: Pteridaceae; genus: Adiantum; specificEpithet: confine; scientificNameAuthorship: Fée.; **Location:** continent: Africa; country: Togo; countryCode: TG; locality: Agou Nyogbo; verbatimElevation: 547; verbatimSRS: WGS84; decimalLatitude: 6.87801; decimalLongitude: 0.733795; geodeticDatum: WGS84; **Identification:** identifiedBy: Abotsi, K.E.; dateIdentified: 06-13-16; **Event:** eventDate: 06-13-16; habitat: Coffee/cocoa based agroforest; **Record Level:** institutionID: Herbarium Togoense; collectionID: Abotsi, K.E.; institutionCode: TOGO; basisOfRecord: Preserved specimen
- **Type status:** Other material. **Occurrence:** recordNumber: AB0164; recordedBy: Abotsi, K.E.; **Taxon:** scientificName: Adiantum confine Fée.; namePublishedIn: Mémoires de la Société des Sciences Naturelles de Strasbourg 6, 1: 14, t. 32, fig. 1 (1866); kingdom: Plantae; phylum: Pteridophyta; class: Polypodiopsida; order: Polypodiales; family: Pteridaceae; genus: Adiantum; specificEpithet: confine; scientificNameAuthorship: Fée.; **Location:** continent: Africa; country: Togo; countryCode: TG; locality: Kpimé Séva; verbatimElevation: 301; verbatimSRS: WGS84; decimalLatitude: 7.006426; decimalLongitude: 0.646555; geodeticDatum: WGS84; **Identification:** identifiedBy: Abotsi, K.E.; dateIdentified: 07-23-16; **Event:** eventDate: 07-23-16; habitat: Rainforest; **Record Level:** institutionID: Herbarium Togoense; collectionID: Abotsi, K.E.; institutionCode: TOGO; basisOfRecord: Preserved specimen

##### Ecological interactions

###### Native status

Native

##### Distribution

Zone 4

#### Adiantum incisum

Forsk.

##### Materials

- **Type status:** Other material. **Occurrence:** recordNumber: ASM 0306; recordedBy: Abotsi, K.E., Sodjinou E. & Mingou P.; **Taxon:** scientificName: Adiantum incisum Forsk.; namePublishedIn: Fl. Aeg.-Arab. 187 (1775); kingdom: Plantae; phylum: Pteridophyta; class: Polypodiopsida; order: Polypodiales; family: Pteridaceae; genus: Adiantum; specificEpithet: incisum; scientificNameAuthorship: Forsk.; **Location:** continent: Africa; country: Togo; countryCode: TG; locality: Mt Agou, Dalavé; verbatimElevation: 283; verbatimSRS: WGS84; decimalLatitude: 6.846671827; decimalLongitude: 0.748630421; geodeticDatum: WGS84; **Identification:** identifiedBy: Abotsi, K.E.; dateIdentified: 04-16-13; **Event:** eventDate: 04-16-13; habitat: Rainforest; **Record Level:** institutionID: Herbarium Togoense; collectionID: Abotsi, K.E.; institutionCode: TOGO; basisOfRecord: Preserved specimen
- **Type status:** Other material. **Occurrence:** recordNumber: AB0360; recordedBy: Abotsi, K.E.; **Taxon:** scientificName: Adiantum incisum Forsk.; namePublishedIn: Fl. Aeg.-Arab. 187 (1775); kingdom: Plantae; phylum: Pteridophyta; class: Polypodiopsida; order: Polypodiales; family: Pteridaceae; genus: Adiantum; specificEpithet: incisum; scientificNameAuthorship: Forsk.; **Location:** continent: Africa; country: Togo; countryCode: TG; locality: Hounto; verbatimElevation: 217; verbatimSRS: WGS84; decimalLatitude: 7.684263; decimalLongitude: 1.46475; geodeticDatum: WGS84; **Identification:** identifiedBy: Abotsi, K.E.; dateIdentified: 10-04-16; **Event:** eventDate: 10-04-16; habitat: Wooded savannah; **Record Level:** institutionID: Herbarium Togoense; collectionID: Abotsi, K.E.; institutionCode: TOGO; basisOfRecord: Preserved specimen
- **Type status:** Other material. **Occurrence:** recordNumber: ASM 0351; recordedBy: Abotsi, K.E., Sodjinou E. & Mingou P.; **Taxon:** scientificName: Adiantum incisum Forsk.; namePublishedIn: Fl. Aeg.-Arab. 187 (1775); kingdom: Plantae; phylum: Pteridophyta; class: Polypodiopsida; order: Polypodiales; family: Pteridaceae; genus: Adiantum; specificEpithet: incisum; scientificNameAuthorship: Forsk.; **Location:** continent: Africa; country: Togo; countryCode: TG; locality: Tomégbé; verbatimElevation: 254; verbatimSRS: WGS84; decimalLatitude: 7.511856703; decimalLongitude: 0.601633983; geodeticDatum: WGS84; **Identification:** identifiedBy: Abotsi, K.E.; dateIdentified: 04-03-14; **Event:** eventDate: 04-03-14; habitat: Inhabited area, on a wall; **Record Level:** institutionID: Herbarium Togoense; collectionID: Abotsi, K.E.; institutionCode: TOGO; basisOfRecord: Preserved specimen

##### Ecological interactions

###### Native status

Native

##### Distribution

Zones 3 and 4

#### Adiantum philippense

L.

##### Materials

- **Type status:** Other material. **Occurrence:** recordNumber: AB0221; recordedBy: Abotsi, K.E.; **Taxon:** scientificName: Adiantum philippense L.; namePublishedIn: Sp. 2: 1094 (1753); kingdom: Plantae; phylum: Pteridophyta; class: Polypodiopsida; order: Polypodiales; family: Pteridaceae; genus: Adiantum; specificEpithet: philippense; scientificNameAuthorship: L.; **Location:** continent: Africa; country: Togo; countryCode: TG; locality: Ounabè; verbatimElevation: 506; verbatimSRS: WGS84; decimalLatitude: 7.560316; decimalLongitude: 1.019679; geodeticDatum: WGS84; **Identification:** identifiedBy: Abotsi, K.E.; dateIdentified: 08-05-16; **Event:** eventDate: 08-05-16; habitat: Rainforest; **Record Level:** institutionID: Herbarium Togoense; collectionID: Abotsi, K.E.; institutionCode: TOGO; basisOfRecord: Preserved specimen
- **Type status:** Other material. **Occurrence:** recordNumber: AB0263; recordedBy: Abotsi, K.E.; **Taxon:** scientificName: Adiantum philippense L.; namePublishedIn: Sp. 2: 1094 (1753); kingdom: Plantae; phylum: Pteridophyta; class: Polypodiopsida; order: Polypodiales; family: Pteridaceae; genus: Adiantum; specificEpithet: philippense; scientificNameAuthorship: L.; **Location:** continent: Africa; country: Togo; countryCode: TG; locality: Badou; verbatimElevation: 401; verbatimSRS: WGS84; decimalLatitude: 7.580778; decimalLongitude: 0.622699; geodeticDatum: WGS84; **Identification:** identifiedBy: Abotsi, K.E.; dateIdentified: 08-08-16; **Event:** eventDate: 08-08-16; habitat: Rainforest; **Record Level:** institutionID: Herbarium Togoense; collectionID: Abotsi, K.E.; institutionCode: TOGO; basisOfRecord: Preserved specimen
- **Type status:** Other material. **Occurrence:** recordNumber: AB0288; recordedBy: Abotsi, K.E.; **Taxon:** scientificName: Adiantum philippense L.; namePublishedIn: Sp. 2: 1094 (1753); kingdom: Plantae; phylum: Pteridophyta; class: Polypodiopsida; order: Polypodiales; family: Pteridaceae; genus: Adiantum; specificEpithet: philippense; scientificNameAuthorship: L.; **Location:** continent: Africa; country: Togo; countryCode: TG; locality: Sérégbéné; verbatimElevation: 528; verbatimSRS: WGS84; decimalLatitude: 7.865086; decimalLongitude: 0.739546; geodeticDatum: WGS84; **Identification:** identifiedBy: Abotsi, K.E.; dateIdentified: 09-17-16; **Event:** eventDate: 09-17-16; habitat: Rainforest; **Record Level:** institutionID: Herbarium Togoense; collectionID: Abotsi, K.E.; institutionCode: TOGO; basisOfRecord: Preserved specimen

##### Ecological interactions

###### Native status

Native

##### Distribution

Zones 2, 3 and 4

#### Adiantum schweinfurthii

Kuhn.

##### Materials

- **Type status:** Other material. **Occurrence:** recordNumber: AB0311; recordedBy: Abotsi, K.E.; **Taxon:** scientificName: Adiantum schweinfurthii Kuhn.; namePublishedIn: Sitz. Ges. Naturf. Freunde Berl. 40: 40 (1869); kingdom: Plantae; phylum: Pteridophyta; class: Polypodiopsida; order: Polypodiales; family: Pteridaceae; genus: Adiantum; specificEpithet: schweinfurthii; scientificNameAuthorship: Kuhn.; **Location:** continent: Africa; country: Togo; countryCode: TG; locality: Assoukoko; verbatimElevation: 631; verbatimSRS: WGS84; decimalLatitude: 8.005067; decimalLongitude: 0.627456; geodeticDatum: WGS84; **Identification:** identifiedBy: Abotsi, K.E.; dateIdentified: 09-18-16; **Event:** eventDate: 09-18-16; habitat: Rainforest; **Record Level:** institutionID: Herbarium Togoense; collectionID: Abotsi, K.E.; institutionCode: TOGO; basisOfRecord: Preserved specimen
- **Type status:** Other material. **Occurrence:** recordNumber: AB0370; recordedBy: Abotsi, K.E.; **Taxon:** scientificName: Adiantum schweinfurthii Kuhn.; namePublishedIn: Sitz. Ges. Naturf. Freunde Berl. 40: 40 (1869); kingdom: Plantae; phylum: Pteridophyta; class: Polypodiopsida; order: Polypodiales; family: Pteridaceae; genus: Adiantum; specificEpithet: schweinfurthii; scientificNameAuthorship: Kuhn.; **Location:** continent: Africa; country: Togo; countryCode: TG; locality: Dikpéléou; verbatimElevation: 717; verbatimSRS: WGS84; decimalLatitude: 8.202439; decimalLongitude: 0.614869; geodeticDatum: WGS84; **Identification:** identifiedBy: Abotsi, K.E.; dateIdentified: 10-05-16; **Event:** eventDate: 10-05-16; habitat: Rainforest; **Record Level:** institutionID: Herbarium Togoense; collectionID: Abotsi, K.E.; institutionCode: TOGO; basisOfRecord: Preserved specimen
- **Type status:** Other material. **Occurrence:** recordNumber: AB0591; recordedBy: Abotsi, K.E.; **Taxon:** scientificName: Adiantum schweinfurthii Kuhn.; namePublishedIn: Sitz. Ges. Naturf. Freunde Berl. 40: 40 (1869); kingdom: Plantae; phylum: Pteridophyta; class: Polypodiopsida; order: Polypodiales; family: Pteridaceae; genus: Adiantum; specificEpithet: schweinfurthii; scientificNameAuthorship: Kuhn.; **Location:** continent: Africa; country: Togo; countryCode: TG; locality: Fazao; verbatimElevation: 537; verbatimSRS: WGS84; decimalLatitude: 8.626659; decimalLongitude: 0.762768; geodeticDatum: WGS84; **Identification:** identifiedBy: Abotsi, K.E.; dateIdentified: 07-17-17; **Event:** eventDate: 07-17-17; habitat: Dry dense forest; **Record Level:** institutionID: Herbarium Togoense; collectionID: Abotsi, K.E.; institutionCode: TOGO; basisOfRecord: Preserved specimen

##### Ecological interactions

###### Native status

Native

##### Distribution

Zones 2 and 4

#### Adiantum soboliferum

Wall.

##### Materials

- **Type status:** Other material. **Occurrence:** recordNumber: AB0253; recordedBy: Abotsi, K.E.; **Taxon:** scientificName: Adiantum soboliferum Wall.; namePublishedIn: Cat. n. 74 (1828) ex Hook., Sp. Fil. 2: 13, t. 74 A (1851); kingdom: Plantae; phylum: Pteridophyta; class: Polypodiopsida; order: Polypodiales; family: Pteridaceae; genus: Adiantum; specificEpithet: soboliferum; scientificNameAuthorship: Wall.; **Location:** continent: Africa; country: Togo; countryCode: TG; locality: Kessibo Wawa; verbatimElevation: 193; verbatimSRS: WGS84; decimalLatitude: 7.699482; decimalLongitude: 0.59182; geodeticDatum: WGS84; **Identification:** identifiedBy: Abotsi, K.E.; dateIdentified: 08-07-16; **Event:** eventDate: 08-07-16; habitat: Coffee/cocoa based agroforest; **Record Level:** institutionID: Herbarium Togoense; collectionID: Abotsi, K.E.; institutionCode: TOGO; basisOfRecord: Preserved specimen
- **Type status:** Other material. **Occurrence:** recordNumber: AB0257; recordedBy: Abotsi, K.E.; **Taxon:** scientificName: Adiantum soboliferum Wall.; namePublishedIn: Cat. n. 74 (1828) ex Hook., Sp. Fil. 2: 13, t. 74 A (1851); kingdom: Plantae; phylum: Pteridophyta; class: Polypodiopsida; order: Polypodiales; family: Pteridaceae; genus: Adiantum; specificEpithet: soboliferum; scientificNameAuthorship: Wall.; **Location:** continent: Africa; country: Togo; countryCode: TG; locality: Badou; verbatimElevation: 314; verbatimSRS: WGS84; decimalLatitude: 7.581881; decimalLongitude: 0.621186; geodeticDatum: WGS84; **Identification:** identifiedBy: Abotsi, K.E.; dateIdentified: 08-08-16; **Event:** eventDate: 08-08-16; habitat: Rainforest; **Record Level:** institutionID: Herbarium Togoense; collectionID: Abotsi, K.E.; institutionCode: TOGO; basisOfRecord: Preserved specimen
- **Type status:** Other material. **Occurrence:** recordNumber: AB0552; recordedBy: Abotsi, K.E.; **Taxon:** scientificName: Adiantum soboliferum Wall.; namePublishedIn: Cat. n. 74 (1828) ex Hook., Sp. Fil. 2: 13, t. 74 A (1851); kingdom: Plantae; phylum: Pteridophyta; class: Polypodiopsida; order: Polypodiales; family: Pteridaceae; genus: Adiantum; specificEpithet: soboliferum; scientificNameAuthorship: Wall.; **Location:** continent: Africa; country: Togo; countryCode: TG; locality: Alédjo; verbatimElevation: 517; verbatimSRS: WGS84; decimalLatitude: 9.272343; decimalLongitude: 1.207248; geodeticDatum: WGS84; **Identification:** identifiedBy: Abotsi, K.E.; dateIdentified: 07-12-17; **Event:** eventDate: 07-12-17; habitat: Dry dense forest; **Record Level:** institutionID: Herbarium Togoense; collectionID: Abotsi, K.E.; institutionCode: TOGO; basisOfRecord: Preserved specimen

##### Ecological interactions

###### Native status

Native

##### Distribution

Zones 2 and 4

#### Adiantum vogelii

Mett. ex Kuhn.

##### Materials

- **Type status:** Other material. **Occurrence:** recordNumber: AB0141; recordedBy: Abotsi, K.E.; **Taxon:** scientificName: Adiantum vogelii Mett. ex Kuhn.; namePublishedIn: Fil. Afr. 66 (1868), syn., ex Keys., Mém. Ac. Imp. Sci. St. Pétersb. 22(1): 8, 31 (1875); kingdom: Plantae; phylum: Pteridophyta; class: Polypodiopsida; order: Polypodiales; family: Pteridaceae; genus: Adiantum; specificEpithet: vogelii; scientificNameAuthorship: Mett. ex Kuhn.; **Location:** continent: Africa; country: Togo; countryCode: TG; locality: Danyi N'Digbé; verbatimElevation: 572; verbatimSRS: WGS84; decimalLatitude: 7.122562; decimalLongitude: 0.654219; geodeticDatum: WGS84; **Identification:** identifiedBy: Abotsi, K.E.; dateIdentified: 06-28-16; **Event:** eventDate: 06-28-16; habitat: Coffee/cocoa based agroforest; **Record Level:** institutionID: Herbarium Togoense; collectionID: Abotsi, K.E.; institutionCode: TOGO; basisOfRecord: Preserved specimen
- **Type status:** Other material. **Occurrence:** recordNumber: AB0193; recordedBy: Abotsi, K.E.; **Taxon:** scientificName: Adiantum vogelii Mett. ex Kuhn.; namePublishedIn: Fil. Afr. 66 (1868), syn., ex Keys., Mém. Ac. Imp. Sci. St. Pétersb. 22(1): 8, 31 (1875); kingdom: Plantae; phylum: Pteridophyta; class: Polypodiopsida; order: Polypodiales; family: Pteridaceae; genus: Adiantum; specificEpithet: vogelii; scientificNameAuthorship: Mett. ex Kuhn.; **Location:** continent: Africa; country: Togo; countryCode: TG; locality: Womé; verbatimElevation: 338; verbatimSRS: WGS84; decimalLatitude: 6.858295; decimalLongitude: 0.553963; geodeticDatum: WGS84; **Identification:** identifiedBy: Abotsi, K.E.; dateIdentified: 08-03-16; **Event:** eventDate: 08-03-16; habitat: Rainforest; **Record Level:** institutionID: Herbarium Togoense; collectionID: Abotsi, K.E.; institutionCode: TOGO; basisOfRecord: Preserved specimen
- **Type status:** Other material. **Occurrence:** recordNumber: AB0294; recordedBy: Abotsi, K.E.; **Taxon:** scientificName: Adiantum vogelii Mett. ex Kuhn.; namePublishedIn: Fil. Afr. 66 (1868), syn., ex Keys., Mém. Ac. Imp. Sci. St. Pétersb. 22(1): 8, 31 (1875); kingdom: Plantae; phylum: Pteridophyta; class: Polypodiopsida; order: Polypodiales; family: Pteridaceae; genus: Adiantum; specificEpithet: vogelii; scientificNameAuthorship: Mett. ex Kuhn.; **Location:** continent: Africa; country: Togo; countryCode: TG; locality: Sérégbéné; verbatimElevation: 519; verbatimSRS: WGS84; decimalLatitude: 7.864763; decimalLongitude: 0.740484; geodeticDatum: WGS84; **Identification:** identifiedBy: Abotsi, K.E.; dateIdentified: 09-17-16; **Event:** eventDate: 09-17-16; habitat: Rainforest; **Record Level:** institutionID: Herbarium Togoense; collectionID: Abotsi, K.E.; institutionCode: TOGO; basisOfRecord: Preserved specimen

##### Ecological interactions

###### Native status

Native

##### Distribution

Zone 4

#### Haplopteris guineensis

(Desv.) E.H.Crane

##### Materials

- **Type status:** Other material. **Occurrence:** recordNumber: AB0474; recordedBy: Abotsi, K.E.; **Taxon:** scientificName: Haplopteris guineensis (Desv.) E.H.Crane; namePublishedIn: Syst. Bot. 22(3): 514 (1998); kingdom: Plantae; phylum: Pteridophyta; class: Polypodiopsida; order: Polypodiales; family: Pteridaceae; genus: Haplopteris; specificEpithet: guineensis; scientificNameAuthorship: (Desv.) E.H.Crane; **Location:** continent: Africa; country: Togo; countryCode: TG; locality: Danyi Dzogbégan; verbatimElevation: 715; verbatimSRS: WGS84; decimalLatitude: 7.232033; decimalLongitude: 0.67768; geodeticDatum: WGS84; **Identification:** identifiedBy: Abotsi, K.E.; dateIdentified: 04-24-17; **Event:** eventDate: 04-24-17; habitat: Rainforest; **Record Level:** institutionID: Herbarium Togoense; collectionID: Abotsi, K.E.; institutionCode: TOGO; basisOfRecord: Preserved specimen
- **Type status:** Other material. **Occurrence:** recordNumber: AB0487; recordedBy: Abotsi, K.E.; **Taxon:** scientificName: Haplopteris guineensis (Desv.) E.H.Crane; namePublishedIn: Syst. Bot. 22(3): 514 (1998); kingdom: Plantae; phylum: Pteridophyta; class: Polypodiopsida; order: Polypodiales; family: Pteridaceae; genus: Haplopteris; specificEpithet: guineensis; scientificNameAuthorship: (Desv.) E.H.Crane; **Location:** continent: Africa; country: Togo; countryCode: TG; locality: Ediwlou; verbatimElevation: 617; verbatimSRS: WGS84; decimalLatitude: 7.409637; decimalLongitude: 0.681109; geodeticDatum: WGS84; **Identification:** identifiedBy: Abotsi, K.E.; dateIdentified: 04-25-17; **Event:** eventDate: 04-25-17; habitat: Rainforest; **Record Level:** institutionID: Herbarium Togoense; collectionID: Abotsi, K.E.; institutionCode: TOGO; basisOfRecord: Preserved specimen

##### Ecological interactions

###### Native status

Native

##### Distribution

Zone 4

#### Salviniales

#### Marsileaceae

#### Marsilea minuta

L.

##### Materials

- **Type status:** Other material. **Occurrence:** recordNumber: AB0001; recordedBy: Abotsi, K.E.; **Taxon:** scientificName: Marsilea minuta L.; namePublishedIn: Mant. Pl. 308 (1771); kingdom: Plantae; phylum: Pteridophyta; class: Polypodiopsida; order: Salviniales; family: Marsileaceae; genus: Marsilea; specificEpithet: minuta; scientificNameAuthorship: L.; **Location:** continent: Africa; country: Togo; countryCode: TG; locality: Dzagblé; verbatimElevation: 16; verbatimSRS: WGS84; decimalLatitude: 6.277842; decimalLongitude: 1.294171; geodeticDatum: WGS84; **Identification:** identifiedBy: Abotsi, K.E.; dateIdentified: 06-04-16; **Event:** eventDate: 06-04-16; habitat: Floating meadow; **Record Level:** institutionID: Herbarium Togoense; collectionID: Abotsi, K.E.; institutionCode: TOGO; basisOfRecord: Preserved specimen
- **Type status:** Other material. **Occurrence:** recordNumber: AB0463; recordedBy: Abotsi, K.E.; **Taxon:** scientificName: Marsilea minuta L.; namePublishedIn: Mant. Pl. 308 (1771); kingdom: Plantae; phylum: Pteridophyta; class: Polypodiopsida; order: Salviniales; family: Marsileaceae; genus: Marsilea; specificEpithet: minuta; scientificNameAuthorship: L.; **Location:** continent: Africa; country: Togo; countryCode: TG; locality: Afito; verbatimElevation: 28; verbatimSRS: WGS84; decimalLatitude: 6.75203; decimalLongitude: 1.592489; geodeticDatum: WGS84; **Identification:** identifiedBy: Abotsi, K.E.; dateIdentified: 01-18-17; **Event:** eventDate: 01-18-17; habitat: Flooded savannah; **Record Level:** institutionID: Herbarium Togoense; collectionID: Abotsi, K.E.; institutionCode: TOGO; basisOfRecord: Preserved specimen
- **Type status:** Other material. **Occurrence:** recordNumber: AB0002; recordedBy: Abotsi, K.E.; **Taxon:** scientificName: Marsilea minuta L.; namePublishedIn: Mant. Pl. 308 (1771); kingdom: Plantae; phylum: Pteridophyta; class: Polypodiopsida; order: Salviniales; family: Marsileaceae; genus: Marsilea; specificEpithet: minuta; scientificNameAuthorship: L.; **Location:** continent: Africa; country: Togo; countryCode: TG; locality: Dzagblé; verbatimElevation: 16; verbatimSRS: WGS84; decimalLatitude: 6.277997; decimalLongitude: 1.294018; geodeticDatum: WGS84; **Identification:** identifiedBy: Abotsi, K.E.; dateIdentified: 06-04-16; **Event:** eventDate: 06-04-16; habitat: Floating meadow; **Record Level:** institutionID: Herbarium Togoense; collectionID: Abotsi, K.E.; institutionCode: TOGO; basisOfRecord: Preserved specimen

##### Ecological interactions

###### Native status

Native

##### Distribution

Zone 5

#### Salviniaceae

#### Azolla pinnatavar.africana

(Desv.) R.M.K. Saunders & K. Fowler

##### Materials

- **Type status:** Other material. **Occurrence:** recordNumber: AB0458; recordedBy: Abotsi, K.E.; **Taxon:** scientificName: Azolla pinnata var. africana (Desv.) R.M.K. Saunders & K. Fowler; namePublishedIn: J. Linn. Soc. Bot. 109(3): 351 (1992); kingdom: Plantae; phylum: Pteridophyta; class: Polypodiopsida; order: Salviniales; family: Salviniaceae; genus: Azolla; specificEpithet: pinnata; infraspecificEpithet: africana; taxonRank: Variety; scientificNameAuthorship: (Desv.) R.M.K. Saunders & K. Fowler; **Location:** continent: Africa; country: Togo; countryCode: TG; locality: Gbowoulé; verbatimElevation: 96; verbatimSRS: WGS84; decimalLatitude: 6.937068; decimalLongitude: 1.524374; geodeticDatum: WGS84; **Identification:** identifiedBy: Abotsi, K.E.; dateIdentified: 01-14-17; **Event:** eventDate: 01-14-17; habitat: Floating meadow; **Record Level:** institutionID: Herbarium Togoense; collectionID: Abotsi, K.E.; institutionCode: TOGO; basisOfRecord: Preserved specimen
- **Type status:** Other material. **Occurrence:** recordNumber: AB0534; recordedBy: Abotsi, K.E.; **Taxon:** scientificName: Azolla pinnata var. africana (Desv.) R.M.K. Saunders & K. Fowler; namePublishedIn: J. Linn. Soc. Bot. 109(3): 351 (1992); kingdom: Plantae; phylum: Pteridophyta; class: Polypodiopsida; order: Salviniales; family: Salviniaceae; genus: Azolla; specificEpithet: pinnata; infraspecificEpithet: africana; taxonRank: Variety; scientificNameAuthorship: (Desv.) R.M.K. Saunders & K. Fowler; **Location:** continent: Africa; country: Togo; countryCode: TG; locality: Tchékpo Dédékpo; verbatimElevation: 32; verbatimSRS: WGS84; decimalLatitude: 6.50838; decimalLongitude: 1.3388016638889; geodeticDatum: WGS84; **Identification:** identifiedBy: Abotsi, K.E.; dateIdentified: 07-01-17; **Event:** eventDate: 07-01-17; habitat: Floating meadow; **Record Level:** institutionID: Herbarium Togoense; collectionID: Abotsi, K.E.; institutionCode: TOGO; basisOfRecord: Preserved specimen
- **Type status:** Other material. **Occurrence:** recordNumber: AB0611; recordedBy: Abotsi, K.E.; **Taxon:** scientificName: Azolla pinnata var. africana (Desv.) R.M.K. Saunders & K. Fowler; namePublishedIn: J. Linn. Soc. Bot. 109(3): 351 (1992); kingdom: Plantae; phylum: Pteridophyta; class: Polypodiopsida; order: Salviniales; family: Salviniaceae; genus: Azolla; specificEpithet: pinnata; infraspecificEpithet: africana; taxonRank: Variety; scientificNameAuthorship: (Desv.) R.M.K. Saunders & K. Fowler; **Location:** continent: Africa; country: Togo; countryCode: TG; locality: Bokpokor; verbatimElevation: 40; verbatimSRS: WGS84; decimalLatitude: 6.258994; decimalLongitude: 1.122876; geodeticDatum: WGS84; **Identification:** identifiedBy: Abotsi, K.E.; dateIdentified: 11-26-17; **Event:** eventDate: 11-26-17; habitat: Floating meadow; **Record Level:** institutionID: Herbarium Togoense; collectionID: Abotsi, K.E.; institutionCode: TOGO; basisOfRecord: Preserved specimen

##### Ecological interactions

###### Native status

Native

##### Distribution

Zone 5

#### Salvinia auriculata

Aubl.

##### Materials

- **Type status:** Other material. **Occurrence:** recordNumber: AB0469; recordedBy: Abotsi, K.E.; **Taxon:** scientificName: Salvinia auriculata Aubl.; namePublishedIn: Hist. Pl. Guiane 2: 969, t. 367 (1775); kingdom: Plantae; phylum: Pteridophyta; class: Polypodiopsida; order: Salviniales; family: Salviniaceae; genus: Salvinia; specificEpithet: auriculata; scientificNameAuthorship: Aubl.; **Location:** continent: Africa; country: Togo; countryCode: TG; locality: Afito; verbatimElevation: 30; verbatimSRS: WGS84; decimalLatitude: 6.751598; decimalLongitude: 1.590756; geodeticDatum: WGS84; **Identification:** identifiedBy: Abotsi, K.E.; dateIdentified: 02-02-17; **Event:** eventDate: 02-02-17; habitat: Floating meadow; **Record Level:** institutionID: Herbarium Togoense; collectionID: Abotsi, K.E.; institutionCode: TOGO; basisOfRecord: Preserved specimen
- **Type status:** Other material. **Occurrence:** recordNumber: AB0470; recordedBy: Abotsi, K.E.; **Taxon:** scientificName: Salvinia auriculata Aubl.; namePublishedIn: Hist. Pl. Guiane 2: 969, t. 367 (1775); kingdom: Plantae; phylum: Pteridophyta; class: Polypodiopsida; order: Salviniales; family: Salviniaceae; genus: Salvinia; specificEpithet: auriculata; scientificNameAuthorship: Aubl.; **Location:** continent: Africa; country: Togo; countryCode: TG; locality: Afito; verbatimElevation: 28; verbatimSRS: WGS84; decimalLatitude: 6.75203; decimalLongitude: 1.592489; geodeticDatum: WGS84; **Identification:** identifiedBy: Abotsi, K.E.; dateIdentified: 02-03-17; **Event:** eventDate: 02-03-17; habitat: Flooded savannah; **Record Level:** institutionID: Herbarium Togoense; collectionID: Abotsi, K.E.; institutionCode: TOGO; basisOfRecord: Preserved specimen

##### Ecological interactions

###### Native status

Native

##### Distribution

Zone 5

#### Salvinia nymphellula

Desv.

##### Materials

- **Type status:** Other material. **Occurrence:** recordNumber: AB0462; recordedBy: Abotsi, K.E.; **Taxon:** scientificName: Salvinia nymphellula Desv.; namePublishedIn: Mem. Soc. Linn. Paris 6, 2: 177 (1827); kingdom: Plantae; phylum: Pteridophyta; class: Polypodiopsida; order: Salviniales; family: Salviniaceae; genus: Salvinia; specificEpithet: nymphellula; scientificNameAuthorship: Desv.; **Location:** continent: Africa; country: Togo; countryCode: TG; locality: Afito; verbatimElevation: 27; verbatimSRS: WGS84; decimalLatitude: 6.753214; decimalLongitude: 1.599899; geodeticDatum: WGS84; **Identification:** identifiedBy: Abotsi, K.E.; dateIdentified: 01-18-17; **Event:** eventDate: 01-18-17; habitat: Floating meadow; **Record Level:** institutionID: Herbarium Togoense; collectionID: Abotsi, K.E.; institutionCode: TOGO; basisOfRecord: Preserved specimen
- **Type status:** Other material. **Occurrence:** recordNumber: AB0472; recordedBy: Abotsi, K.E.; **Taxon:** scientificName: Salvinia nymphellula Desv.; namePublishedIn: Mem. Soc. Linn. Paris 6, 2: 177 (1827); kingdom: Plantae; phylum: Pteridophyta; class: Polypodiopsida; order: Salviniales; family: Salviniaceae; genus: Salvinia; specificEpithet: nymphellula; scientificNameAuthorship: Desv.; **Location:** continent: Africa; country: Togo; countryCode: TG; locality: Tchékpo Dédékpo; verbatimElevation: 31; verbatimSRS: WGS84; decimalLatitude: 6.520226; decimalLongitude: 1.348513; geodeticDatum: WGS84; **Identification:** identifiedBy: Abotsi, K.E.; dateIdentified: 02-04-17; **Event:** eventDate: 02-04-17; habitat: Floating meadow; **Record Level:** institutionID: Herbarium Togoense; collectionID: Abotsi, K.E.; institutionCode: TOGO; basisOfRecord: Preserved specimen

##### Ecological interactions

###### Native status

Native

##### Distribution

Zone 5

#### Schizaeales

#### Anemiaceae

#### Anemia sessilis

(Jeanp.) C.Chr.

##### Materials

- **Type status:** Other material. **Occurrence:** recordNumber: AB0541; recordedBy: Abotsi, K.E.; **Taxon:** scientificName: Anemia sessilis (Jeanp.) C.Chr.; namePublishedIn: Repert. Spec. Nov. Regni Veg. 9: 371 (1911); kingdom: Plantae; phylum: Pteridophyta; class: Polypodiopsida; order: Schizaeales; family: Anemiaceae; genus: Anemia; specificEpithet: sessilis; scientificNameAuthorship: (Jeanp.) C.Chr.; **Location:** continent: Africa; country: Togo; countryCode: TG; locality: Défalé; verbatimElevation: 312; verbatimSRS: WGS84; decimalLatitude: 9.917837; decimalLongitude: 1.104178; geodeticDatum: WGS84; **Identification:** identifiedBy: Abotsi, K.E.; dateIdentified: 07-11-17; **Event:** eventDate: 07-11-17; habitat: Dry dense forest; **Record Level:** institutionID: Herbarium Togoense; collectionID: Abotsi, K.E.; institutionCode: TOGO; basisOfRecord: Preserved specimen
- **Type status:** Other material. **Occurrence:** recordNumber: AB0559; recordedBy: Abotsi, K.E.; **Taxon:** scientificName: Anemia sessilis (Jeanp.) C.Chr.; namePublishedIn: Repert. Spec. Nov. Regni Veg. 9: 371 (1911); kingdom: Plantae; phylum: Pteridophyta; class: Polypodiopsida; order: Schizaeales; family: Anemiaceae; genus: Anemia; specificEpithet: sessilis; scientificNameAuthorship: (Jeanp.) C.Chr.; **Location:** continent: Africa; country: Togo; countryCode: TG; locality: Agaradè; verbatimElevation: 748; verbatimSRS: WGS84; decimalLatitude: 9.290947; decimalLongitude: 1.212542; geodeticDatum: WGS84; **Identification:** identifiedBy: Abotsi, K.E.; dateIdentified: 07-13-17; **Event:** eventDate: 07-13-17; habitat: Wooded savannah; **Record Level:** institutionID: Herbarium Togoense; collectionID: Abotsi, K.E.; institutionCode: TOGO; basisOfRecord: Preserved specimen
- **Type status:** Other material. **Occurrence:** recordNumber: AB0562; recordedBy: Abotsi, K.E.; **Taxon:** scientificName: Anemia sessilis (Jeanp.) C.Chr.; namePublishedIn: Repert. Spec. Nov. Regni Veg. 9: 371 (1911); kingdom: Plantae; phylum: Pteridophyta; class: Polypodiopsida; order: Schizaeales; family: Anemiaceae; genus: Anemia; specificEpithet: sessilis; scientificNameAuthorship: (Jeanp.) C.Chr.; **Location:** continent: Africa; country: Togo; countryCode: TG; locality: Alédjo Kadara; verbatimElevation: 796; verbatimSRS: WGS84; decimalLatitude: 9.246814; decimalLongitude: 1.202418; geodeticDatum: WGS84; **Identification:** identifiedBy: Abotsi, K.E.; dateIdentified: 07-13-17; **Event:** eventDate: 07-13-17; habitat: Wooded savannah; **Record Level:** institutionID: Herbarium Togoense; collectionID: Abotsi, K.E.; institutionCode: TOGO; basisOfRecord: Preserved specimen

##### Ecological interactions

###### Native status

Native

##### Distribution

Zone 2

#### Lygodiaceae

#### Lygodium microphyllum

(Cav.) R. Br.

##### Materials

- **Type status:** Other material. **Occurrence:** catalogNumber: 12410; recordNumber: 7864; recordedBy: Brunel, J.-F.; **Taxon:** scientificName: Lygodium microphyllum (Cav.) R. Br.; namePublishedIn: Prodr. Fl. Nov. Holland. 162 (1810); kingdom: Plantae; phylum: Pteridophyta; class: Polypodiopsida; order: Schizaeales; family: Lygodiaceae; genus: Lygodium; specificEpithet: microphyllum; scientificNameAuthorship: (Cav.) R. Br.; **Location:** continent: Africa; country: Togo; countryCode: TG; locality: Danyi Dzogbégan; verbatimElevation: 786; verbatimSRS: WGS84; decimalLatitude: 7.236561041; decimalLongitude: 0.688195106; geodeticDatum: WGS84; **Identification:** identifiedBy: Brunel, J.-F.; dateIdentified: /06/1983; **Event:** eventDate: /06/1983; habitat: Rainforest; **Record Level:** institutionID: Herbarium Togoense; collectionID: Brunel, J.-F.; institutionCode: TOGO; basisOfRecord: Preserved specimen

##### Ecological interactions

###### Native status

Native

##### Distribution

Zone 4

## Analysis

**Figure 2.**
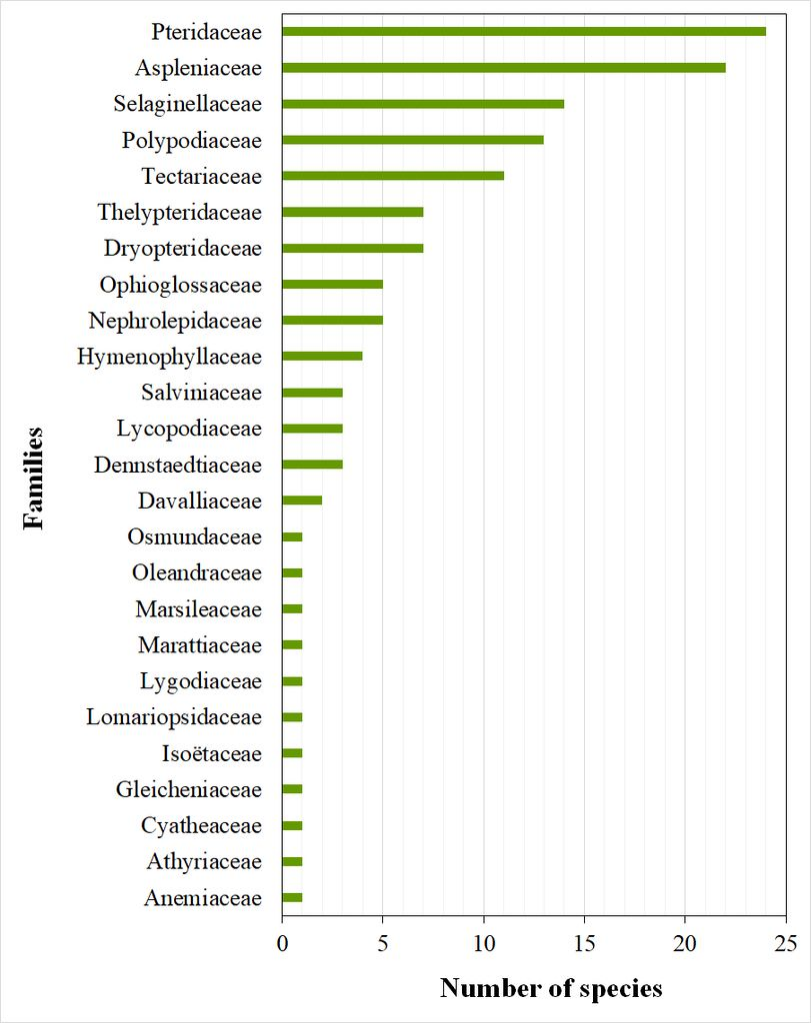
Species diversity of Pteridophytes families occurring in Togo

The current diversity of Pteridophytes in Togo is 134 species including 122 spontaneous species (native or naturalised) and 12 species obviously introduced mainly for horticultural purposes. These species are divided into 53 genera, 25 families, 12 orders, 4 subclasses and 2 classes. The Polypodiales and the Selaginellales are the most diversified orders with respectively 97 and 14 species. The most representative families are Pteridaceae (24 species), Aspleniaceae (22 species) and Selaginellaceae (14 species). In contrast, 11 families are represented by only 1 species each (Fig. 2).

## Discussion

With a diversity now estimated at 134 species including 12 exotics, the present study provides the first checklist of Togolese Pteridophytes based on an integrative approach combining newly collected specimens and data from the literature, mostly by Alston (1959), Roux (2009) and Abotsi et al. (2015). The checklist for the country is consequently complemented by 12 exotic species and 61 spontaneous species. Alston (1959), Roux (2009)

Regarding the species previously mentioned for the country, it appears that seven species, listed by Roux (2009), were not confirmed by any specimens, neither in the herbaria consulted, nor in the GBIF databases. These species are *Crepidomanes minutum* (Blume) K.Iwats. (*as Crepidomanes mannii* (Hook) J.P.Roux; see Dubuisson et al. 2013), *Didymoglossum erosum* (Willd.) J.P.Roux, *Asplenium blastophorum* Hieron., *Asplenium uhligii* Hieron, *Oleandra distenta* Kunze, *Triplophyllum heudelotii* Pic.Serm and *Triplophyllum subquinquefidum* (P.Beauv.) Pic.Serm. Even if it is possible that Roux (2009) mentioned the occurence of these species by extrapolation of their known and documented ranges, it is also possible that specimens do support this occurence without being available in online databases. In addition, we were also unable to confirm their presence in the new samples collected in the context of our recent collections which were, however, focused on ferns between 2013 and 2017 for this study. This could result from a drastic decrease in the local range of these species following the loss of their primary habitat and their confinement in forest relics. Indeed, the increasing deforestation in Togo (Adjossou 2009, Kokou 1998, Nadjombe 1992), which has been underway for several decades, has as its main consequence, the degradation and fragmentation of habitats, which generally remain only in the form of forest relics (Kokou and Caballé 2000) in more or less inaccessible places. Our investigations did not reach some very difficult to access sites and these may therefore abound with interesting biodiversity.

By comparing Togo's ecological and climatic conjectures with its neighbouring regions, there may still be spontaneous species to discover. Indeed, Pteridophytes from the neighbouring regions of Togo are estimated at 42 species, 165 species and 25 species respectively for Benin (Akoègninou et al. 2006), Ghana (Alston 1959, Hassler and Schmitt 2017) and Burkina Faso (Thiombiano et al. 2012).

However, it is very difficult to confirm the number of species provided by Fousseni et al. (2014), indicating a local diversity of 126 species including 15 exotics, since the authors did not present a list of species for this purpose. Until 2013, at the beginning of this study, Pteridophytes’ preserved specimens available at the herbarium TOGO do not comprise more than 20 species. Indeed, so far, specimens of plants collected in the country were not always deposited in the institutions provided for this purpose, which also prompted a resumption of field collection for this study.

Finally, since the control mechanisms for plant species in transit to and across Togo national borders are not sufficiently rigorous, it is possible that the list of introduced Pteridophytes species in Togo (12 at all), accidentally or knowingly for horticultural purposes, is incomplete. Ongoing collections of biological data from horticulturists and custom services would greatly improve this list.

### Conclusion

This study estimates the diversity of Pteridophytes in Togo to 134 species, comprising 122 spontaneous species and 12 introduced species for horticultural purposes. This flora is very diverse at supra-specific levels, with 53 genera divided into 24 families and 12 orders, from which 3 belong to Lycophytes. It provides 73 new species for the Togolese flora.

Although future investigations in both isolated areas and protected areas might reveal additional taxa, this study resulted in the first checklist of pteridophytes of Togo, that should set the stage for a Flora of the Pteridophytes in Togo which is still unavailable to date.
